# Insights into Decoupled Solar Energy Conversion and Charge Storage in a 2D Covalent Organic Framework for Solar Battery Function

**DOI:** 10.1021/jacs.4c17642

**Published:** 2025-04-28

**Authors:** Bibhuti Bhusan Rath, Laura Fuchs, Friedrich Stemmler, Andrés Rodríguez-Camargo, Yang Wang, Maximilian F. X. Dorfner, Johann Olbrich, Joris van Slageren, Frank Ortmann, Bettina V. Lotsch

**Affiliations:** † Nanochemistry Department, 28326 Max Planck Institute for Solid State Research, Heisenbergstraße 1, 70569 Stuttgart, Germany; ‡ Department of Chemistry, TUM School of Natural Sciences, Technische Universität München, Garching b., 85748 München, Germany; § Institute of Physical Chemistry, 9149 University of Stuttgart, Pfaffenwaldring 55, 70569 Stuttgart, Germany; ∥ Department of Chemistry, University of Stuttgart, Pfaffenwaldring 55, 70569 Stuttgart, Germany; ⊥ Department of Chemistry, Ludwig-Maximilians-Universität (LMU), Butenandtstr. 5-13, 81377 Munich, Germany; # E-Conversion and Center for Nanoscience, Lichtenbergstrasse 4a, 85748 Garching, Germany

## Abstract

Decoupling solar energy conversion and storage in a single material offers a great advantage for off-grid applications. Herein, we disclose a two-dimensional naphthalenediimide (NDI)-based covalent organic framework (COF) exhibiting remarkable solar battery performance when used as a photoanode. Light-induced radicals are stabilized within the framework for several hours, offering on-demand charge extraction for electrical energy production. Our study reveals mechanistic insights into the long-term charge stabilization using optical spectroscopy and (photo)­electrochemical measurements, in conjunction with density functional theory (DFT) simulations. Among several solvents, water provides the best dielectric screening and energetically favorable proton exchange to stabilize photoinduced radicals for more than 48 h without the need for additional metal cations. This study provides fundamental insights into the optoionic charge storage mechanism in NDI-COF, while introducing a highly tunable, nanoporous material platform that surpasses related materials, such as carbon nitrides, metal–organic frameworks (MOFs), or metal oxides, in terms of charge storage capacity. This study opens new perspectives for the design of optoionic charge-storing materials and the direct storage of solar energy to overcome the intermittency of solar irradiation.

## Graphical abstract

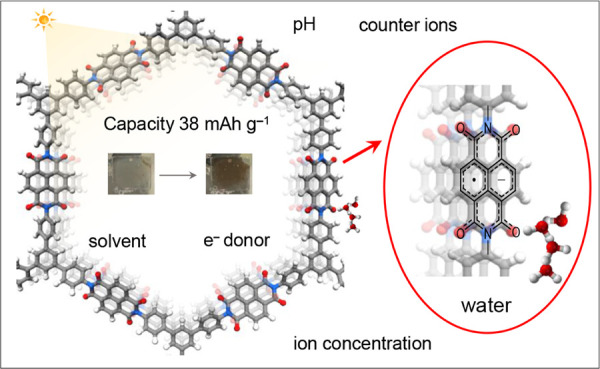

## 1 Introduction

In the pursuit of mitigating our reliance on fossil fuels, harnessing solar energy as an abundant, renewable energy source is a key element of today’s energy infrastructure. A decoupled model of photoelectric conversion and charge storage, akin to natural photosynthesis, would be highly desirable to “buffer” the intermittency of solar energy.^1^ Despite major progress made in photovoltaics, energy storage in external batteries requires decoupling of energy conversion and storage, resulting in energy losses and higher system costs. The effective storage of excess energy can be achieved by integrating the photoconversion system and the energy storage component within a single device.^2,3^ On the material level, coupled light harvesting and charge storage properties have been identified in a small set of materials, including carbon nitrides,^4–6^ MOFs,^7,8^ perovskites,^9^ photosensitizer-polyoxometalate dyads,^10^ and metal oxides,^11–14^ some of which are used in faradaic junctions.^15^ However, so far, the longevity and capacity of charge storage are not large enough to meet substantial off-grid energy demands. In spite of such challenges, electrodes composed of a single, bifunctional material can avoid the complex fabrication of multimaterial junctions and overcome losses pertaining to electron injection and transport. In this regard, poly­(heptazine imide) (PHI)-type 2D ionic carbon nitride has been a benchmark material for bifunctional photoanodes and on-demand charge extraction for “dark” (i.e., time-delayed) hydrogen evolution, thus setting the stage for the exploration of other framework structures such as MOFs and COFs.^2,4–6,16^ Above-bandgap photointercalation of metal ions effectively screens the photoaccumulated electrons, which are trapped on the heptazine backbone, while the photogenerated holes are extracted by a sacrificial electron donor.^5,17^ However, the intrinsically low conductivity of PHI-type carbon nitrides hampers the charge transfer and collection process, which translates into low capacity utilization. Moreover, the limitation of compositional tunability of the molecular backbone of carbon nitrides severely restricts their variability and functionality. This bottleneck can possibly be overcome by reticular framework materials with superior light harvesting and charge storing properties.

COFs have emerged as a subclass of crystalline porous materials displaying large structural diversity and molecular-level tunability, which allows target-specific design for optoelectronics and energy applications.^18–20^ Previously, Wang and coworkers demonstrated the coupling of photoinduced electron transfer and reversible de/intercalation of cations for direct solar-to-electrochemical energy storage in functional COF photoelectrodes.^21–23^ While this represents a huge leap forward in the design of reticular solar battery materials, the trifecta of high capacity, long-term charge storage, and on-demand carrier extraction in COFs remains largely elusive to date. In addition, deeper insights into the mechanism of charge trapping are critical for the rational design of charge storage materials, which further pushes the performance of existing systems.

We have selected a two-dimensional naphthalene diimide (NDI)-based COF, which acts as an electron reservoir to accommodate photogenerated electrons after the quenching of holes by a sacrificial electron donor, based on the following considerations. Formation of NDI radicals has been documented in various compounds using chemical, photochemical, and electrochemical techniques.^24–28^ NDI units are found to be promising electron acceptors in organic field-effect transistors (OFETs) and complex supramolecular structures.^25^ Therefore, NDI represents an excellent benchmark system to study light-induced energy storage in COFs. In addition, imide-based COFs are known to exhibit excellent (photo)­electrochemical properties and show exceptional framework stability.^29,30^ In this work, we explore the photoinduced charge trapping behavior and on-demand release of accumulated electrons in a donor–acceptor-type NDI-COF as an efficient aqueous solar battery. With a capacity of 38 mAh g^–1^, NDI-COF supersedes the charge storage capacity of contemporary optoionic materials. Moreover, obtaining a fundamental understanding of the charge trapping mechanism is a prerequisite for the development of materials with improved performance. Hence, we present a holistic study revealing the role of solvents, electron donors, and external ions in determining the long-term charge storage capability and overall capacity. Our findings indicate that charge trapping in NDI-COF is mediated by dielectric screening via dipolar interactions and hydrogen bonding, thus rendering water the solvent of choice for long-term charge trapping. On the contrary, the type of charge compensating cation and pH seem to have less influence on the charge trapping efficiency. DFT simulations indicate that NDI π-interactions facilitate efficient stabilization and delocalization of the excess electronic charge of the NDI^•–^ radicals over the NDI moieties and COF backbone, making charge screening a global, rather than local, property of the system. Apart from demonstrating the superior charge storage performance of the NDI-COF, this study provides fundamental insights into the optoionic charge storage mechanism operative in COFs, thus carving out design principles for long-term charge stabilization.

## 2 Results and Discussion

### 2.1 Synthesis and Characterization

NDI-COF was synthesized using a modified literature method by reacting 1,4,5,8–naphthalenetetracarboxylic dianhydride (NTCDA) with 1,3,5–tris­(4–aminophenyl)­benzene (TAPB) under solvothermal conditions (more details in Figure S1). Adequate washing and Soxhlet extraction followed by supercritical CO_2_ activation yielded NDI-COF as a light brown solid. The appearance of characteristic bands (at 1716 and 1677 cm^–1^ for the asymmetric and symmetric stretching vibrations of the C=O group, respectively, and at 1341 cm^–1^ for the C–N–C stretching vibration) in Fourier transform infrared spectroscopy (FTIR) analysis confirmed the formation of the six-membered imide ring. Furthermore, the N–H or C=O bands from the amino and anhydride monomers disappeared from the spectrum, thus confirming quantitative imide formation (Figure S2). The solid-state ^13^C cross-polarization magic angle spinning NMR (^13^C–CP/MAS NMR) spectrum shows a signal at 163.4 ppm corresponding to the carbonyl carbon of the six-membered imide ring, while the overlapping signals centered at 141.8 and 128.6 ppm correspond to the aromatic carbons from the naphthalenyl and phenyl moieties (Figure S3). Pawley refinement of the powder X-ray diffraction (PXRD) pattern shows good agreement with the metrics of the simulated structure. Note that the broadness of the PXRD reflections often makes it possible to get acceptable agreement factors for a range of lattice parameters after Pawley refinement.^31^ We therefore adapted a previously published structure, which takes into account torsion of the building blocks and a trigonal AA stacking.^32^ Pawley refinement produced a hexagonal unit cell (P3̅1*m*) with *a* = *b* = 37.073 Å, *c* = 3.88 Å, α = β = 90°, and γ = 120°, with refinement results of *R*_wp_ = 2.65% and *R*_p_ = 2.08% (Figure 1a). An eclipsed stacked model of NDI-COF and the molecular NDI unit are presented in Figure 1b. The reproducibility of NDI-COF synthesis by the solvothermal method was verified across multiple batches (Figures S4–S6). Ar adsorption measurements at 77 K yield a Brunauer–Emmett–Teller (BET) surface area of 982 m^2^ g^–1^ and a total pore volume of 1.452 cm^3^ g^–1^ (Figure S7). Scanning electron microscopy (SEM) reveals a microcrystalline morphology with typical domain sizes of 100 ± 32 nm (Figure S8). Transmission electron microscopy (TEM) shows flake-like particle morphology, and fast-Fourier transforms (FFTs) from selected areas give a *d*-spacing of ∼3.0 nm (Figures 1c and S9), which is consistent with the theoretical pore size of 3.12 nm (Figure S10). DFT simulations indicate that the NDI units stack on top of each other. Although the NDI fragments are somewhat rotated out of plane, deviating from perfect cofacial eclipsed stacking, the electronic coupling of the aromatic units leads to strong band dispersion in the π stacking direction, i.e., Γ **→** Α (Figure 1d), which is a driving force for charge carrier delocalization along the stacking direction.

### 2.2 Charge Storage Properties of NDI-COF

First, to assess the accessible redox processes in the NDI-COF, cyclic voltammetry was performed in a three-electrode system with a carbon paper electrode, an Ag/AgCl reference electrode, and a platinum counter electrode in 1 M KCl aqueous electrolyte (Figure S11). Well-separated one- and two-electron-reduced species of the NDI core are observed at redox potentials of −0.55 V (NDI^0/•–^) and −0.72 V (NDI^•–/2–^) versus Ag/AgCl, respectively (Figures S12 and S13). Two pairs of redox peaks (denoted as O1/R1 and O2/R2) indicate two-step intercalation of alkali metal ions onto the framework. However, when the CV was performed on an FTO electrode, NDI^0/•–^ and NDI^•–/2–^ redox couples were no longer distinguishable, yielding only a broad peak (R and O) at −0.6 V vs Ag/AgCl (Figures 2a and S14). Sluggish interfacial electron transfer, coupled with ohmic resistance in the film and slow ion transport at the film-electrolyte interface, could cause such behavior, which is also dependent on the specific choice of solvent and electrolyte.^33,34^ With increasing scan rate (0.1 mV s^–1^ to 1 mV s^–1^), the polarization potentials between the cathodic peak and anodic peak slightly increase, while the peak shape remains the same (Figure S15). The charge storage kinetics can be determined by the exponent *b*-value in eq 1, where *i* refers to the current density, *v* represents the scan rate, and *a* and *b* are constants.^30,35^

1 $$i=av^{b}$$

**Figure 1:**
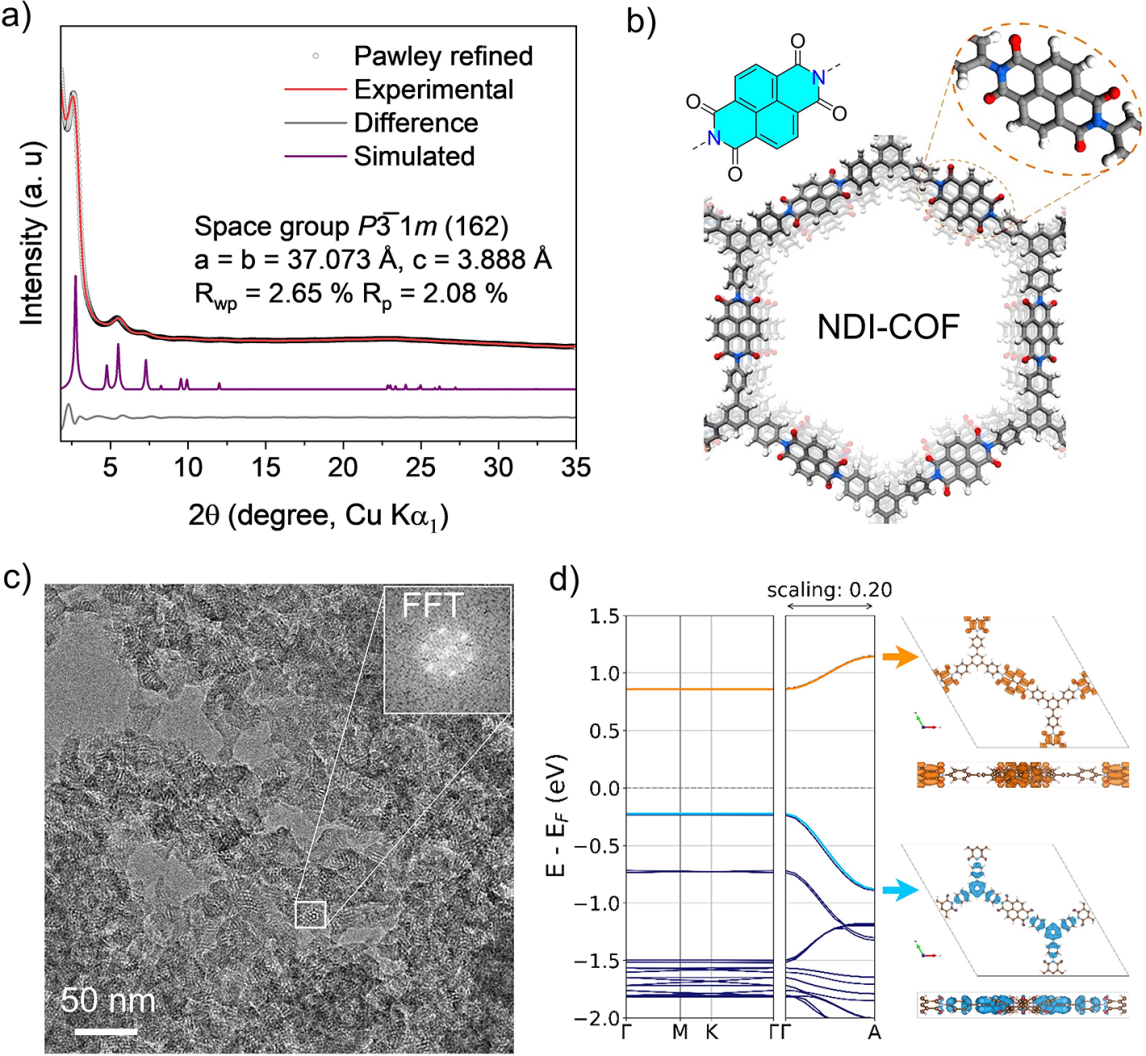
(a) PXRD patterns of NDI-COF are shown with the experimental in red, Pawley refined data as black circles, calculated trigonal structure based on AA stacking in violet, and the difference (observed minus refined profiles) in gray. (b) Simulated structure of NDI-COF showing π–π stacking of 2D layers. A close-up of a molecular NDI unit is shown for clarity. (c) TEM image and FFT of the selected region (inset) of NDI-COF. (d) Band structure, from DFT simulations with rescaled band gap to match hybrid DFT level, indicating strong band dispersion in π stacking direction with visualization of localization of charge densities for HOMO (blue) and LUMO (orange) bands in top and side views.

Typically, a *b*-value of 0.5 indicates a diffusion-controlled process, while a *b*-value of 1.0 suggests a surface-controlled or (pseudo)­capacitive process. The scan-rate dependence of the current density for R and O is plotted in Figure 2b and linear fitting of the slopes gives *b*-values of 0.81 and 0.78, respectively. These *b*-values (>0.5) are indicative of charge storage dominated by a (pseudo)­capacitive or surface-controlled process rather than solid-state (i.e., bulk) diffusion of alkali metal ions.^36,37^ To quantify the (pseudo)­capacitive contribution to charge storage, we employed the method developed by Dunn, according to which the (pseudo)­capacitive contribution (*k*_1_ *v*) and diffusion-controlled contribution (*k*_2_ *v* ^0.5^) in the CV curves to the current response *i* at a fixed potential *V* are separated according to eq 2 (Figure 2c).

2 $$i=k_{1}v+k_{2}v^{0.5}$$

The role of the (pseudo)­capacitive contribution increases with increasing scan rate, yielding (pseudo)­capacitive contributions at 0.1, 0.2, 0.4, 0.6, 0.8, and 1 mV s^–1^ of 70, 76, 82, 85, 87, and 89%, respectively (Figure 2d). This is consistent with the highly porous nature of the COF film and the correspondingly high surface area, which allows for the rapid access of solvent species (i.e., charge-compensating ions) into the COF interior, thus shifting the charge storage in NDI-COF from diffusion-controlled to (pseudo)­capacitive. To verify the stability of the NDI-COF film on the FTO electrode, CV was performed for 1500 cycles at a scan rate of 10 mV s^–1^, revealing that the NDI-COF electrode shows good long-term stability and cycling performance with a capacity retention of 50% (Figure S16). Despite a decrease in the crystallinity, the overall structural integrity of NDI-COF remains intact (Figure S17). This is crucial for the solar battery application, which will be discussed further below.

Accessing the different redox states in the NDI-COF film on FTO resulted in a visual color change from light yellow to brown. The redox states addressable by electrochemical reduction were characterized by *in situ* UV–Vis spectroelectrochemical measurements (Figure 2e). The pristine NDI-COF film shows characteristic π–π* bands of the NDI core at 344 and 365 nm (also at a potential of 0 V vs Ag/AgCl). A small band at 433 nm possibly corresponds to the donor-to-acceptor (TAPB to NDI) charge transfer band.^38,39^ As the cathodic scan proceeds, new bands at 473, 602, 676, and 750 nm corresponding to the NDI^•–^ radical species appear.^40,41^ At a more negative potential, a different ratio of NDI^•–^ and NDI^2–^ species must be present, which, however, is disguised by the indiscernible redox peaks on FTO, as mentioned above. A potential beyond −0.7 V versus Ag/AgCl results in the complete reduction of NDI^•–^ to the dianion NDI^2–^ with electronic transitions at 346 and 372 nm. While the spectral signatures of NDI^2–^ largely resemble those of NDI^•–^, a new peak at 579 nm appears. It is worth noting that NDI^2–^ is less stable compared to NDI^•–^ and that its distinct spectral signature can be detected under continuous Ar purging. As expected, the interconversion of the radical anions in the cathodic scan can be reversed during the anodic scan, corroborating the reversible two-step electrochemical reduction (Figure S18). To gain a clearer understanding of the different redox states, the NDI-COF film was monitored by *in situ* spectroelectrochemical EPR analysis (Figure 2f). During the cathodic scan, the intensity of EPR spectra increases between the negative bias voltage from 0 V to −0.75 V vs Ag/AgCl, corresponding to the continuous conversion of NDI units to NDI^•–^ radicals. The *g* values at 2.0036(1) are attributed to the unpaired electron of paramagnetic NDI^•–^ species, in line with the reported literature.^30,42^ On further increase in the negative bias above −0.75 V vs Ag/AgCl, a slight decrease in the intensity of the EPR spectra was observed. In principle, the conversion of NDI^•–^ radicals to diamagnetic NDI^2–^ species would result in the suppression of EPR signal intensity. However, due to the lower stability of NDI^2–^ species, a residual EPR signal is attributed to persistent NDI^•–^ radical anions, which corroborates the UV–Vis spectroelectrochemical analysis.

**Figure 2:**
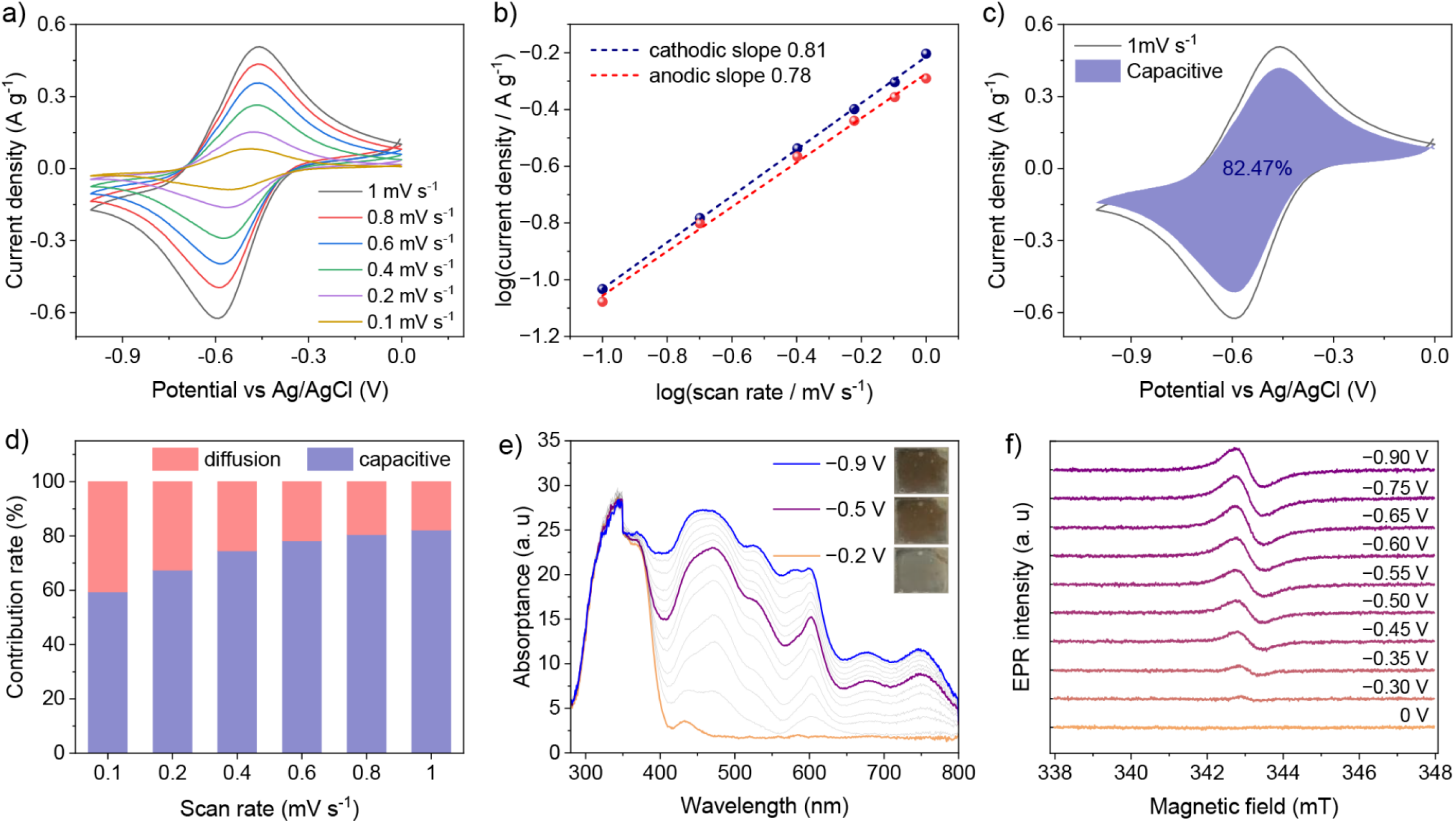
Kinetic analysis of the electrochemical charge storage in NDI-COF. (a) CV curves of the NDI-COF on a FTO electrode at various scan rates. (b) *b*-value as defined in eq 1 for the redox couple. (c) CV curves showing the capacitive contribution of the NDI-COF electrode at a scan rate of 1 mV s^–1^. (d) Comparison of capacitive contribution of NDI-COF at various scan rates. (e) UV–vis spectro-electrochemical analysis of NDI-COF, showing the transformation to the NDI^•–^ and NDI^2–^ species and the resulting color changes during a cathodic scan. (f) *In situ* EPR spectroscopic analysis of NDI-COF shows different redox states during a cathodic scan.

### 2.3 Photoinduced Electron Accumulation

Encouraged by the electrochemical accessibility of the mono- and dianions of NDI in the dark, we probed the photochromic behavior of NDI-COF in an O_2_-free aqueous suspension in the presence of 4-methylbenzyl alcohol (4-MBA) as a sacrificial electron donor (SED). The UV–vis spectrum of the NDI-COF suspension shows vibronic progression (0–1 band around 365 nm and 0–0 band around 385 nm), indicative of characteristic π–π* transitions observed in NDI derivatives.^43^ The bandgap of NDI-COF was calculated to be 3.06 eV (Figure S19). Upon 365 nm UV irradiation (nominal power *P*_nominal_ ≈100 mW cm^–2^), the light yellow suspension gradually transforms to brown with the evolution of new excitations at 490, 545, 618, 688, and 760 nm, corresponding to NDI^•–^ radical species, as observed during the CV measurements (Figure 3a). Under continuous irradiation, the signal for the NDI^•–^ radical reaches a stationary state after 3 min with an isosbestic point at 405 nm and remains stable thereafter, even after the illumination is stopped. It is important to note that photocharging only yields the one-electron-reduced NDI^•–^ radical species, as opposed to electrochemical reduction, where both reduced species (NDI^•–^ and NDI^2–^) can be accessed. Control experiments suggest that the SED is important for hole quenching after exciton generation (Figure S20), whereas the photoelectrons are trapped on the NDI^•–^ radicals. Note that radical stabilization requires an inert atmosphere, and O_2_ exposure causes radical quenching, as seen from the disappearance of the new bands, ultimately yielding the original spectrum, accompanied by color reversal (Figure S21). Radical formation and quenching could be repeated for multiple cycles, indicating the reversibility of photoinduced radical generation and the reusability of NDI-COF for charge storage and subsequent charge extraction (Figure 3b inset).

**Figure 3:**
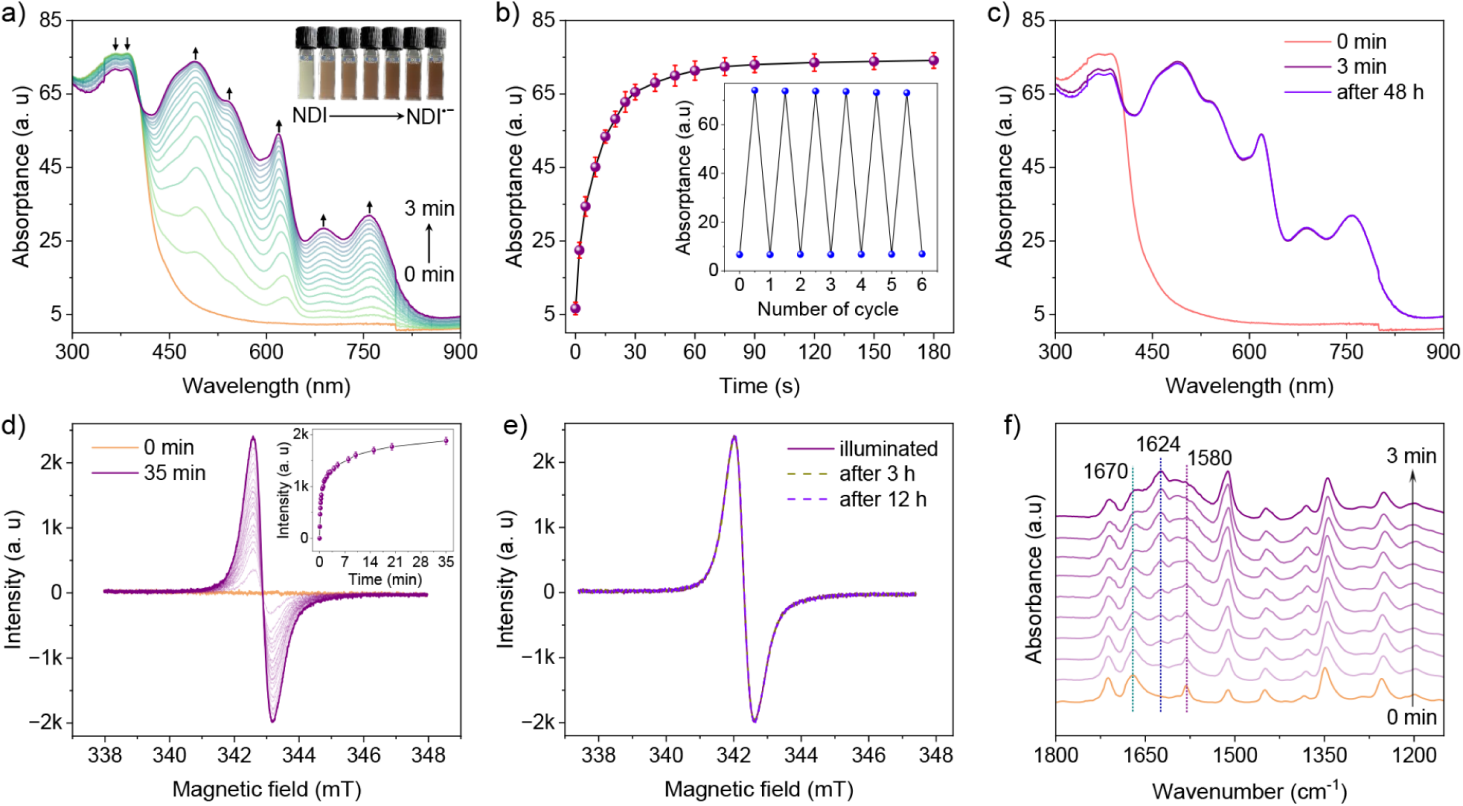
(a) Absorption spectra of an aqueous NDI-COF suspension in 10 mM 4-MBA showing the formation of the NDI^•–^ radical anion upon continuous illumination. (b) Time vs absorptance plot showing the progress of NDI^•–^ radical formation. Inset shows the reversibility and recyclability after O_2_ exposure. (c) Long-term dark stability (up to 48 h) of the NDI^•–^ radical formed after 3 min illumination. (d) EPR spectra monitoring the formation of the photoinduced NDI^•–^ radical. Inset shows the reaction kinetics. (e) Comparison of time evolution of EPR spectra, showing the stability of the NDI^•–^ radical. (f) ATR-IR spectra showing the evolution of the NDI^•–^ radical signal upon illumination.

Under an Ar atmosphere, the photogenerated “trapped” electrons remain stable for more than 48 h, as observed from the unchanged UV–visible absorption spectrum (Figure 3c). A slight reduction in signal intensity is possibly due to the leakage of oxygen into the system. The dark stability of the NDI^•–^ radical is highly relevant for the on-demand release of accumulated photoelectrons for decoupled light and dark (e.g., catalytic) processes, enabling its use in solar batteries and “dark” photocatalysis. The formation and stability of NDI^•–^ could be verified by characteristic signals in the electron paramagnetic resonance (EPR) spectra. It is noteworthy that, in the ground state, a very weak radical signal with a *g* factor of 2.0039 was observed, indicative of spontaneous ground-state partial charge transfer from TAPB to the NDI unit.^44^ Upon continuous irradiation, the signal intensity at *g* = 2.0039(1), corresponding to NDI^•–^ species (in view of the spectroelectrochemical results), increased significantly, originating from unpaired electrons. This is consistent with photoreduced electron trapping (Figure 3d). Double integration of the EPR peaks shows the evolution of NDI^•–^ to a saturation value within 35 min of irradiation (Figure S22), which remained stable thereafter in the dark for 12 h in an inert atmosphere (Figure 3e). Furthermore, the formation of the NDI^•–^ radical anion is expected to change the C=O double bond character of the six-membered imide ring toward a single bond C–O by shifting electron density into the antibonding π*orbital.^45,46^ This change in electron density affects the C–O stretching frequency, which can be monitored by changes in the IR spectra after illumination. The characteristic peaks of the C=O groups of NDI at 1712 cm^–1^ and 1672 cm^–1^ were shifted to lower frequencies of 1670 cm^–1^ and 1624 cm^–1^ in the NDI^•–^ spectrum (Figure 3f). In the normalized ATR-IR spectra, with an increase in illumination time, a new peak at 1624 cm^–1^ corresponding to the C–O stretching frequency gradually appears (Figure 3f). Concurrently, the characteristic imide C=O (1670 cm^–1^) and imide C=C (1580 cm^–1^) peaks become broadened due to changes in the conjugated six-membered ring. Simulated IR absorption spectra corroborate the shift in peak position as a function of charging time, as different ratios of NDI^•–^ radicals are present (Figure S23). Upon O_2_ exposure, the peak at 1624 cm^–1^ (C–O stretching frequency) vanishes, suggesting radical quenching and reversal to the ground state (Figure S24). This process could be reproduced multiple times, indicating that the structural integrity of the NDI-COF is maintained.

### 2.4 Photoelectrochemical Investigation

To further investigate the photoinduced charge accumulation phenomenon, photoelectrochemical experiments were performed on the NDI-COF photoanode using a three-electrode setup with Ag/AgCl as the reference electrode and platinum wire as the counter electrode (Figure 4a and S11). Photocharging was conducted under standard solar illumination (AM 1.5 G, *P*_nominal_ ≈100 mW cm^–2^) from the backside of the photoanode in an O_2_-free aqueous electrolyte. First, we analyzed the accessible open-circuit potential (OCP) and the longevity of stored charges under different conditions. A 120-min illumination in pure water without a dedicated electron donor gives rise to an OCP value of −0.32 V vs Ag/AgCl, possibly due to some photoelectrons in the conduction band or trap states. When 4-MBA is added as SED, the OCP rises to −0.65 V vs Ag/AgCl, accounting for the accumulated electrons in NDI^•–^ by effective hole quenching (Figure 4b).

As seen in previous experiments, the color of the electrode changed from light yellow to brown after attaining this photopotential, which remains stable for more than 48 h after the irradiation is stopped. This demonstrates the (pseudo)­capacitive storage of electrons in a potential window more negative than −0.35 V vs Ag/AgCl. The OCP decays faster in the presence of an electron scavenger such as O_2_, which prevents the accumulation of photoelectrons and their stability after terminating the irradiation. Moreover, photocurrent measurements were performed to probe photoelectron generation per second in the absence and presence of 4-MBA. The transient photocurrent spectra show an accelerated charging kinetics in NDI-COF when the donor is present, which is reflected by an enhanced photocurrent response (10 times higher) compared with that in pure water. Upon illumination, the photocurrent increases sharply until saturation at 10 μA cm^–2^ is achieved after 5 min (Figure 4c). In contrast to the fast rise time, a slower decay time was observed due to the stabilization of photogenerated electrons in the framework. Chronoamperometry measurements under illumination show a stable photocurrent of 10 μA cm^–2^ up to 24 h. To extract the photogenerated electrons, linear sweep voltammetry was performed by applying a voltage sweep from 0.2 V to −0.65 V vs Ag/AgCl at a scan rate of 10 mV s^–1^ (Figure 4d). Upon illumination, a positive current indicates the generation of photoelectrons withdrawn from the material, while a negative current corresponds to electron injection through the FTO into the material or electrolyte due to the applied negative bias. Furthermore, the photocurrent response was also measured at different potentials (swept from 0 V to −0.65 V vs Ag/AgCl) applied to the photoanode (Figure 4e). Upon illumination, photoelectron generation populates the conduction band, marked by a positive extraction current, which is multifold in the presence of a donor, underlining efficient hole extraction. Without a donor, the smaller positive current density diminishes to zero at −0.3 V vs Ag/AgCl, and a negative bias higher than this results in a negative (i.e., injection) current. In the presence of a donor, the electrode is charged efficiently after hole extraction, and the charges are stabilized at low voltages, resulting in higher positive current. Continuous increment in the negative voltage impedes hole extraction, marked by a decreasing positive current, which eventually becomes negative at −0.65 V vs Ag/AgCl. This further highlights the (pseudo)­capacitive charge storage region between −0.3 and −0.65 V vs Ag/AgCl in the presence of a sacrificial electron donor.

**Figure 4:**
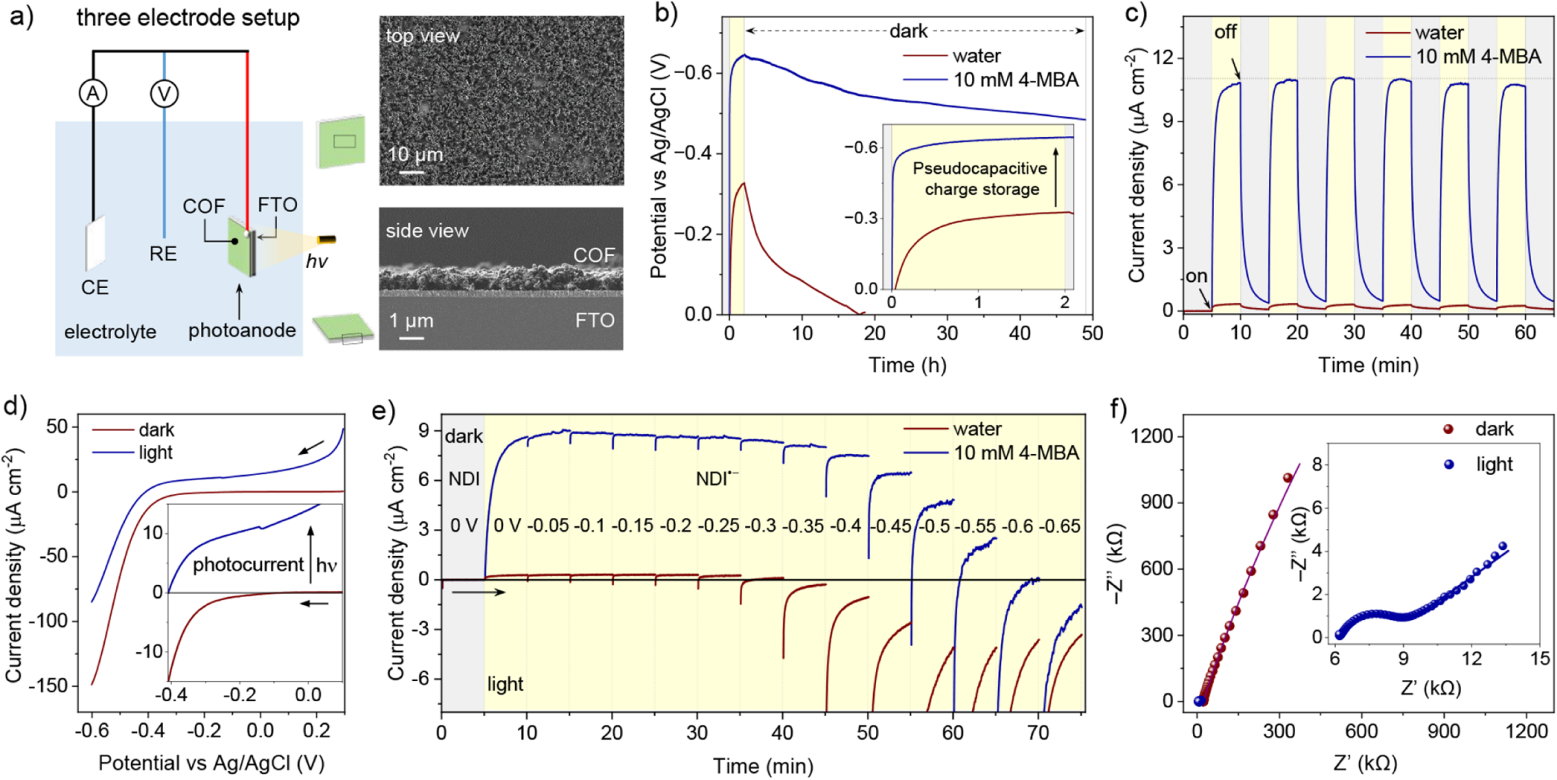
(a) Schematic diagram of the three electrode setup and SEM images showing the top-view and side-view of the COF photoanode. (b) Open circuit potential stability during and after light illumination in the absence and presence of SED 4-MBA, showing the stability of stored electrons in the dark. The inset shows the OCP during photocharging for 2h. (c) Transient photocurrent response in the absence and presence of SED 4-MBA. (d) Linear sweep voltammetry measurement under dark and 1 sun illumination at a scan rate of 10 mV s^–1^. The inset shows the magnified region between 0 and −0.4 V. (e) Chronoamperometry measurements at different potentials under illumination in the absence and presence of SED 4-MBA, which is a measure of electron generation and self-discharge current. (f) Impedance measurements in 10 mM 4-MBA aqueous solution in the ground state (dark) and in the light-induced radical state, showing significantly lower charge transfer resistance in the latter. The inset shows a Nyquist plot of the radical state.

Electrochemical impedance spectroscopy (EIS) was used to probe the effect of light illumination on the charge transfer characteristics of the NDI-COF. A small voltage perturbation over a wide range of frequencies can differentiate physical processes before and after light illumination. To highlight the changes caused by illumination, Bode plots of experimental impedance data are presented as a function of OCP values vs Ag/AgCl at different illumination times (Figure S25). The impedance value at a lower frequency of 0.01 Hz decreases by more than 10-fold after light illumination. The Nyquist plot and the fitted equivalent circuit are shown in Figures 4f and S26. Quantitative analyses using the shown equivalent circuits (EC) suggest that, in the dark, the NDI-COF has a high bulk resistance *R*_C_ of 18.2 MΩ, ascribed to the low conductivity (1.12 × 10^–10^ S cm^–1^) of the material, which is in parallel to a constant phase element (CPE) and a resistance *R*_S_ (22 kΩ) in series, accounting for serial resistance including electrode contact and the electrolyte. After light illumination, an equivalent circuit includes a series resistance *R*_S_, a charge transfer resistance *R*_CT_ in parallel to *C*_CT_, as well as a resistive element *R*_C_ accounting for the materials’ bulk conductivity, with a constant phase element (CPE) in parallel describing a pseudocapacitance *C*_P_. After 1 sun illumination, the *R*_C_ value decreased by 4 orders of magnitude to around 1867 Ω, indicating a substantial increase in conductivity (1.09 × 10^–6^ S cm^–1^) of the system. This apparent increase in conductivity observed in the radical state can be attributed to the combination of both electronic and ionic conductivities of the radical anion in an aqueous environment. Details of the fitted parameters of equivalent circuits in the dark and light are shown in Tables S1 and S2.

### 2.5 COF Solar Battery Performance

Based on these results, we hypothesized that NDI-COF can be utilized as an efficient photoabsorber material, which simultaneously has the built-in ability to store charge and deliver it on demand. To quantify such combined light-harvesting and charge storage/release properties of the NDI-COF solar battery photoanode, we charged the photoanode under illumination and discharged it electrically in the dark (Figure S27). Prior to all experiments, an activation was performed by applying a 0 V bias for 5 min in order to remove any unwanted charges due to stray light. The photoanode was irradiated from the back for a given time, and after switching off the light, it was discharged at a given current until the voltage dropped back to 0 V (Figure 5a). Upon illumination, the OCP value increases instantly to about −0.5 V in 1 min, underlining the photovoltaic effect, and thereafter slowly attains a saturation point of −0.63 V versus Ag/AgCl at 2 h. Figure 5a shows subsequent electrical discharging at a current density of 15 mA g^–1^ for different charging times (1–120 min). The discharge curve resembles the shape of a galvanostatic charge–discharge battery measurement, indicating a faradaic charge storage mechanism to be at play: a plateau-like potential at −0.4 V and a sharp voltage drop to 0 V. Note that the pseudocapacitive response in NDI-COF discussed above involves electron transfer, i.e., redox reactions, consistent with a faradaic process. Discharging kinetics experiments were performed by charging the photoanode with 1 sun for seven different illumination durations (1, 3, 5, 10, 30, 60, and 120 min) under OCP conditions and subsequent galvanostatic discharging in the dark by applying different current densities (15, 50, 100, 200, and 500 mA g^–1^).

**Figure 5:**
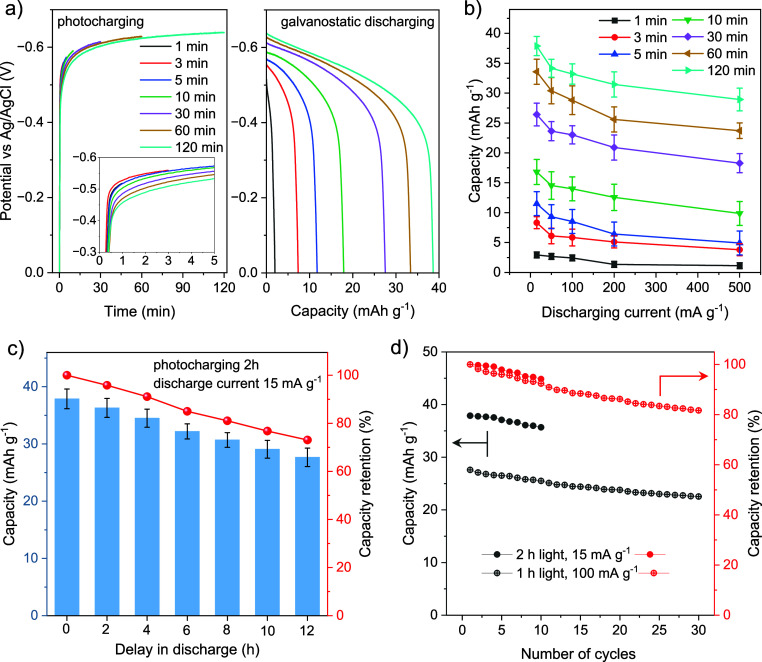
(a) Charging of the solar battery photoanode with different illumination times at 1 sun and under OCP conditions (inset shows a zoomed-in view of short illumination times). Subsequent electric discharge in the dark with a fixed current density of 15 mA g^–1^ (normalized against mass of NDI-COF). (b) Kinetic study of the discharging process. Charging is performed via illumination for different time durations, and subsequent immediate discharging is carried out with different discharging currents (15, 50, 100, 200, and 500 mA g^–1^). (c) Comparison of capacity and capacity retention for delayed discharge at 15 mA g^–1^ after 2 h of photocharging. (d) Evolution of capacity and capacity retention for multiple cycles of photocharging and subsequent immediate discharging.

As seen in Figure 5b, for all illumination times, smaller discharging current densities result in larger capacities, as is often observed in battery materials where charge transport is less limiting. Indeed, less severe resistive losses due to the intrinsically low conductivity and fewer diffusion limitations are invoked for such behavior also in K-PHI solar batteries.^5^ Discharging current density-dependent capacity variation is more pronounced for shorter illumination times than for longer ones. For example, for 10 min of illumination, the increase in capacity from current densities of 500 to 15 mA g^–1^ is 58%, while for 2 h of illumination, it is 33%. The maximum capacity of 38 mAh g^–1^ was achieved after 2 h of photocharging, followed by discharging at a current density of 15 mA g^–1^. In terms of charge storage capacity, NDI-COF surpasses previously reported materials such as K-PHI,^5^ MOFs,^7,8^ and metal oxides^14^ by several folds (Table S3). A further increase in illumination time did not show considerable enhancement in capacity, possibly due to competitive charge loss via faradaic charge transfer, i.e., self-discharge of the material. The thickness of the film varies with the amount of COF loading, affecting the overall capacity, as seen from the optimization experiments (Figure S28). The charge storage capacity of NDI-COF was estimated across multiple batches to demonstrate high reproducibility and minimize estimation error (Figures S29 and S30). At the maximum capacity of 38 mAh g^–1^, the solar battery photoanode can deliver an energy of 15 W h kg^–1^. The respective energy and power output at different illumination times are plotted against different discharging currents in Figure S31. At all illumination times, power output is approximately constant since it is governed by the photovoltage. When only light energy larger than the bandgap of NDI-COF is considered, a maximum solar-to-output efficiency of 0.012% is obtained (Figure S32), while the solar-to-output efficiency is reduced to 0.002% if the entire solar spectrum is considered.

To investigate the longevity/stability of the stored charge by solar charging and its time-delayed extraction, the electrical discharge was delayed by a given amount of time (Figures 5c and S33). After 2 h of illumination, when the OCP reached −0.62 V versus Ag/AgCl, the illumination was stopped, and the dark stability of the OCP was checked before discharging at a current density of 15 mA g^–1^. A stable OCP up to 12 h indicates the efficiency of charge trapping in NDI-COF and its long-term stability. Capacity slowly decreases with an increase in the delayed discharging, but only by 15% for a 6-h delay. Even after 12 h, the photoanode retains 72% of its capacity, reaffirming the robust charge storage in NDI-COF and its on-demand release. Faradaic charge transfer from the partially uncovered FTO substrate to the electrolyte might be the reason for the decay in capacity. This was confirmed by comparing the self-discharge of photoanodes with different areas (0.5 cm × 0.5 cm vs 1 cm × 1 cm) loaded with the same amount of COF. Faster discharge kinetics were observed for the larger substrate, where the uncovered area was comparatively higher.

Next, the cycling performance and stability of the photoanode were investigated over several cycles of photocharging and electric discharging (Figure 5d). Photocharging for 2h and subsequent electric discharging at 15 mA g^–1^ show a capacity retention of 94% after 10 cycles. As these measurements span a longer time, they can also affect the stability of the electrode and lead to a decrease in the electrolyte amount under argon purging. Hence, we performed the cycling test for 30 cycles with 1 h of photocharging and electric discharging at a higher current density of 100 mA g^–1^. Note that, before each cycle, a bias of 0 V was applied for 10 min, as the electrode is not fully discharged when a higher discharge current is applied. Despite a loss of crystallinity (Figure S34), after 30 cycles of photocharging–discharging, a high capacity retention of 82% was achieved, implying the robustness and reversible functionality of the NDI-COF as an electron reservoir system.

### 2.6 Understanding Charge Trapping

From the above discussion, it is clear that above-band-gap illumination of NDI-COF generates electron–hole pairs, and after quenching of holes by a suitable sacrificial electron donor, the electron remains trapped on the material for up to several hours. At this point, we have substantiated the photoaccumulation of electrons in NDI^•–^ by various spectroscopic and photoelectrochemical methods. Similar to the kinetic analysis of the electrochemical charge storage described in Figure 2, the observed long charge storage times suggest that charge trapping is supported by charge-compensating ions from the solution, which screen the negative charge and enable electron accumulation on the NDI-COF backbone. As such, an optoionic coupling between photoinduced electrons and ions from the solution seems to be at play. The role of external ions in the screening of photoelectrons and the charge storage capacity was thus investigated in 1 M aqueous solutions of different alkali metal ions (Li^+^, Na^+^, K^+^, Rb^+^, and Cs^+^) as shown in Figure S35. Despite a decrease in the photopotential by almost 0.1 V (−0.55 V vs Ag/AgCl), no significant impact on the capacity or stability of charge trapping was observed. In all cases, the charge storage capacity varied between 35 and 38 mAh g^–1^ and the retention rate was around 70% after a 12-h delay in discharging. Hence, the presence of additional external ions in the charge storage process does not seem to be obligatory. However, charge trapping can be enhanced by intrinsic components of the electrolyte, including (decomposition products of) the sacrificial electron donor or simply protons generated during electron donor oxidation. This explains why an appropriate concentration of electron donor is essential, as seen from the concentration-dependent study (Figure S36). In contrast, continuously increasing the alkali metal ion (e.g., K^+^) or H^+^ concentration did not alter the charge storage capacity in a 10 mM 4-MBA aqueous solution (Figures S37 and S38).

To probe the mechanism of electron trapping and stabilization, we further conducted UV–vis spectroscopy of NDI-COF in solvents with different dielectric constants (ε) in the presence of various electron donors (4-MBA, triethylamine, triethanolamine). The combination of solvent and electron donor turns out to be highly relevant for the formation of the photoinduced NDI^•–^ radical, which can be monitored via the absorption spectra (Figures S39–S43). In many instances, no photochromic phenomenon was observed after 10 min of irradiation due to either a faster charge recombination process or the unsuitable oxidation potential of the electron donor for successful hole quenching in the solvent of choice (Tables S4 and S5 and Figure S39). In several other cases, despite the formation of the NDI^•–^ radical anion, the retention rate—i.e., stability—of the radicals as a function of time was poor. In most solvents, the signal for the radical anion decreased by more than 30–50% within 10 min. Besides, the addition of external counterions aimed at screening photoelectrons could not increase charge retention. Among all solvents, NDI^•–^ exhibited superior stability in water for more than 48 h for all of the electron donors used, as observed from almost unaltered spectral features (Table S5 and Figure S42). Furthermore, control EPR measurements in acetonitrile show fast regression of the NDI^•–^ radical signal at *g* = 2.0039(1), with complete disappearance within 12 h (Figures S44 and 45), whereas in water, the signal intensity remains unchanged even after 12 h. This points out that long-term charge stabilization does not necessarily require additional external ions, but that the interplay between the solvent (i.e., its dielectric screening ability) and the SED (and its decomposition products) is more critical.

In order to examine the special role of water and shed light on the enhanced stability of NDI^•–^ in this solvent, we performed DFT simulations (see the Theoretical Methods section in Supporting Information for details). The insignificance of added ions (Li^+^, Na^+^, K^+^, Rb^+^, and Cs^+^) for long-term storage of the NDI^•–^ radical anion first led us to hypothesize that the stability, instead of being due to metal cations, is due to a proton-mediated mechanism. Both water and alcohol (provided by or formed upon decomposition of the electron donor) are sources of protons, which may easily find the NDI^•–^ moieties in the COF and serve as charge-compensating species. Natural sites for protonation are the NDI oxygens. To compare the capability of stabilizing excess electrons, we first calculated the energy difference between the electron affinity of NDI and that of protonated NDIH^+^ in the gas phase. Figure 6a,b shows that the latter would stabilize electrons stronger by >4 eV, at least for a single molecule. We also find that excess electrons are stabilized by the NDI stack in the COF as compared to an isolated NDI due to the electrostatic quadrupolar characteristics of neighboring NDIs.^47^ This was further verified by comparing the charge storage capacity of NDI-COF with that of an analogous amorphous NDI polymer (Figures S46–S49). The lower capacity and poor charge retention ability of the NDI polymer reinforce the role of a highly porous and well-accessible crystalline framework for long-term charge stabilization.

**Figure 6:**
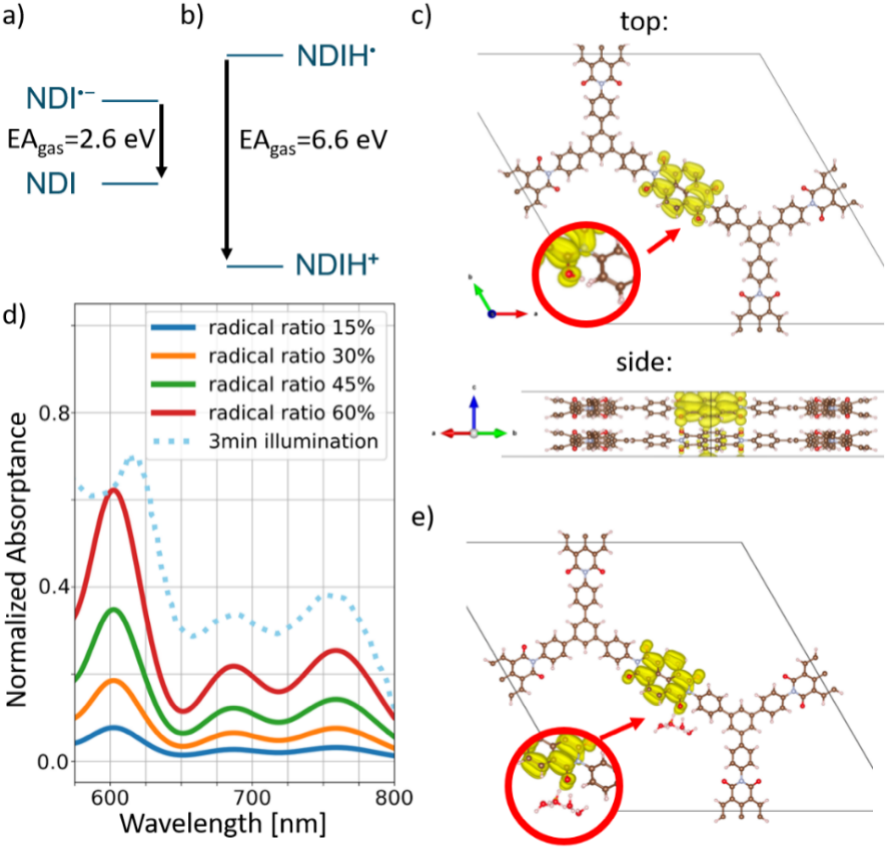
(a), (b) Term schemes for different charging states. (c) Relaxed structure of the COF with an additional hydrogen atom forming a hydroxyl group at the former carbonyl carbon (NDIH^•^). Excess electron is localized in the former LUMO of the NDI fragment. (d) Calculated low-energy absorption spectrum of NDI radicals as a function of radical concentration (solid lines) and comparison to experiments (dotted line). (e) Relaxed structure with additional hydrogen and water clusters ([H_2_O]_4_) leads to solvation of H^+^ and favors more delocalization of the excess electron within the NDI stack.

Third, the involvement of protonated COF was examined in detail. Note that we compared different protonation sites across the NDI and other fragments in simulations with one additional proton in a doubled unit cell in the stacking direction, showing a clearly preferred position of the proton at the oxygen atom, as shown in Figure 6c. This configuration is called NDIH^•^ in the following and was clearly more stable than intercalated protons by 0.7–2.7 eV (depending on the intercalation position). Further analysis of the NDIH^•^ configuration showed charge transfer to the former NDI LUMO, as illustrated in Figure 6c. Here, the localization of the excess electron charge is restricted to the NDIH^•^; i.e., in the presence of the protonated oxygen, the excess electron cannot delocalize through the π stack of NDI moieties. The absorption spectra of NDI^•–^ and NDIH^•^ chromophores are investigated by time-dependent DFT (TDDFT, see Supporting Information) to find more evidence of the protonation state of the radical species, but the spectra of NDI^•–^ and NDIH^•^ fragments were not sufficiently different to result in a clear preference for either species, partly because of the strong vibronic replica features. In fact, the simulations show that the mixture of both could closely resemble the COF radical absorption, with a good spectral match for relative concentrations of both radicals of about *r* = 85:15 (Figure 6d). Here, we plot the computed low-energy absorption spectrum with varying relative concentrations of the charge-neutral NDI molecule and both radical components, NDI^•–^ and NDIH^•^ (fixing the value of *r*). We observe a gradual increase in the absorption of the radical species, in agreement with the behavior of the low-energy absorptance in Figure 3a with illumination time.

As a next step, we added water molecules to the simulations and placed them in close vicinity of the NDIH^•^. The result of structural relaxation is shown in Figure 6e, where four water molecules were initially grouped around the OH group, and the proton was abstracted from NDIH^•^ and dissolved in the water cluster. Still, the electron charge resides on the COF NDI, in agreement with all experimental data. From this, we conclude that protons may not stabilize the NDI radicals in H_2_O solvent, similar to our conclusions regarding metal cations, as the charge can equally be compensated on a more global scale, and a localized charge compensation is not necessary. On the other hand, water itself leads to the screening and stabilization of NDI^•–^ through its polarizability. When comparing water as a solvent to others like acetonitrile, where the polarizability, dipole interactions, hydrogen bonding, and ability to dissolve protons are less pronounced, the screening of NDI^•–^ is substantially reduced, as quantified by the lower dielectric constants of these solvents compared to water. It is thus reasonable to conclude that the NDI^•–^ radical anion is most stabilized in water due to its high polarizability and dielectric screening ability, resulting in a maximized lifetime of the radical anion. A possible quencher is dissolved oxygen in water, which has a high electron affinity. In this context, we emphasize that the lowering of the NDI^•–^ energy by both mechanisms—dielectric screening due to H_2_O and a local electrostatic attraction of the stacked NDI units in the COF—increases the reaction barrier for forming superoxide (O_2_^•–^), thereby extending the radical’s lifetime. Additionally, charge delocalization may significantly contribute to reduced reactivity and a prolonged lifetime.

## 3 Conclusion

To summarize, we have demonstrated the combination of solar energy harvesting, conversion, and storage in NDI-COF as an earth-abundant material. Light-induced electrons remain trapped on the framework backbone for several hours and can be extracted on demand to generate electrical energy. This dual functionality of light harvesting and charge storage in the NDI-COF has been exploited to demonstrate an aqueous solar battery photoanode for decentralized electrochemical energy storage. By combining spectroscopic and photoelectrochemical characterization with insights from theory, we pinpoint the crucial role of water in stabilizing the NDI radical anion by dielectric screening, along with efficient charge delocalization across the COF backbone. In contrast, neither the type nor concentration of extra cations in the electrolyte has a pronounced influence on charge trapping. Photocharging of NDI-COF results in a stable OCP for more than 48 h, which, in combination with an optimum charge storage capacity of 38 mAh g^–1^ sets NDI-COF apart from other reported photoanode materials, both in terms of capacity and cycling stability. The molecular-level tunability of COFs and their fast and reversible pseudocapacitive charge storage, enabled by their large, accessible porosity, thus opens new perspectives for the rational design of high-performance, sustainable solar battery electrodes based on reticular materials.

## Supplementary Material



## Notes

The supporting Information contains detailed experimental and computational methods, along with corresponding supporting Figures S1–S49 and supporting Tables S1–S5 mentioned in the main text (PDF)

## References

[1] Zhang L., Wang Y. (2023). Decoupled Artificial Photosynthesis. Angew.
Chem., Int. Ed..

[2] Rogolino A., Savateev O. (2023). Photochargeable Semiconductors: In “Dark Photocatalysis”
and Beyond. Adv. Funct. Mater..

[3] Lv J., Xie J., Mohamed A. G. A., Zhang X., Wang Y. (2022). Photoelectrochemical
energy storage materials: Design principles and functional devices
towards direct solar to electrochemical energy storage. Chem. Soc. Rev..

[4] Lau V. W., Klose D., Kasap H., Podjaski F., Pignie M. C., Reisner E., Jeschke G., Lotsch B. V. (2017). Dark Photocatalysis:
Storage of Solar Energy in Carbon Nitride for Time-Delayed Hydrogen
Generation. Angew. Chem., Int. Ed..

[5] Podjaski F., Kroger J., Lotsch B. V. (2018). Toward
an Aqueous Solar Battery:
Direct Electrochemical Storage of Solar Energy in Carbon Nitrides. Adv. Mater..

[6] Kröger J., Jiménez-Solano A., Savasci G., Rovó P., Moudrakovski I., Küster K., Schlomberg H., Vignolo-González H. A., Duppel V., Grunenberg L. (2020). Interfacial Engineering
for Improved Photocatalysis in a Charge Storing
2D Carbon Nitride: Melamine Functionalized Poly­(heptazine imide). Adv. Energy Mater..

[7] Stanley P. M., Sixt F., Warnan J. (2023). Decoupled Solar Energy Storage and
Dark Photocatalysis in a 3D Metal–Organic Framework. Adv. Mater..

[8] Wu S., Stanley P. M., Deger S. N., Hussain M. Z., Jentys A., Warnan J. (2024). Photochargeable Mn-Based
Metal–Organic Framework
and Decoupled Photocatalysis. Angew. Chem.,
Int. Ed..

[9] Ahmad S., George C., Beesley D. J., Baumberg J. J., De Volder M. (2018). Photo-Rechargeable
Organo-Halide Perovskite Batteries. Nano Lett..

[10] Amthor S., Knoll S., Heiland M., Zedler L., Li C., Nauroozi D., Tobaschus W., Mengele A. K., Anjass M., Schubert U. S. (2022). A photosensitizer-polyoxometalate dyad that enables
the decoupling of light and dark reactions for delayed on-demand solar
hydrogen production. Nat. Chem..

[11] Boruah B. D., Wen B., De Volder M. (2021). Light Rechargeable
Lithium-Ion Batteries Using V(2)­O(5)
Cathodes. Nano Lett..

[12] Boruah B. D., Mathieson A., Wen B., Feldmann S., Dose W. M., De Volder M. (2020). Photo-rechargeable
zinc-ion batteries. Energy Environ. Sci..

[13] Lou S. N., Sharma N., Goonetilleke D., Saputera W. H., Leoni T. M., Brockbank P., Lim S., Wang D. W., Scott J., Amal R. (2017). An Operando Mechanistic
Evaluation of a Solar-Rechargeable Sodium-Ion
Intercalation Battery. Adv. Energy Mater..

[14] Wang Y., Chan Y. T., Oshima T., Duppel V., Bette S., Kuster K., Gouder A., Scheurer C., Lotsch B. V. (2024). Decoupling
of Light and Dark Reactions in a 2D Niobium Tungstate for Light-Induced
Charge Storage and On-Demand Hydrogen Evolution. J. Am. Chem. Soc..

[15] Ruan Q., Xi X., Yan B., Kong L., Jiang C., Tang J., Sun Z. (2023). Stored photoelectrons
in a faradaic junction for decoupled solar
hydrogen production in the dark. Chem.

[16] Gouder A., Podjaski F., Jimenez-Solano A., Kroger J., Wang Y., Lotsch B. V. (2023). An integrated solar battery based on a charge storing
2D carbon nitride. Energy Environ. Sci..

[17] Podjaski F., Lotsch B. V. (2020). Optoelectronics
Meets Optoionics: Light Storing Carbon
Nitrides and Beyond. Adv. Energy Mater..

[18] Keller N., Bein T. (2021). Optoelectronic processes in covalent
organic frameworks. Chem. Soc. Rev..

[19] Wang D.-G., Qiu T., Guo W., Liang Z., Tabassum H., Xia D., Zou R. (2021). Covalent organic
framework-based materials for energy applications. Energy Environ. Sci..

[20] Tan K. T., Ghosh S., Wang Z., Wen F., Rodríguez-San-Miguel D., Feng J., Huang N., Wang W., Zamora F., Feng X. (2023). Covalent organic frameworks. Nat. Rev. Methods
Primers.

[21] Lv J., Tan Y. X., Xie J., Yang R., Yu M., Sun S., Li M. D., Yuan D., Wang Y. (2018). Direct Solar-to-Electrochemical
Energy Storage in a Functionalized Covalent Organic Framework. Angew. Chem., Int. Ed..

[22] Zhou E., Zhang X., Zhu L., Yuan D., Wang Y. (2023). A Solar Responsive
Battery Based on Charge Separation and Redox Coupled Covalent Organic
Framework. Adv. Funct. Mater..

[23] Wang W., Zhang X., Lin J., Zhu L., Zhou E., Feng Y., Yuan D., Wang Y. (2022). A Photoresponsive
Battery
Based on a Redox-Coupled Covalent-Organic-Framework Hybrid Photoelectrochemical
Cathode. Angew. Chem., Int. Ed..

[24] Bhosale S. V., Al Kobaisi M., Jadhav R. W., Morajkar P. P., Jones L. A., George S. (2021). Naphthalene diimides: Perspectives
and promise. Chem. Soc. Rev..

[25] Kobaisi M. A., Bhosale S. V., Latham K., Raynor A. M., Bhosale S. V. (2016). Functional
Naphthalene Diimides: Synthesis, Properties, and Applications. Chem. Rev..

[26] Bhosale S. V., Jani C. H., Langford S. J. (2008). Chemistry of naphthalene
diimides. Chem. Soc. Rev..

[27] Wiberg C., Busch M., Evenäs L., Ahlberg E. (2021). The electrochemical
response of core-functionalized naphthalene Diimides (NDI) –
a combined computational and experimental investigation. Electrochim. Acta.

[28] Shi Y., Tang H., Jiang S., Kayser L. V., Li M., Liu F., Ji F., Lipomi D. J., Ong S. P., Chen Z. (2018). Understanding
the Electrochemical Properties of Naphthalene Diimide: Implication
for Stable and High-Rate Lithium-Ion Battery Electrodes. Chem. Mater..

[29] Royuela S., Martínez-Periñán E., Arrieta M. P., Martínez J. I., Ramos M. M., Zamora F., Lorenzo E., Segura J. L. (2020). Oxygen
reduction using a metal-free naphthalene diimide-based covalent organic
framework electrocatalyst. Chem. Commun..

[30] Yu M., Chandrasekhar N., Raghupathy R. K. M., Ly K. H., Zhang H., Dmitrieva E., Liang C., Lu X., Kuhne T. D., Mirhosseini H. (2020). A High-Rate
Two-Dimensional Polyarylimide Covalent
Organic Framework Anode for Aqueous Zn-Ion Energy Storage Devices. J. Am. Chem. Soc..

[31] Van
Gele S., Bette S., Lotsch B. V. (2025). The Devil Is in the Details: Pitfalls
and Ambiguities in the Analysis of X-ray Powder Diffraction Data of
2D Covalent Organic Frameworks. JACS Au.

[32] van
der Jagt R., Vasileiadis A., Veldhuizen H., Shao P., Feng X., Ganapathy S., Habisreutinger N. C., van der Veen M. A., Wang C., Wagemaker M. (2021). Synthesis
and Structure-Property Relationships of Polyimide Covalent Organic
Frameworks for Carbon Dioxide Capture and (Aqueous) Sodium-Ion Batteries. Chem. Mater..

[33] Castner A. T., Su H., Svensson Grape E., Inge A. K., Johnson B. A., Ahlquist M. S. G., Ott S. (2022). Microscopic
Insights into Cation-Coupled
Electron Hopping Transport in a Metal-Organic Framework. J. Am. Chem. Soc..

[34] Li J., Kumar A., Johnson B. A., Ott S. (2023). Experimental manifestation
of redox-conductivity in metal-organic frameworks and its implication
for semiconductor/insulator switching. Nat.
Commun..

[35] Augustyn V., Come J., Lowe M. A., Kim J. W., Taberna P. L., Tolbert S. H., Abruna H. D., Simon P., Dunn B. (2013). High-rate
electrochemical energy storage through Li+ intercalation pseudocapacitance. Nat. Mater..

[36] Lee J., Lim H., Park J., Kim M.- S., Jung J.- W., Kim J., Kim I.- D. (2023). Fluorine-Rich Covalent Organic Framework to Boost Electrochemical
Kinetics and Storages of K+ Ions for Potassium-Ion Battery. Adv. Energy Mater..

[37] Qiu M., Sun P., Shen L., Wang K., Song S., Yu X., Tan S., Zhao C., Mai W. (2016). WO 3 nanoflowers with excellent pseudo-capacitive
performance and the capacitance contribution analysis. J. Mater. Chem. A.

[38] Jin S., Ding X., Feng X., Supur M., Furukawa K., Takahashi S., Addicoat M., El-Khouly M. E., Nakamura T., Irle S. (2013). Charge dynamics
in a donor-acceptor
covalent organic framework with periodically ordered bicontinuous
heterojunctions. Angew. Chem., Int. Ed..

[39] Levine A. M., Biswas S., Braunschweig A. B. (2019). Photoactive organic material discovery
with combinatorial supramolecular assembly. Nanoscale Adv..

[40] Li J., Kumar A., Ott S. (2024). Diffusional
Electron Transport Coupled
to Thermodynamically Driven Electron Transfers in Redox-Conductive
Multivariate Metal-Organic Frameworks. J. Am.
Chem. Soc..

[41] AlKaabi K., Wade C. R., Dincǎ M. (2016). Transparent-to-Dark Electrochromic
Behavior in Naphthalene-Diimide-Based Mesoporous MOF-74 Analogs. Chem.

[42] Leong C. F., Chan B., Faust T. B., D’Alessandro D. M. (2014). Controlling
charge separation in a novel donor–acceptor metal–organic
framework via redox modulation. Chem. Sci..

[43] Maniam S., Higginbotham H. F., Bell T. D. M., Langford S. J. (2019). Harnessing Brightness
in Naphthalene Diimides. Chem.

[44] Kim B., Lee J., Chen Y.-P., Wu X.-Q., Kang J., Jeong H., Bae S.-E., Li J.-R., Sung J., Park J. (2022). π-Stacks
of radical-anionic naphthalenediimides in a metal-organic framework. Sci. Adv..

[45] Kumar Y., Kumar S., Bansal D., Mukhopadhyay P. (2019). Synthesis
and Isolation of a Stable Perylenediimide Radical Anion and Its Exceptionally
Electron-Deficient Precursor. Org. Lett..

[46] Zhang A., Jiang W., Wang Z. (2020). Fulvalene-Embedded Perylene Diimide
and Its Stable Radical Anion. Angew. Chem.,
Int. Ed..

[47] Bittner E.-A., Merkel K., Ortmann F. (2024). Engineering the electrostatic potential
in a COF’s pore by selecting quadrupolar building blocks and
linkages. Npj 2D Mater. Appl..

