# Supplementary material for: Insights into Decoupled Solar Energy Conversion and Charge Storage in a 2D Covalent Organic Framework for Solar Battery Function
Source: J Am Chem Soc. 2025 Apr 28;147(22):18492–503. doi: 10.1021/jacs.4c17642 (PMC12147126; doi:10.1021/jacs.4c17642)
Supplement: Supplementary file 1 [file ja4c17642_si_001.pdf]

Supporting Information

Insights into Decoupled Solar Energy Conversion and Charge Storage in a 2D Covalent Organic Framework for Solar Battery Function

Bibhuti Bhusan Rath,[1,*] Laura Fuchs,[2] Friedrich Stemmler,[3] Andrés Rodríguez-Camargo,[1,4] Yang Wang,[1] Maximilian F. X. Dorfner,[2] Johann Olbrich,[2] Joris van Slageren,[3] Frank Ortmann,[2,*] and Bettina V. Lotsch[1,5,6*]

[1] Nanochemistry Department, Max Planck Institute for Solid State Research, Heisenbergstraße 1, 70569 Stuttgart, Germany

[2] Department of Chemistry, TUM School of Natural Sciences, Technische Universität München, Garching, 85748 München, Germany

[3] Institute of Physical Chemistry, University of Stuttgart, Pfaffenwaldring 55, 70569 Stuttgart, Germany

[4] Department of Chemistry, University of Stuttgart, Pfaffenwaldring 55, 70569 Stuttgart, Germany

[5] Department of Chemistry, Ludwig-Maximilians-Universität (LMU), Butenandtstraße 5-13, 81377 Munich, Germany

[6] E-Conversion and Center for Nanoscience, Lichtenbergstraße 4a, Garching, 85748 Munich, Germany

* Corresponding authors. E-mails: bb.rath@fkf.mpg.de, frank.ortmann@tum.de, b.lotsch@fkf.mpg.de

1. Materials, Instrumentation and Simulations

Materials

Commercially available reagents and solvents were purchased from Sigma-Aldrich, TCI chemicals, Avra chemicals, and used without further purification.

General instrumentations and methods

Supercritical CO_2 ($scCO_2$). After an overnight Soxhlet treatment, Leica EM CPD300 critical point dryer was used for $scCO_2$ rinsing and drying of NDI-COF. Initially, the sample chamber was half-filled with methanol at 13 °C and the liquid was stirred for 15 min post CO_2 infusion. After around 20 exchange cycles, CO_2 was removed at 40 °C.

Infrared (IR) absorbance spectroscopy. IR absorbance spectra were recorded using PerkinElmer Spectrum Two spectrometer in attenuated total reflection (ATR) geometry equipped with a diamond crystal. Ten measurements were averaged with a resolution of 4 cm^{-1}. Spectral post processing was performed by correcting the ATR effect (contact parameter: 0), subtracting the base line and normalizing νC=C peaks at 1600 cm^{-1}.

Powder X-ray diffraction (PXRD). PXRD patterns were collected using a Stoe Stadi P diffractometer with a Cu Kα_1 source monochromatized with Ge(111) in a Debye-Scherrer geometry at room temperature (RT). Powder samples sealed in ø1.0 mm glass No. 14 capillaries were measured with spinning.

Solid-state nuclear magnetic resonance (SSNMR) spectroscopy. ^{13}C cross-polarization (CP) magic-angle spinning (MAS) SSNMR spectra were recorded using a Bruker Avance III 400 MHz spectrometer. The measurements were done at a rotation frequency of 20000 Hz, and a contact time (p15) of 3000 µs at a temperature of 293 K. The chemical shifts (δ) were referenced relative to α-glycine (176.5 ppm).

Scanning electron microscopy (SEM). SEM images were recorded on a Zeiss Merlin microscope under the electron high tension voltage of 1.5 kV. The samples were cast on indium-doped tin oxide (ITO) substrates.

Transmission electron microscopy (TEM). TEM images were recorded on a Philips CM 30 ST microscope with a LaB6 cathode and TVIPS TemCam-F216 CMOS camera operated under 300 kV. The samples were gently ground and contacted with a holey carbon/copper grid.

Gas sorption measurements. Argon sorption measurements were conducted at 77 K using a Quantachrome Instruments Autosorb iQ 3. The pore size distribution (PSD) was determined by employing the Ar adsorption data at 77 K and applying the QSDFT model (cylindrical pores, adsorption branch) within ASiQwin software version 3.01. Prior to measurement, the NDI COF sample was subjected to activation under high vacuum conditions at 120 °C for a duration of 12 hours. For the determination of the BET surface area, a specific pressure range (P/P_0 = 0.02–0.2) was selected.

Ultraviolet–visible (UV–vis) spectroscopy. UV–vis absorptance spectra were recorded on a Cary 60 UV–Vis spectrophotometer with a 1 cm cuvette at RT.

UV-vis spectroelectrochemistry was conducted by combining Metrohm Autolab potentiostat (PGSTAT302) with Nova 2.1.4 software electrochemistry setup with the above spectrophotometer. A cuvette was employed as the electrochemical cell, with Pt wire as pseudoreference and all other conditions remained the same as above.

Electron paramagnetic resonance spectroscopy. EPR measurements were conducted on a Bruker EMX spectrometer at room temperature with X-band frequencies. The spectrum fitting was conducted with the EasySpin (Version 6.0.0-dev.53) software package[1] for Matlab (Version R2022a) using a spin Hamiltonian with isotropic g tensors ($H = \mu_B \boldsymbol{SgB}_0$) and a Voigtian line broadening.

The electrochemical cell for the in situ EPR spectroelectrochemistry was a basic three electrode setup of Pt/Pt/AgCl fitted into an EPR tube under inert atmosphere. Electrochemical potentials were applied using a Keithley 2450 SMU and a home-written program for cyclovoltammetry experiments.

Electrochemical and Photo-electrochemical methods

Electrode preparation. NDI-COF ink was prepared by vigorously stirring 5 mg of pristine COF in a 9: 3: 1 ratio of DMF: EtOH: Nafion (total volume 10 mL) for 2 h, yielding a dispersion of 0.5 mg/ml. SIGMA ALDRICH FTO slides (surface resistivity: 7 Ohm/sq) were cut to dimension of 7×6 mm or 11×10 mm, and were thoroughly washed with water and isopropanol followed by O_2 plasma cleaning for 10 minutes. An equivalent of COF ink usually 10 µg was drop cast on the FTO substrates and dried on a hot plate at 50°C for 30 minutes. The COF film was scratched a little at the corner of the substrate and silver paste was used to make contact with isolated copper wire. The contact was properly sealed with epoxy (3 M Scotch-Weld DP410), leaving an exposed electrode area of approximately 5x5 mm² or 10x10 mm².

(Photo)electrochemical measurements. All the (photo)electrochemical measurements were performed in a custom made closed glass reactor equipped with a quartz window for illumination. A conventional three electrode setup with Ag/AgCl (saturated KCl, RE-1CP) as reference electrode, platinum wire as counter electrode was used. Prior to every measurement, the electrolyte was purged with >99% pure Ar through a porous glass frit to remove oxygen and ensure O_2 free environment. Water, 10 mM 4–MBA, or 1 M MCl (M = Li, Na, K, Rb, Cs) aqueous solution was used as electrolytes for different (photo)electrochemical measurements.

Electrochemical measurements were recorded and analyzed using a multichannel potentiostat (Autolab M204, Metrohm) and the NOVA software. Simulated sunlight was provided by a SCIENCETECH LightLine A4 solar simulator (class AAA) fitting the ASTM standard G138 (AM 1.5G). The intensity of the illumination was measured by a calibrated THORLABS S310C thermal power meter and further confirmed by a calibrated OCEAN OPTICS USB4000 spectrometer. The intensity was set to $P_{nominal} \approx 100$ mW cm^{-2} for all the irradiation experiments.

Cyclic voltammetry measurements were performed in a potential window of 0 ~ −0.9 V vs. Ag/AgCl. Electrochemical impedance measurements were performed in a frequency range of 10 kHz ~ 0.01Hz under 1 sun illumination for different times.

Theoretical Methods

DFT Ground State Geometry of Molecular Simulations

To find a stable equilibrium configuration, we optimize the geometries of the $NDI^{\bullet-}$ and $NDIH^{\bullet}$ molecules in the gas-phase. Hybrid density functional theory in the form of the HSE06 functional[2] has been employed. For the computations, we use the CP2K software package[3] and Goedecker-Teter-Hutter pseudopotentials[4-6] in combination with the triple-zeta double-valence polarization (TZV2P) basis set from the HF basis set, along with a plane-wave cutoff of 500 Ha.

TDDFT: Singlet Excited States

At the optimized geometries we compute the electronic singlet excited states $\{|S_m\rangle\}$ and their energies $\mathcal{E}_m$ by means of linear-response time-dependent density functional theory (TDDFT)[7-10] in the Tamm-Dancoff approximation[11] as implemented in CP2K[3]. For consistency with the geometry optimization we use the same basis set (TZV2P) and HSE06 as the TDDFT kernel. The resulting energies at HSE06 are too high to align with the experimental onsets, as commonly observed. This could be a result of the known overestimation of excited state energies by TDDFT[12], when employing the HSE06 kernel, the presence of the solvent in the experiment that is not present in the gas phase calculations, or the impact of the COF structure in which the NDI is embedded. To correct for this, we apply an empirical rigid shift of -0.285 eV to all the excited state energies.

Molecular Vibrations and Interaction with Electronic Structure

Normal Modes – We compute the normal modes and corresponding energies $\hbar\omega_\lambda$ in the $NDI^{\bullet-}$ ground state by displacing the equilibrium structure by 0.05 a_0 (Bohr radius) along each Cartesian direction, and numerically diagonalizing the mass-weighted Hessian, which is computed using a finite difference approximation to the forces. We take explicitly those modes λ into account, which fulfill $\hbar\omega_\lambda > 0.1$ eV, as for these the static polaron limit in the context of absorption experiments is most appropriate. We assume, that the $NDIH^{\bullet}$ molecule has identical modes.

Linear Coupling Constants – To obtain the exciton-phonon coupling constants for these modes we deflect the equilibrium geometry along the normal mode coordinates X_λ and compute the coupling constants, defined by

$$g_{\lambda m} = \frac{\partial_{X_\lambda}\mathcal{E}_m}{\sqrt{2\hbar\omega_\lambda^3}} \qquad (1)$$

from a finite difference approximation of the TDDFT energies at the displaced geometries. Computationally we use identical settings as above. Non-adiabatic coupling constants are not considered in this work. Similarly to the normal modes, we assume that the coupling constants of the $NDIH^{\bullet}$ are identical to the one of the charged $NDI^{\bullet-}$.

Absorption Spectrum – We compute the absorption spectrum $\bar{\kappa}(\omega)$ by spatial average of the imaginary part of the susceptibility χ'' [13]

$$\bar{\kappa}(\omega) \propto \frac{1}{3}\left[\chi''_{xx}(\omega) + \chi''_{yy}(\omega) + \chi''_{zz}(\omega)\right] \quad (2)$$

Here $\chi''_{ij}(\omega)$ is given by

$$\chi''_{ij}(\omega) = \sum_{\alpha N} \langle 0_N|\hat{\boldsymbol{d}}_i|\alpha_N\rangle\langle\alpha_N|\hat{\boldsymbol{d}}_j|0_N\rangle \delta(\hbar\omega - E_\alpha^N + E_0^N) \quad (3)$$

where $\hat{\boldsymbol{d}}_i$ is the ith component of the transition dipole operator, $|\alpha_N\rangle$ are the exact N-electron eigenstates with energy E_α^N. We use the transition dipole moments from the TD-TDFT simulations.

To account for the vibronic interaction, we use a parameterized linear vibronic coupling model[14-16], with zero non-adiabatic couplings

$$\sum_{m>0} \mathcal{E}_m |S_m\rangle\langle S_m| + \sum_{m,\lambda} \hbar\omega_\lambda g_{\lambda m}\left[\hat{b}_\lambda^\dagger + \hat{b}_\lambda\right]|S_m\rangle\langle S_m| + \sum_\lambda \hbar\omega_\lambda \hat{b}_\lambda^\dagger \hat{b}_\lambda \quad (4)$$

and obtain the exact vibronic eigenstates $|\alpha_N\rangle$ that enter Eq. (2), by a polaron transformation.

For the spectrum we finally take into account up to 2-phonon-excitations and broaden the delta-distributions in Eq. (3) by a Gaussian broadening of width 0.06 eV.

DFT Simulations of COFs

The initial model of the NDI-COF was constructed using experimental powder x-ray diffraction data. The initial structure was relaxed within DFT as implemented in the VASP[17] code using the PBE exchange-correlation functional[18], PAW pseudopotentials[17] and periodic boundary conditions in all directions. The relaxation was performed by first relaxing the ion positions, then the cell volume and finally again the ion positions. The energy cut-off for the relaxation of the ion position was 400 eV while the energy cut-off for the volume of the unit cell was set to 520 eV. The energy convergence value for the self-consistency cycle was 10^{-6} eV. A 1x1x6 k-point grid was used. Van-der-Waals dispersion was included using Grimme's D3 version along with a Becke-Johnson damping function.[19] The resulting structure is in good agreement with experimental data. For the simulations with additional hydrogen atoms and water clusters we extended the simple unit cell to a supercell of doubled size in stacking direction, reduced the kpoint grid to 1x1x3, and performed spin-polarized calculations.

DFT Band Gap Correction to Hybrid Level

Since the PBE functional underestimates the fundamental band gaps of semiconductors systematically, we use a correction scheme from PBE to hybrid DFT at HSE06 level. Three single-point calculations in the Brillouin zone with both functionals were performed along the $\Gamma \rightarrow$ A direction. The obtained energy shift (scissors shift) was applied to all conduction bands accordingly.

Simulation of IR absorption spectra

We investigated theoretically the peak shift of the C−O stretch mode from $1672\ cm^{-1}$ to $1624\ cm^{-1}$ [cf. Fig. 3 (f) of the main manuscript] in the ATR-IR absorption spectrum by computing the IR absorption spectra of the neutral NDI molecule and the radical anion $NDI^{\bullet-}$. To access this quantity, we restricted ourselves to the gas phase and used the vibrational normal mode coordinates X^λ and their energies $\hbar\omega_\lambda$ of NDI and $NDI^{\bullet-}$ as described above.

Molecular Vibrations and Interaction with Electronic Structure.

We further computed the IR dipole matrix element $d_{S,i}^{(\lambda)}$ for the two species $S =$ NDI and NDI$^{•-}$. Together, these quantities enter the IR absorption spectrum, via

$$\alpha(E) \sim \sum_{\lambda,i,S} C_S \left| d_{S,i}^{(\lambda)} \right|^2 \delta(E - \hbar\omega_\lambda),$$

where C_S denotes the concentration of the absorbing species in the mixture of both species. These matrix elements are given by

$$d_{S,i}^{(\lambda)} = e\sqrt{\frac{\hbar}{\omega_\lambda}}\frac{\partial d_{S,i}}{\partial X^\lambda},$$

where e is the elementary charge, $\hbar$ is the reduced Planck's constant, ω_λ is the frequency of the considered mode λ, and $d_{S,i}$ is the ith cartesian component of the total ground state dipole moment of the corresponding species. Furthermore, $\frac{\partial}{\partial X^\lambda}$ denotes the derivative along the normal mode coordinate. To compute $d_{S,i}^{(\lambda)}$, we employ a central difference approach to the derivative along the normal mode coordinate. Here, the ground state geometry is displaced by $\pm\delta = 0.075 a_0$ along the (normalized) mode patterns (defined by the vector e_λ, including all atoms). The total dipole moment is computed as $d_{S,i}(R_0 \pm \delta e_\lambda)$ as implemented in CP2K. Here we employ, consistent with the previous calculations, the HSE06 functional and the TZV2P basis set. Then the derivative is computed as

$$\frac{\partial d_{S,i}}{\partial X^\lambda} \approx \frac{d_{S,i}(R_0 + \delta e_\lambda) - d_{S,i}(R_0 - \delta e_\lambda)}{2\delta\sqrt{M_\lambda}},$$

where $\sqrt{M_\lambda}$ is the reduced mass of the mode. The spectra are broadened by a Gaussian of width 3 meV. To compute the IR absorption spectra of the NDI–NDI$^{•-}$ mixture present in the experiment, we weight the individual (single species) spectra by the same concentrations of radical species used to compute the low-energy UV-vis absorption [cf. Fig. 6 (d)]. The resulting theoretical spectra are compared with the experimental ATR-IR spectra in **Figure S23**. We find that the main absorption features of NDI and NDI$^{•-}$ appearing in the theoretical spectrum are blue-shifted relative to the experiment and show a slightly stronger energetic separation. However, considering that the calculations are based on gas-phase molecules, while in the actual material they are embedded in the COF structure and surrounded by solvent, we find the agreement to be satisfactory. That is, the gradual peak shift with increasing NDI$^{•-}$ concentration in the ATR-IR spectrum is reproduced. Hence, this peak shift can be associated with a red shift of the C−O normal mode energies upon charging NDI, which is accompanied by an approximate doubling of the matrix element $d_{S,i}^{(\lambda)}$ (dipole strength). Thus, this corroborates that the observed peak shift is not indicating stronger structural changes, but results from the higher density of NDI$^{•-}$ species during illumination.

2. Synthesis and Characterizations

Synthesis of NDI-COF: NDI COF was synthesized following a literature procedure with slight modifications.[20] In a 8 mL Schlenk tube, 1,4,5,8-naphthalenetetracarboxylic dianhydride (NTCDA, 40.2 mg, 0.15 mmol) and 1,3,5-tris(4-aminophenyl)benzene (TAPB, 35.1 mg, 0.1 mmol) were suspended in a mixture of N-methyl-2-pyrrolidone (0.75 mL), and mesitylene (0.15 mL). The mixture was sonicated for 5 minutes in order to obtain a homogenous dispersion and isoquinoline (0.05 mL) was added, followed by sonication for 2 minutes. The mixture was frozen in a liquid N_2 bath, the inner atmosphere was evacuated at < 0.01 mbar and three cycles of freeze–pump–thaw was performed. The mixture was sonicated for 5 minutes, and heated in aluminium heating block for 4 days at 120 °C. After cooling to room temperature, the precipitate was collected by filtration and washed with hot N, N-dimethylformamide, ethanol and tetrahydrofuran, while keeping the solids wet. An overnight Soxhlet treatment in tetrahydrofuran was performed, followed by $scCO_2$ drying, which resulted NDI-COF as a light brown solid with a yield of 54.7 mg.

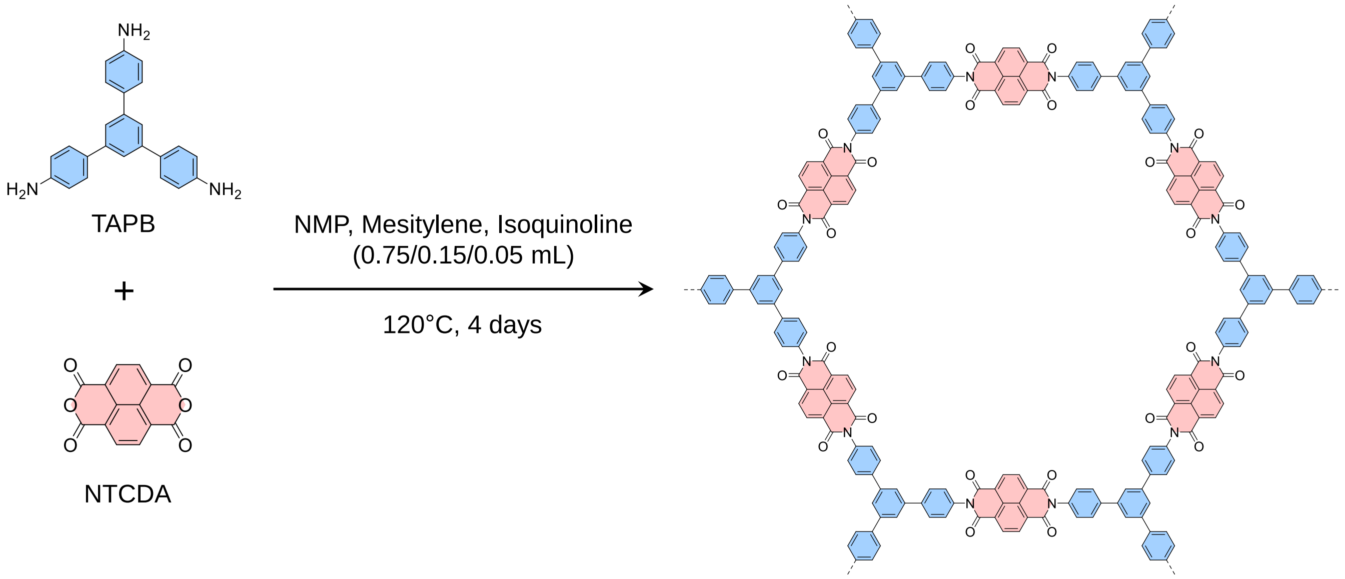

Figure S1. Synthetic procedure and chemical structure of NDI-COF.

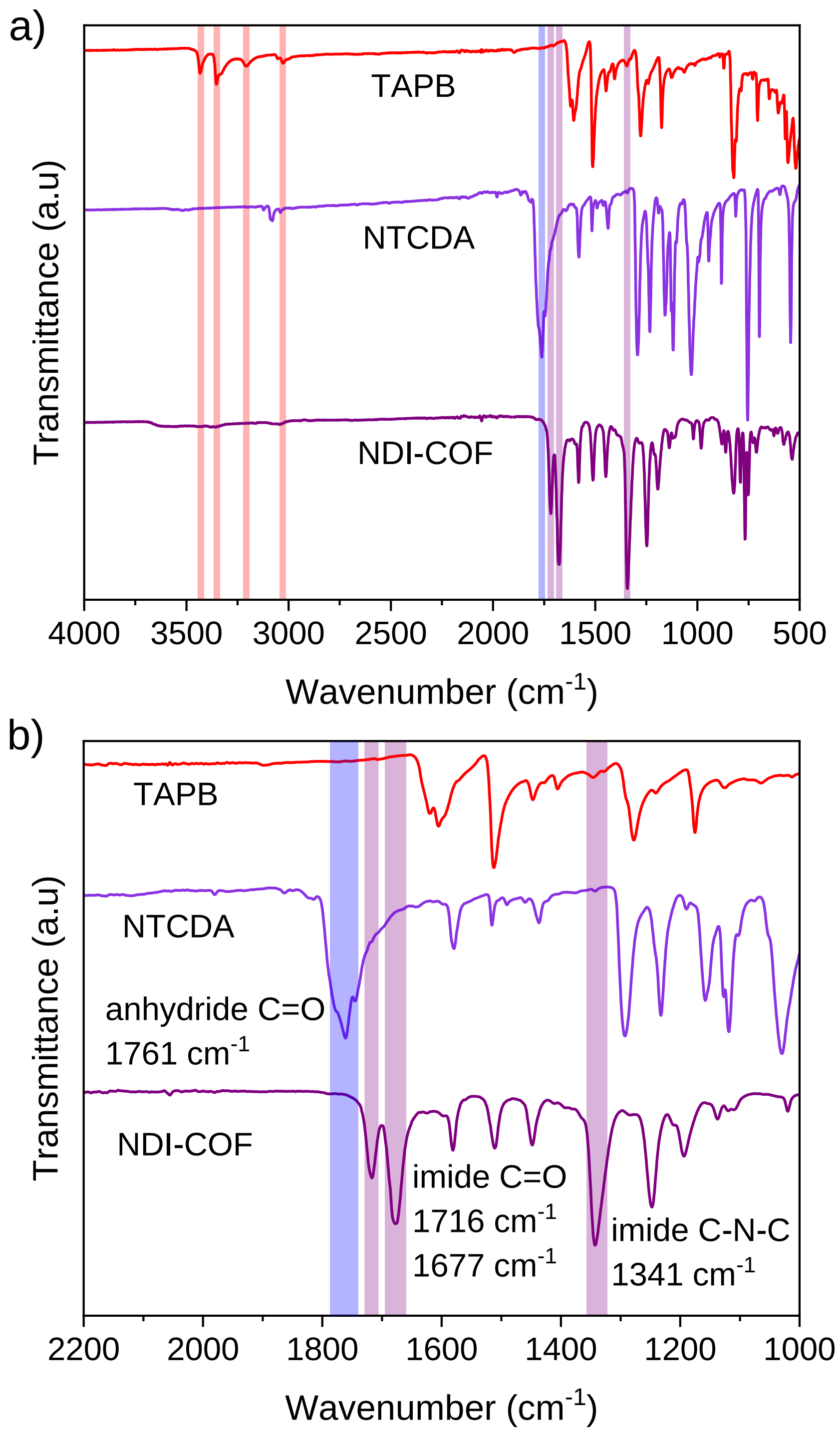

Figure S2. (a) FTIR spectra of TAPB (red), NTCDA (blue), and NDI-COF (purple) highlighting the N-H bands from TAPB (light red), the C=O band from NTCDA (light blue), and the six-membered imide ring bands from NDI-COF (light purple). (b) Enlarged section of the FTIR spectra highlighting the C=O band from NTCDA (light blue) and the six-membered imide ring bands from NDI-COF (light purple).

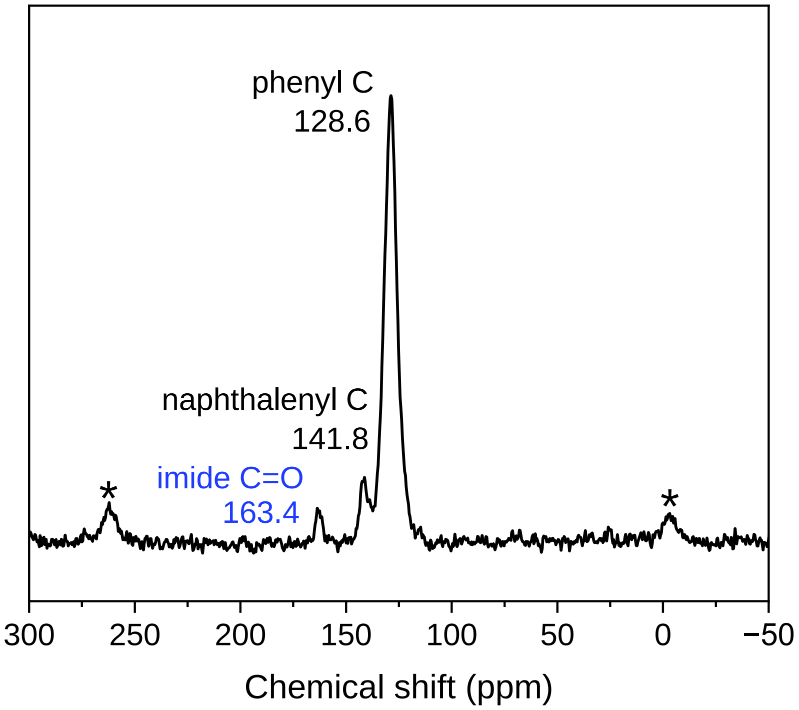

Figure S3. ^{13}C–CP/MAS-NMR spectrum of NDI-COF. Asterisks indicate spinning side bands.

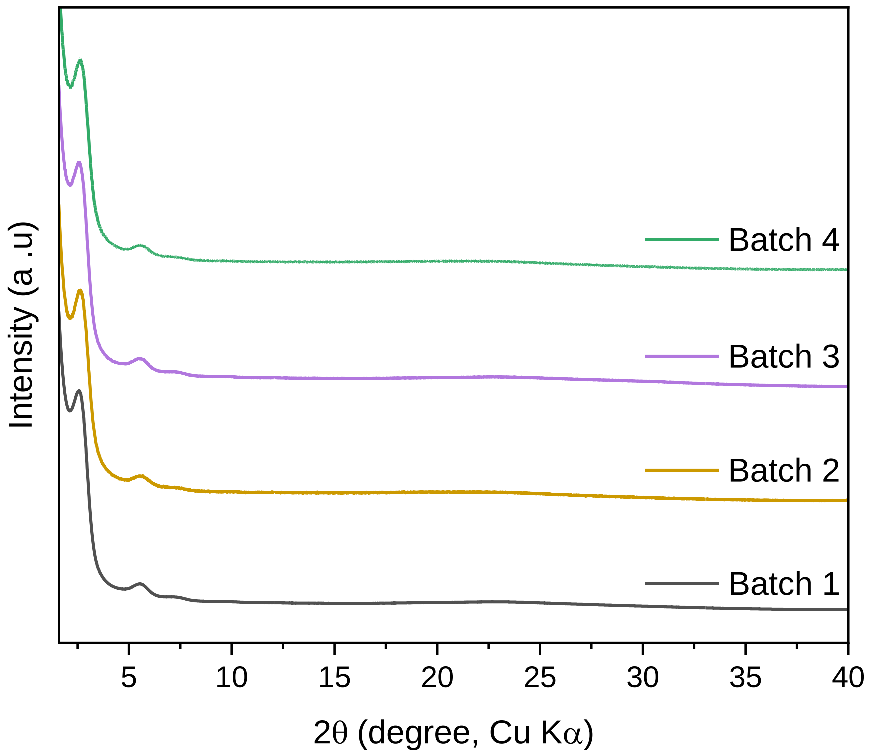

Figure S4. Comparison of PXRD patterns of NDI-COF synthesized in different batches. The solvothermal synthesis method is reproducible and returns COFs with similar spectroscopic features.

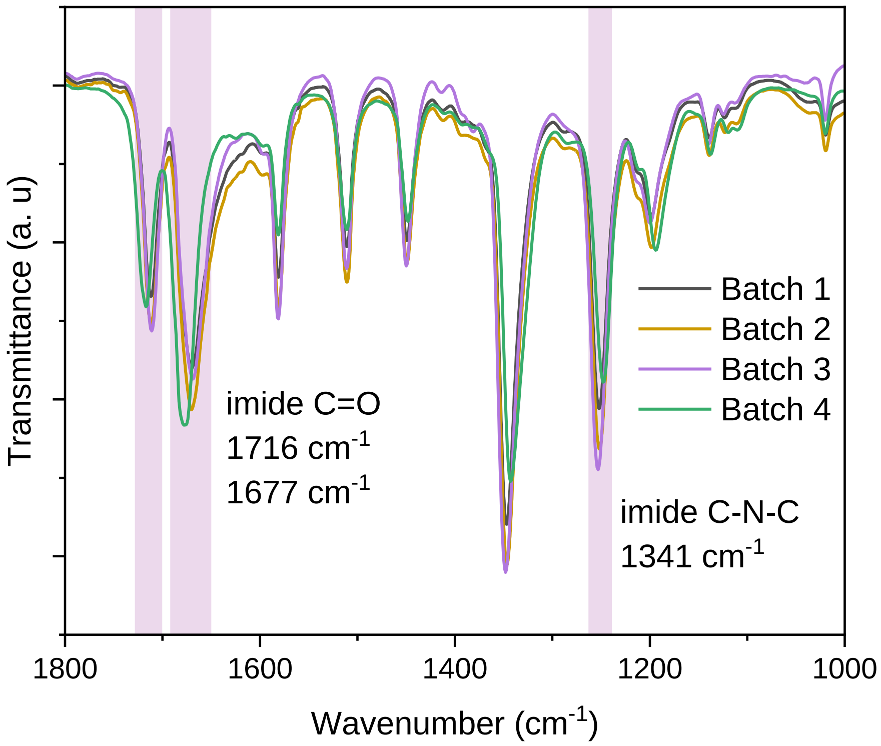

Figure S5. Comparison of FTIR spectra of NDI-COF synthesized in different batches showing the characteristic imide features.

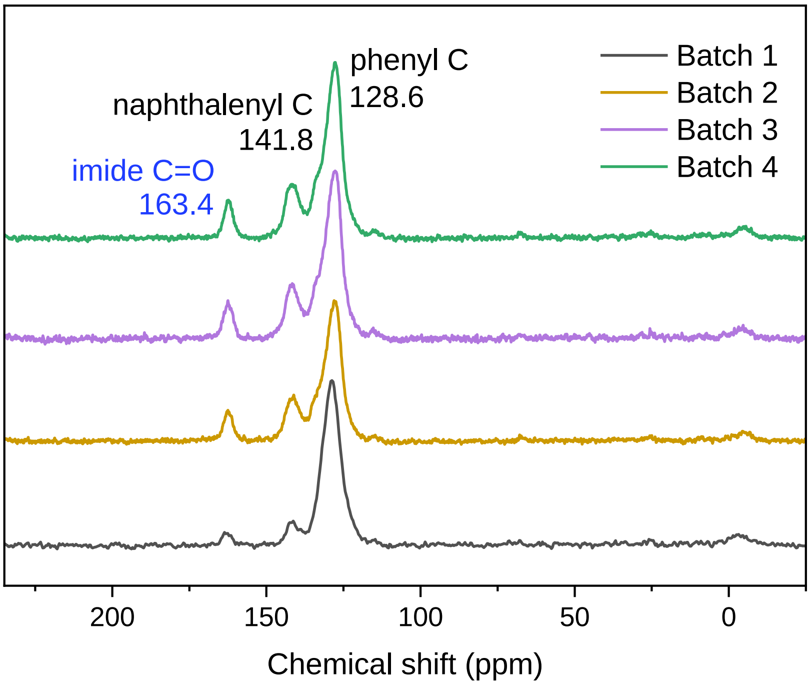

Figure S6. Comparison of ^{13}C–CP/MAS-NMR spectra of NDI-COF synthesized in different batches showing the characteristic imide features.

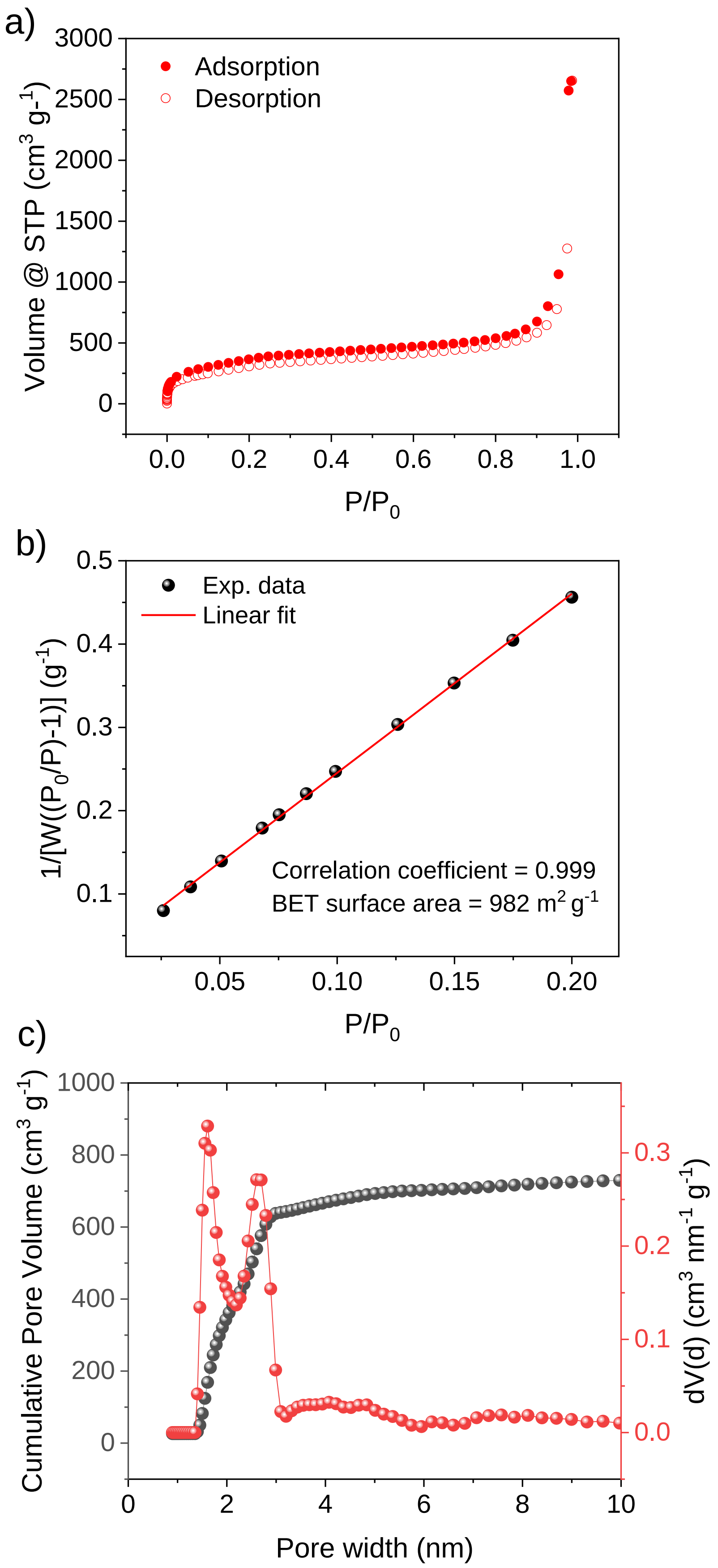

Figure S7. (a) Ar sorption isotherm, (b) BET plot showing the relative pressure (P/P_0) vs. $1/[W((P_0/P)-1)]$ BET function, and (c) cumulative pore volume and pore size distribution of NDI-COF.

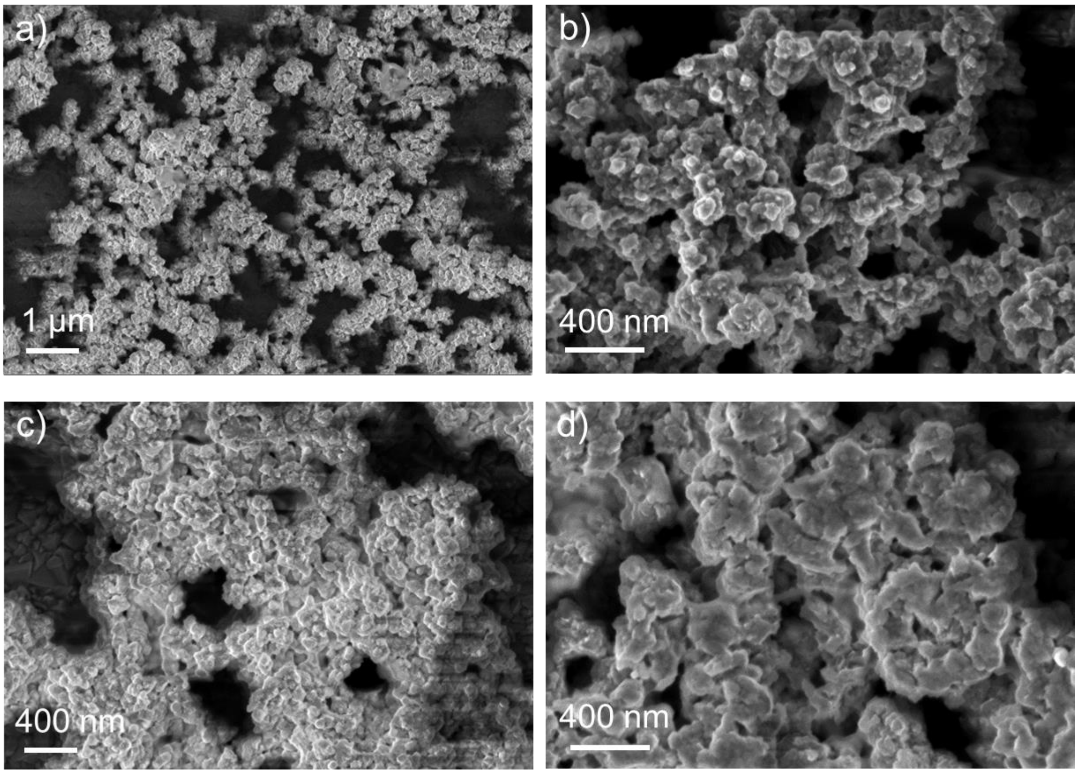

Figure S8. InLens SEM images of NDI-COF at various magnifications.

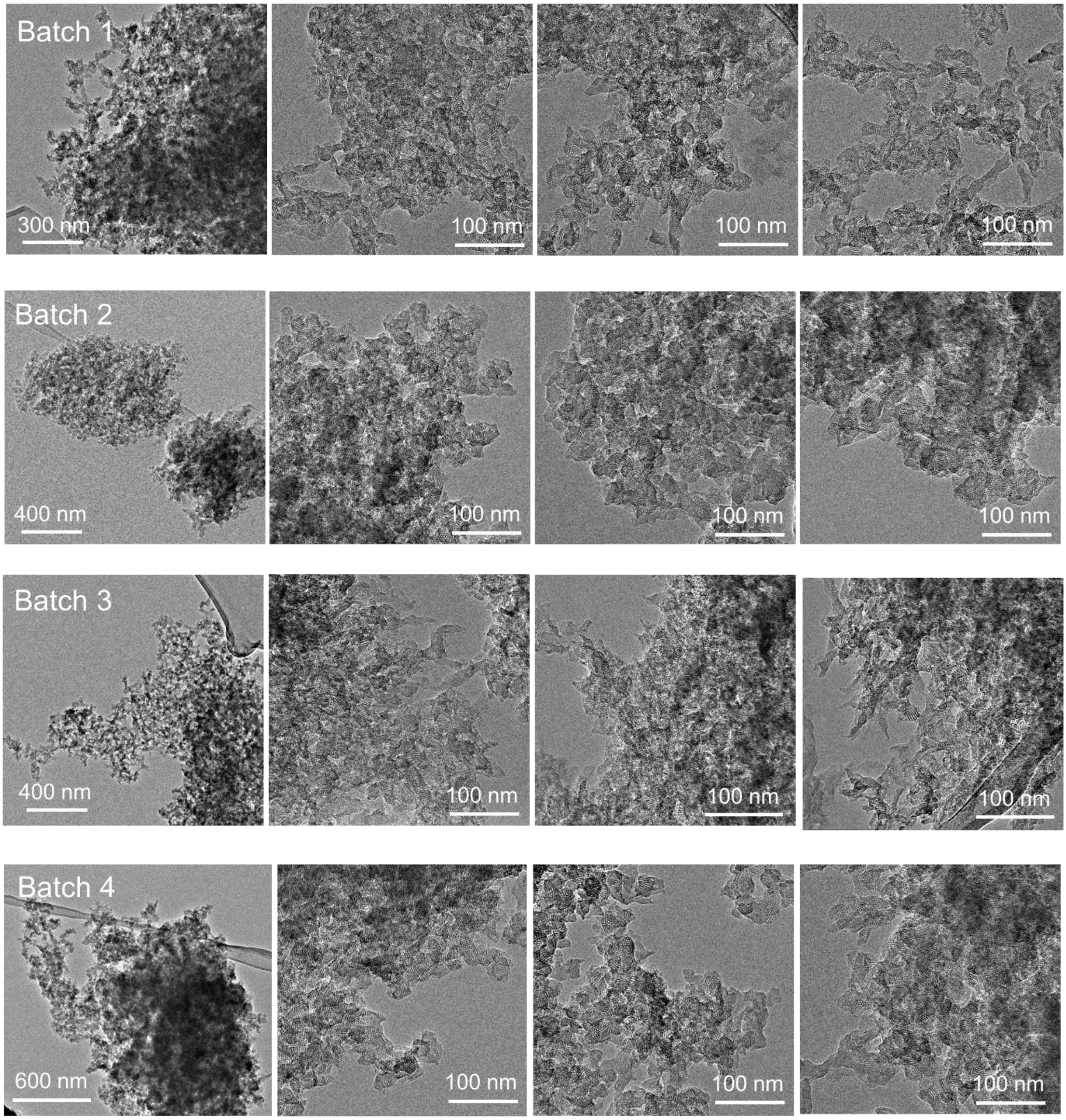

Figure S9. TEM images of NDI-COF synthesized in different batches at various magnifications. The average crystalline domain sizes are (25 ± 9 nm).

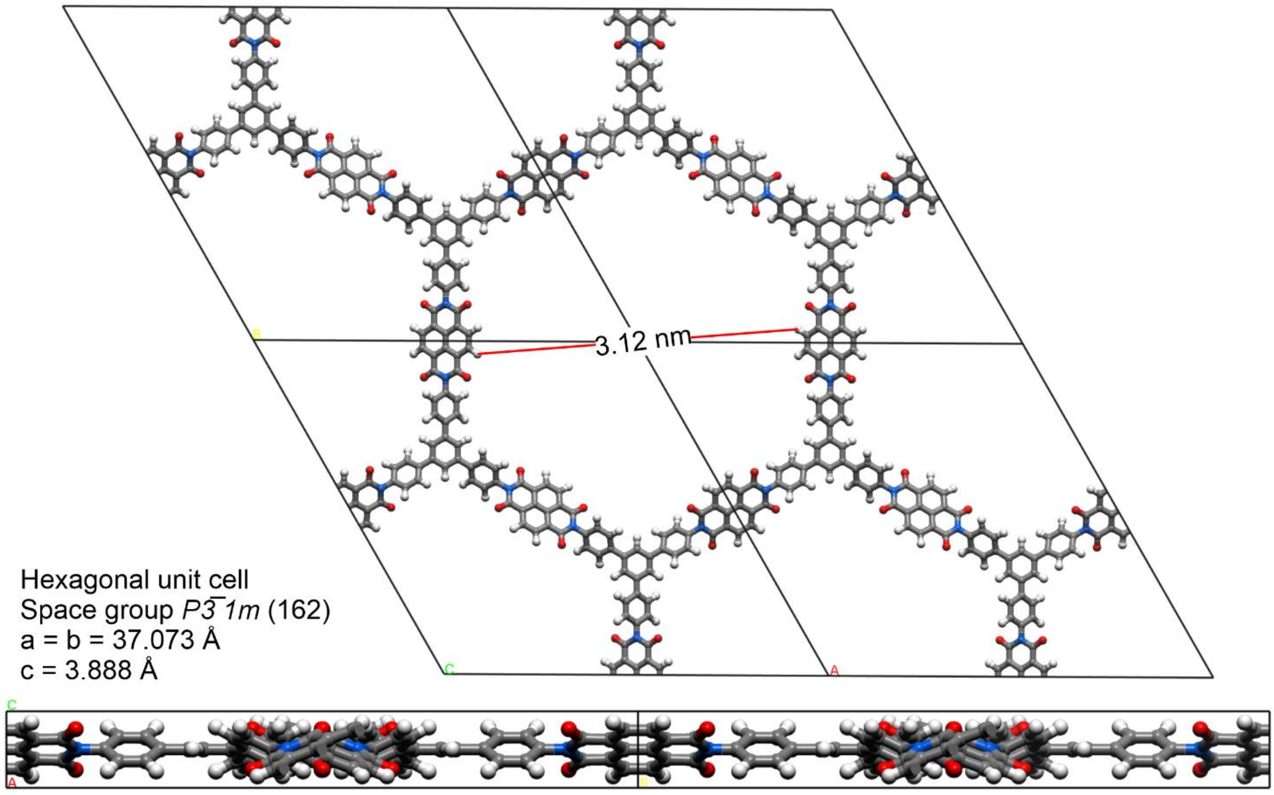

Figure S10. Simulated NDI-COF structure showing different orientations.

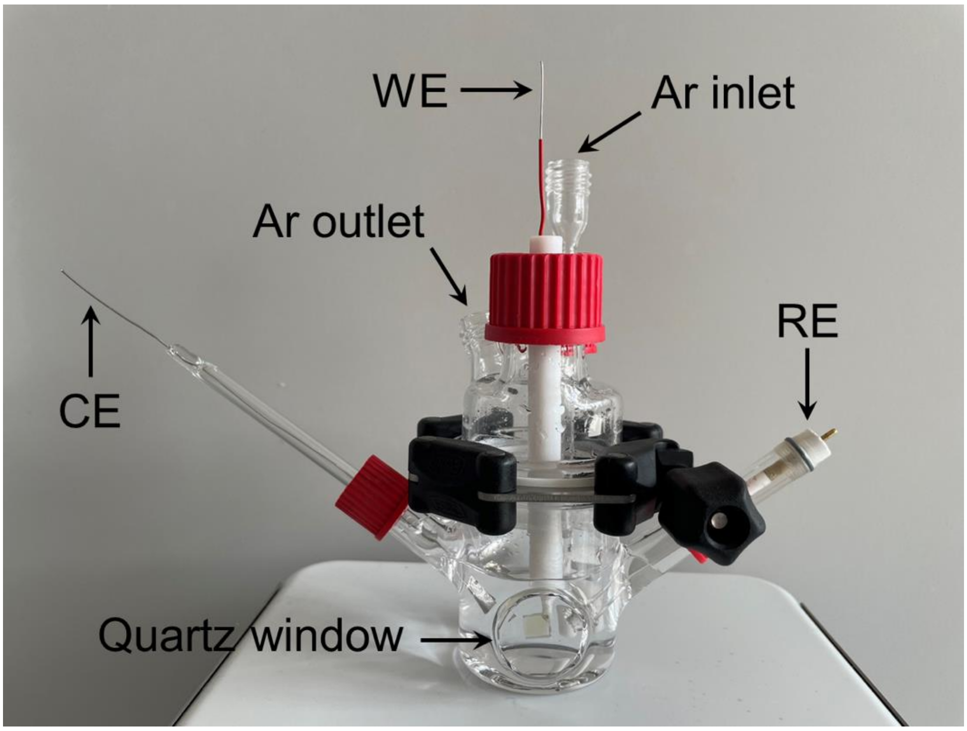

Figure S11. Photograph of the home-made reactor used for photo(electrochemical) measurements using a three-electrode setup. For photoelectrochemical measurements, light is illuminated through the quartz window on the back side of the FTO electrode.

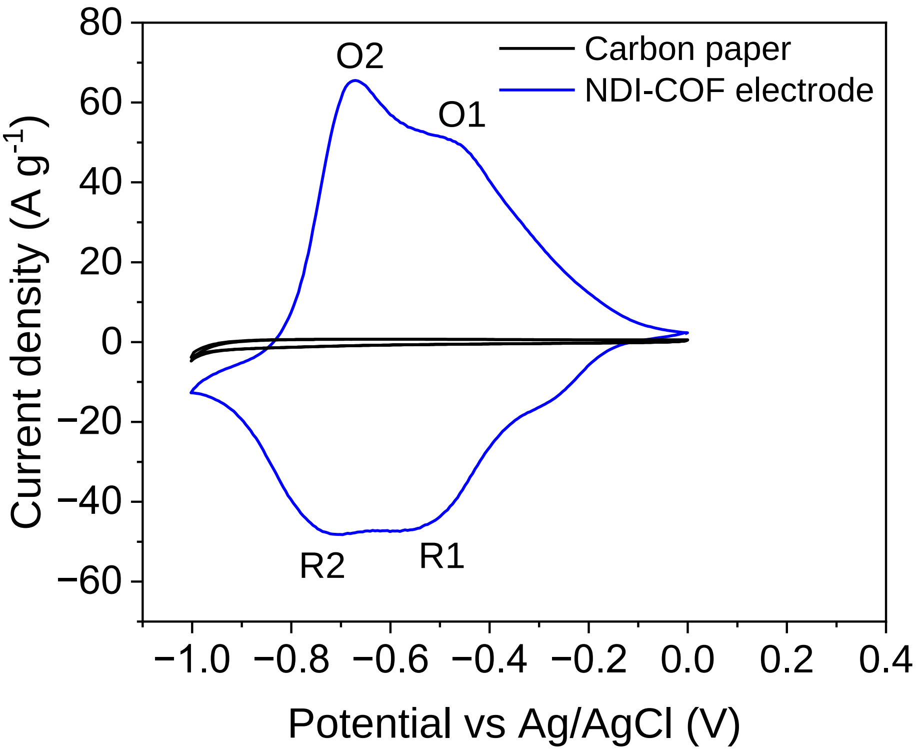

Figure S12. Cyclic voltammetry curves of carbon black on a carbon paper electrode and NDI-COF on a carbon paper electrode at a scan rate of 10 mV s^{-1}.

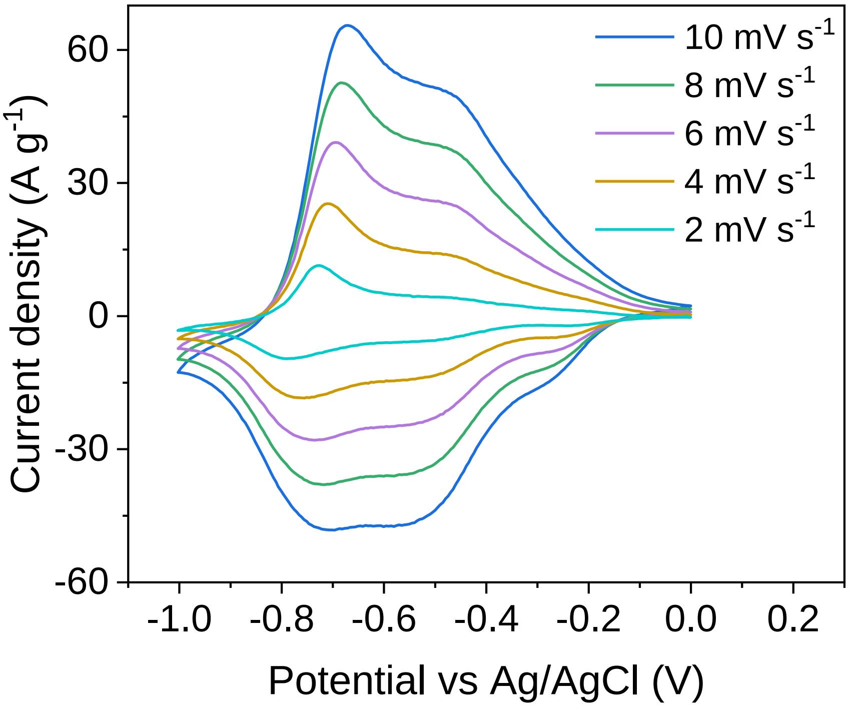

Figure S13. Cyclic voltammetry measurement of NDI-COF on carbon electrode at various scan rates ranging from 2–10 mV s^{-1}.

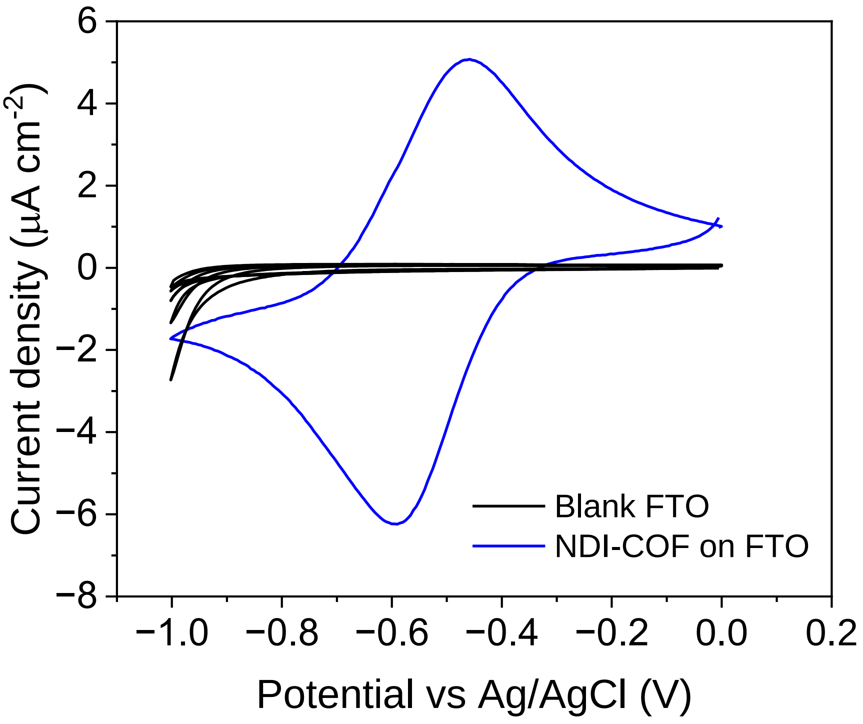

Figure S14. Cyclic voltammetry curves of blank FTO electrode and NDI-COF on FTO electrode at a scan rate of 10 mV s^{-1}.

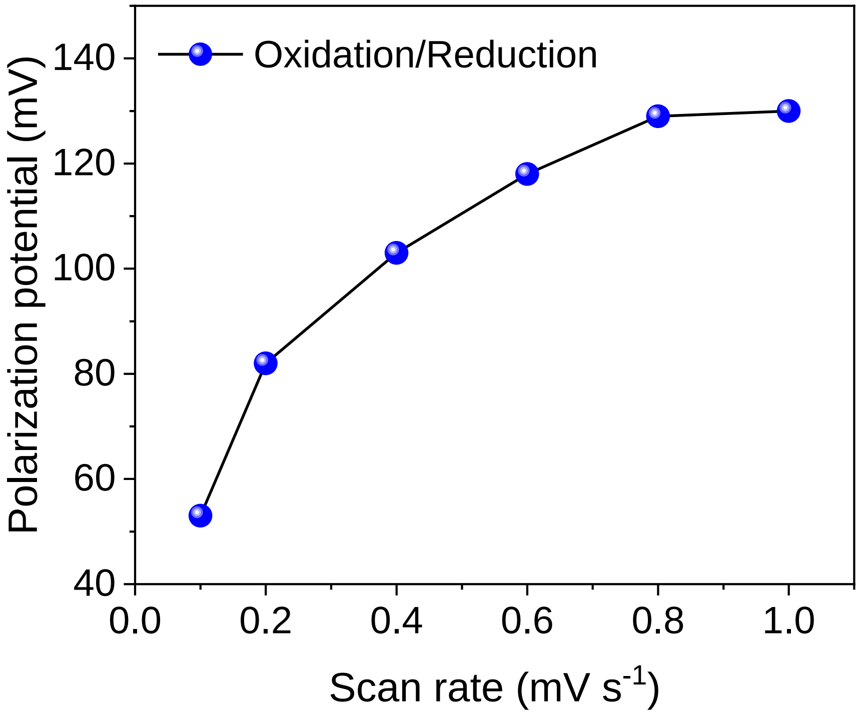

Figure S15. Polarization potential of the of redox peak (Oxidation/Reduction) in the CV curve of NDI-COF on FTO electrode.

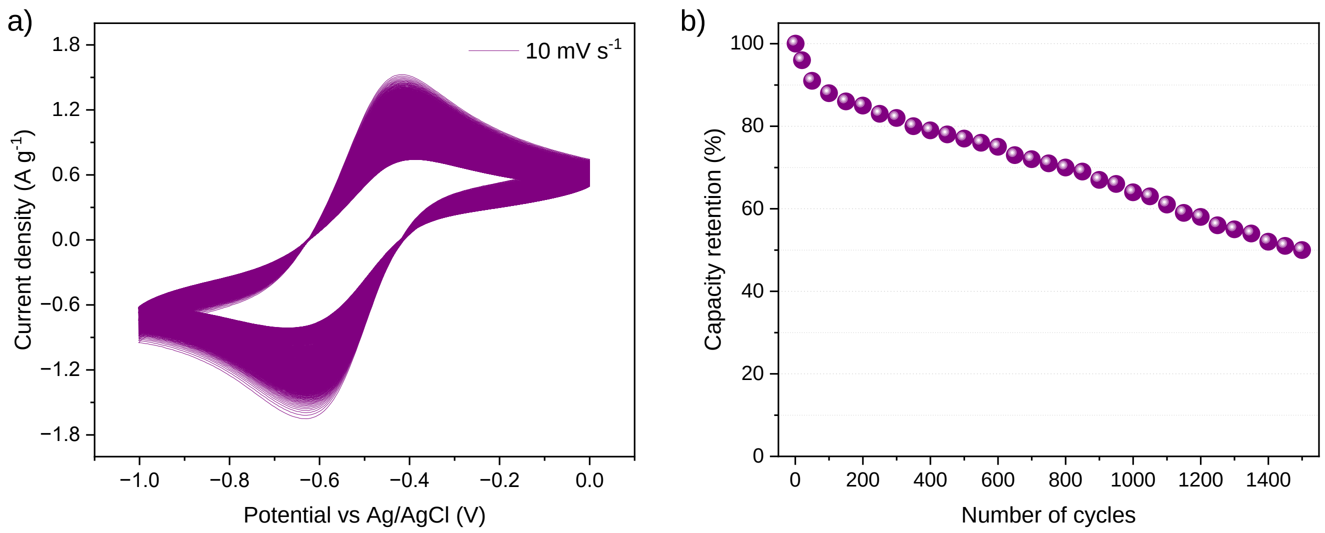

Figure S16. (a) Cyclic voltammetry measurements of NDI-COF on FTO electrode for 1500 cycles at a scan rate of 10 mV s^{-1}, showing good electrode stability, along with a reversible redox process. (b) Cycling performance of NDI-COF showing capacity retention of more than 50% over 1500 cycles.

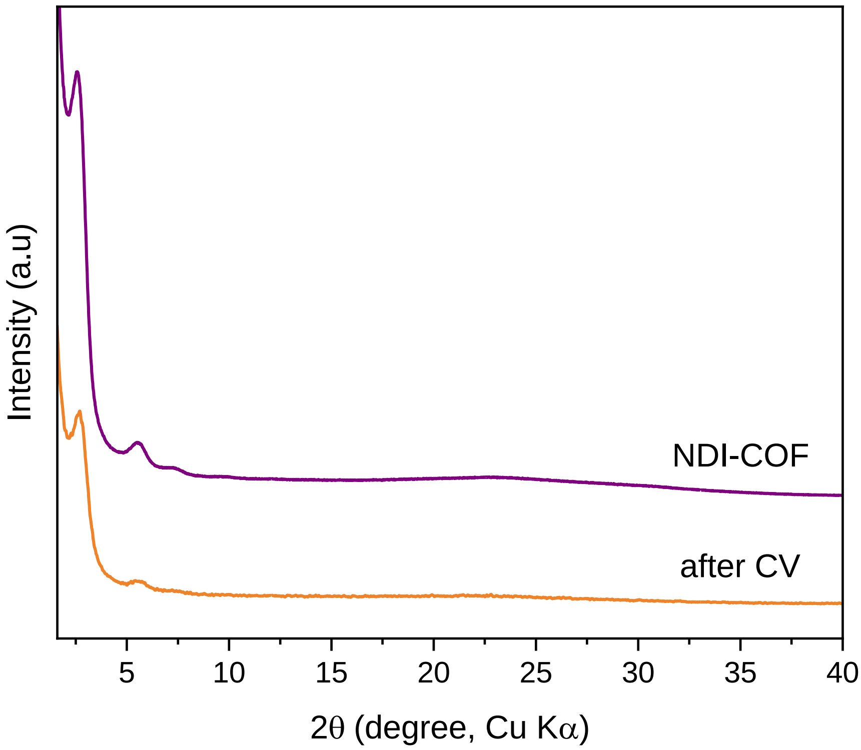

Figure S17. Comparison of PXRD patterns shows considerable loss of crystallinity of NDI-COF after multiple cycles of cyclic voltammetry measurements.

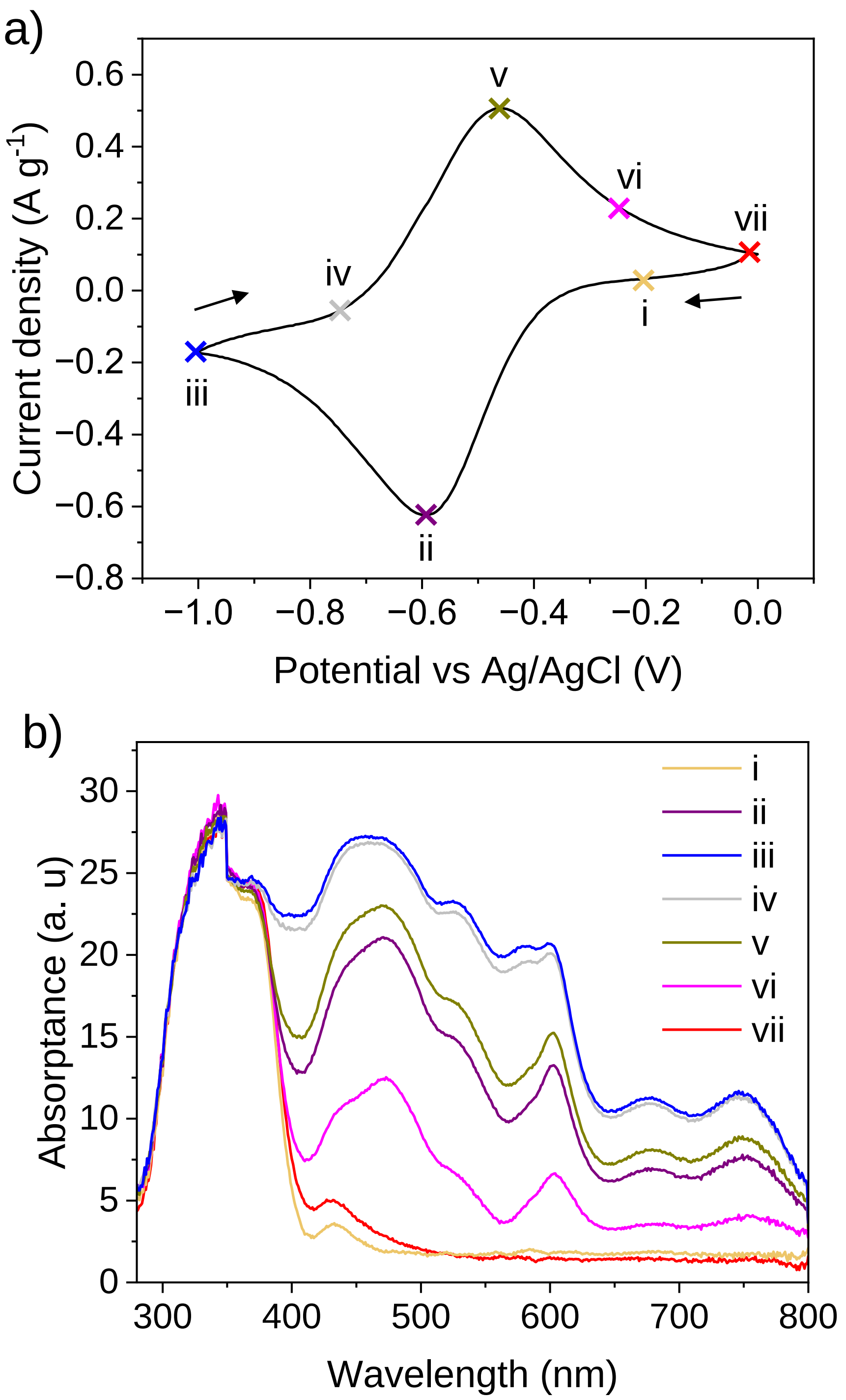

Figure S18. (a) A representative CV curve showing the potentials used for spectroelectrochemical measurements during cathodic and anodic scans. (b) UV-visible absorptance spectra collected at marked potentials shows the reversible reduction and oxidation of NDI-COF.

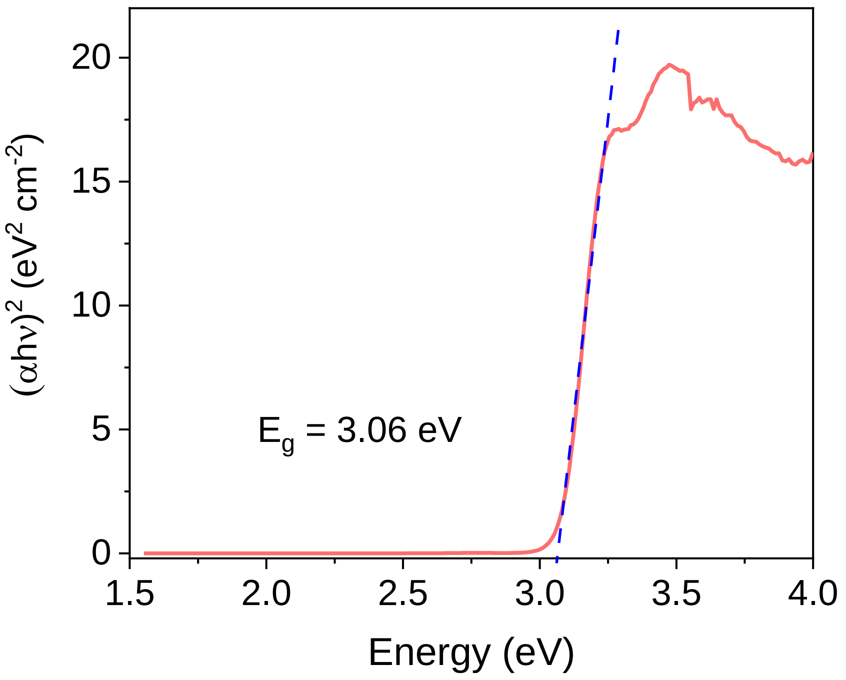

Figure S19. The Tauc plot of NDI-COF obtained from UV-vis spectrum and extracted optical band gaps.

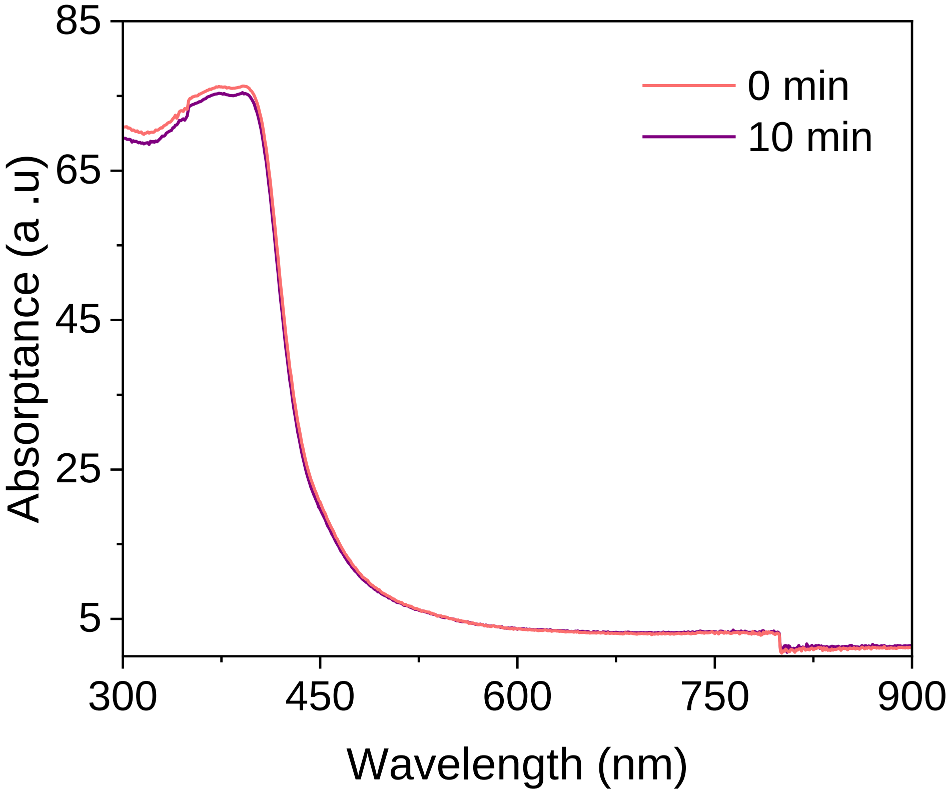

Figure S20. UV-vis absorptance spectra of NDI-COF suspension in oxygen-free water without any sacrificial electron donor (SED) before and after UV irradiation. In the absence of SED, no signature of the $NDI^{\bullet-}$ radical was observed, suggesting the role of SED as efficient hole quencher to stabilize photoelectrons in the framework.

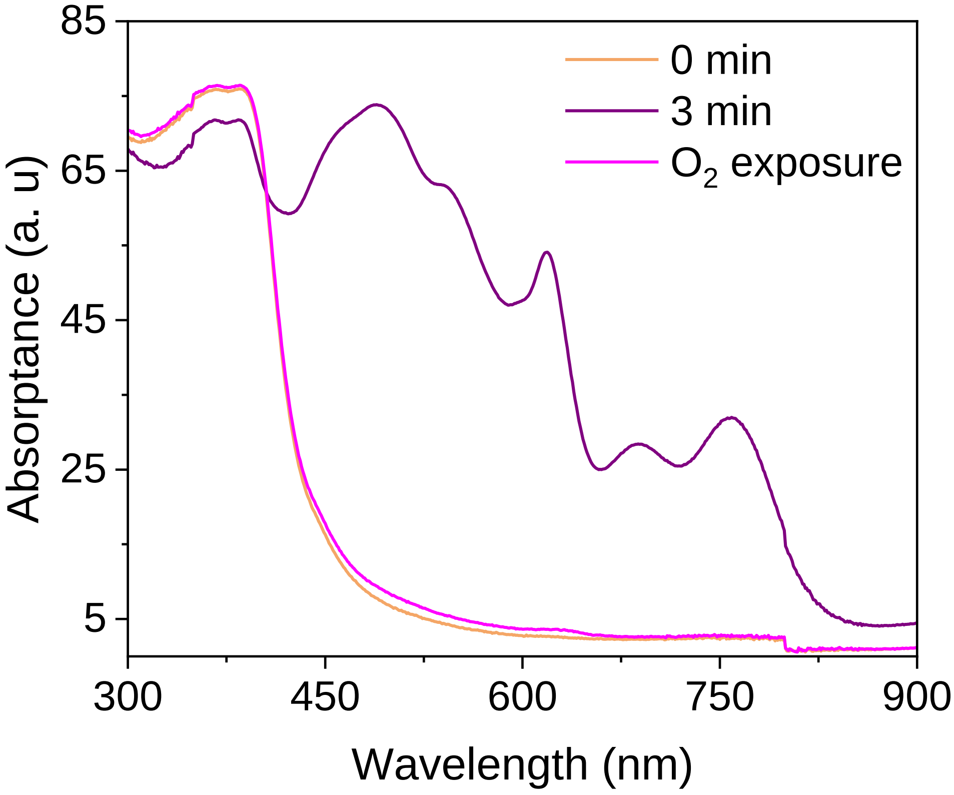

Figure S21. UV-vis absorptance spectra of NDI-COF suspension in oxygen-free water in the presence of 10 mM sacrificial electron donor (SED) 4-MBA before and after 3 min UV irradiation, showing the formation of stable $NDI^{\bullet-}$ radicals after hole quenching. Upon oxygen exposure, the $NDI^{\bullet-}$ radical was quenched and the original spectrum was obtained. This was also marked by the reversal of the brown color of the radical state to the yellow color of the ground state.

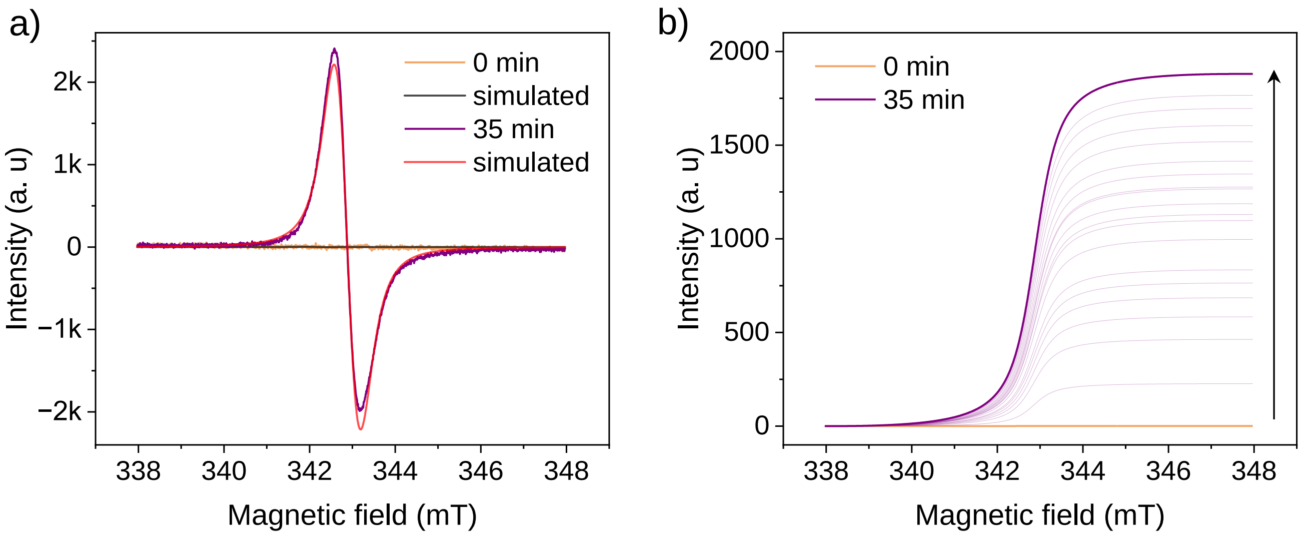

Figure S22. (a) Experimental EPR spectra with the simulated spectra at 0 min and 35 min illumination, showing the formation of the NDI$^{•−}$ radical anion. (b) Double integration of the EPR signals collected at different illumination times (Figure 3d) shows the progress of the NDI$^{•−}$ radical formation.

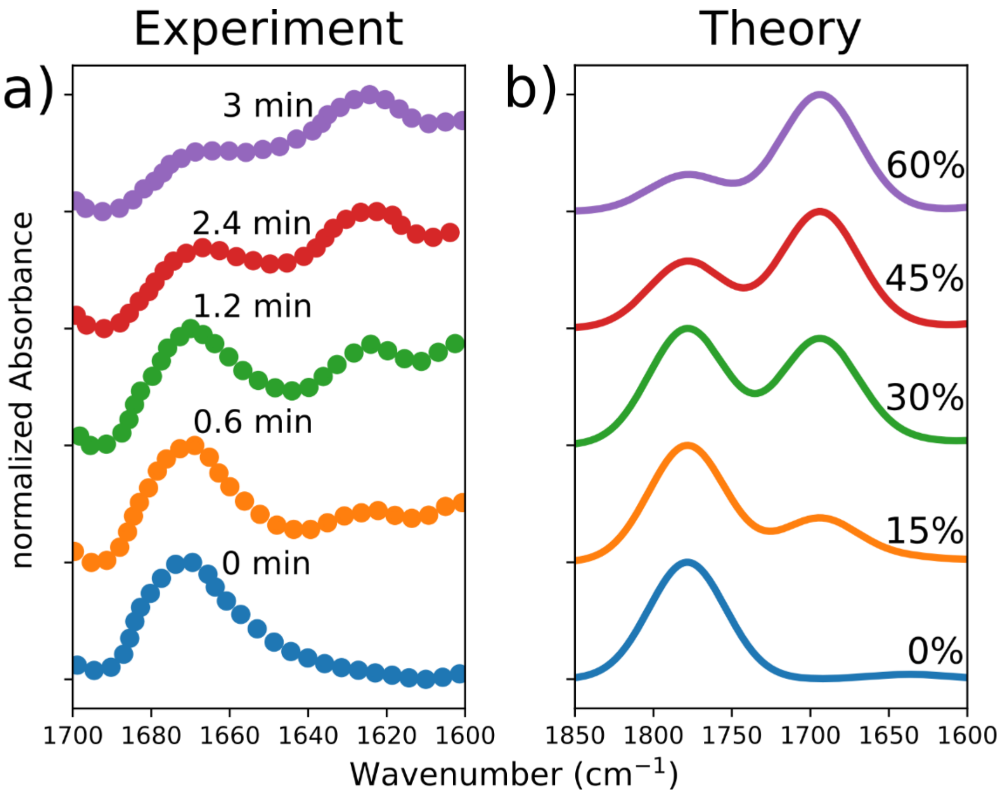

Figure S23. Comparison of the IR absorption spectra corresponding to the C–O stretch modes between experiment (a) and theory (b). In (b) the NDI$^{•-}$/ NDI mixture is indicated as percentage value.

The main absorption features of NDI and NDI$^{•-}$ appearing in the theoretical spectrum are blue-shifted relative to the experiment and show a slightly stronger energetic separation. However, considering that the calculations are based on gas-phase molecules, while in the actual material they are embedded in the COF structure and surrounded by solvent, we find the agreement to be satisfactory. That is, the gradual peak shift with increasing NDI$^{•-}$ concentration in the ATR-IR spectrum is reproduced.

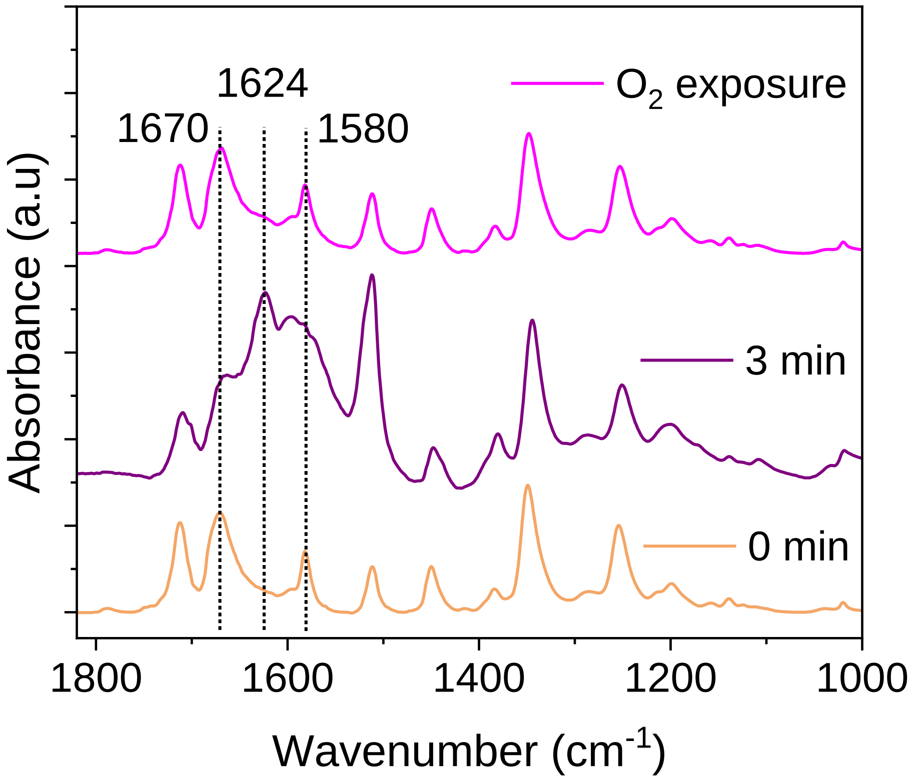

Figure S24. ATR-IR absorbance spectra of NDI-COF showing the evolution of $NDI^{•-}$ radical anion formation upon 3 min illumination. Upon O_2 exposure, the signal disappears due to quenching of the $NDI^{•-}$ radical. This could be reproduced multiple times, indicating that the structural integrity of NDI-COF is maintained.

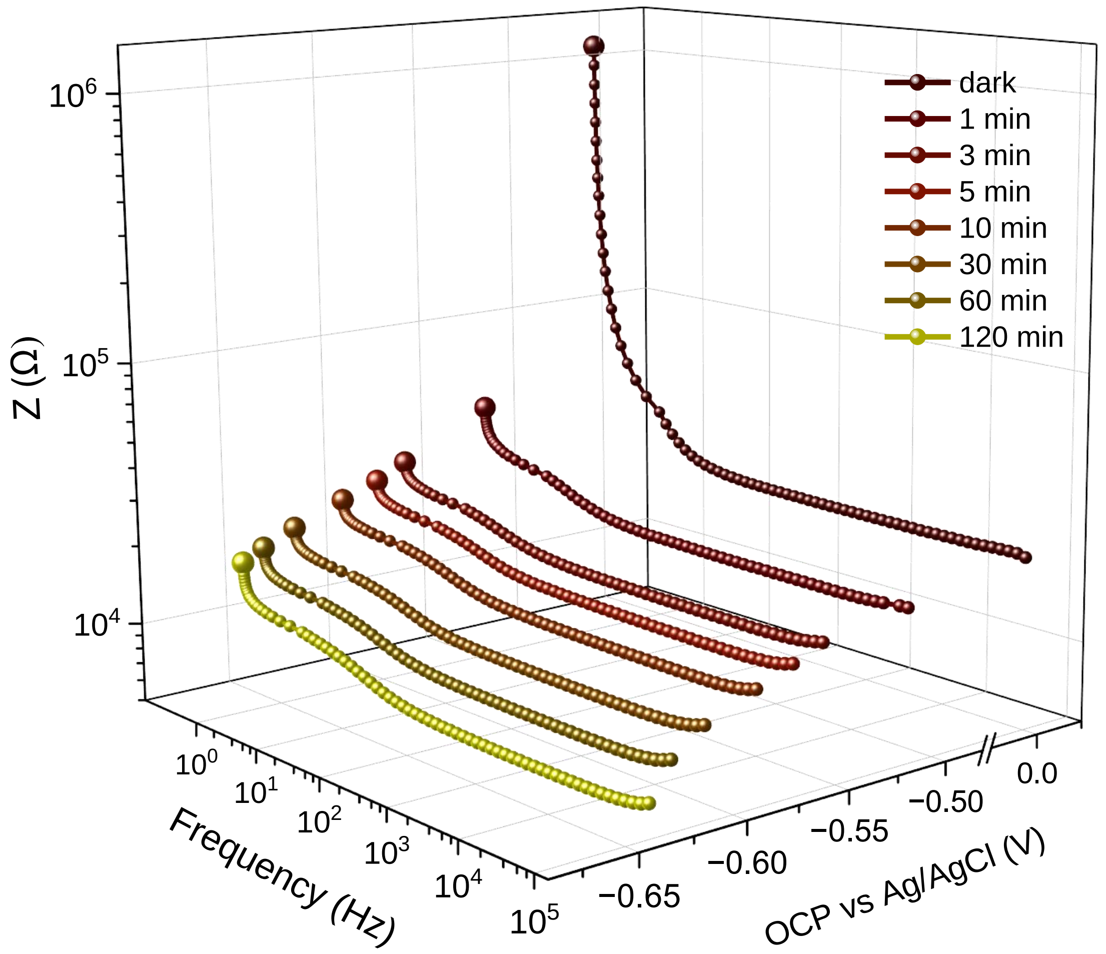

Figure S25. Bode plots of NDI-COF film at different open circuit potentials achieved after 1 sun illumination for different time duration. The measurements were performed in oxygen free water containing 10 mM 4-MBA as sacrificial electron donor. The impedance data point at the frequency of 0.01 Hz was magnified for each measurement.

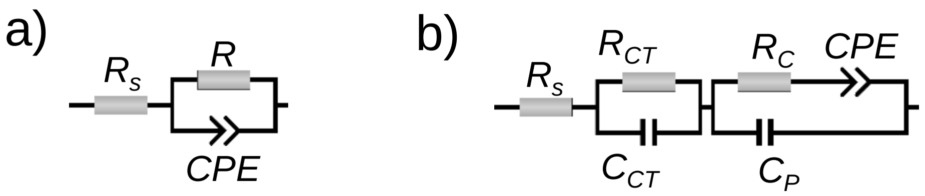

Figure S26. The fitted equivalent circuit models used for the NDI-COF photoanode (a) in the dark and (b) under 1 sun illumination as shown in Figure 4f.

Table S1. Fitting results of the EIS measurement in the dark as shown in Figure 4f.

Parameters	Value	Fit Error	Fit Error (%)
R_S (Ω)	22015	87.7	0.4
R_C (Ω)	1.822E+7	4.76E+6	26
CPE Q 1	8.797E-6	4.84E-8	0.55
CPE Alpha 1	0.83737	0.00309	0.37

Table S2. Fitting results of the EIS measurement after 1 sun illumination as shown in Figure 4f.

Parameters	Value	Fit Error	Fit Error (%)
R_S (Ω)	6206.6	9.26	0.15
R_{CT} (Ω)	354.16	27.4	7.7
C_{CT} (Ω)	4.148E-6	3.92E-7	9.4
R_C (Ω)	1867	33	1.8
CPE Q 1	0.000483	7.84E-6	1.6
CPE Alpha 1	0.41729	0.00497	1.2
C_C (Ω)	8.402E-6	3.06E-7	3.6

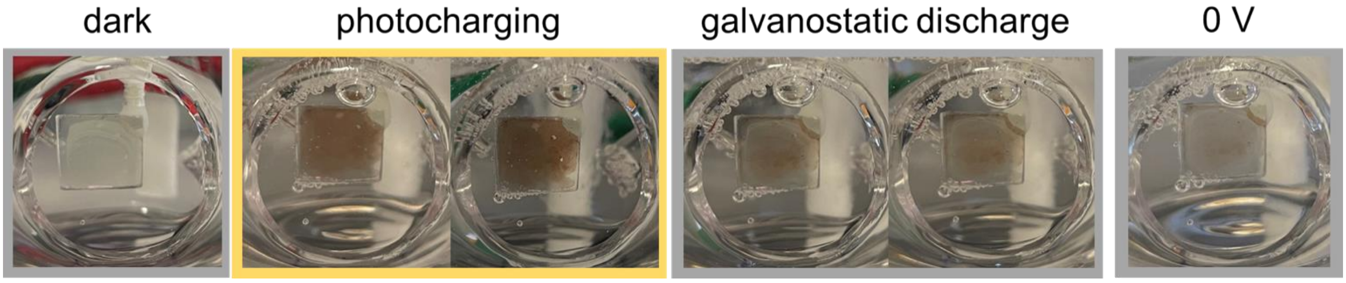

Figure S27. A typical photocharging and electrical discharging experiment in oxygen free 10 mM 4-MBA aqueous electrolyte showing the color change of the photoanode.

After Ar purging for 10 minutes, when a stable OCP is achieved in the dark, the photoanode is charged by 1 sun illumination in the presence of 4-MBA for a certain amount of time (1, 3, 5, 10, 30, 60, 120, 300 min). The photocharging results in the formation of $NDI^{\bullet-}$ indicated by the color change from light yellow to brown. To estimate the charge storage capacity by the respective photocharging experiment, a discharge current (15, 50, 100, 200, 500 mA g^{-1}) is applied to extract the photoaccumulated electrons. This results in the transformation of $NDI^{\bullet-}$ units to pristine NDI, accompanied by color reversal. Note that, due to poor conductivity of the COF film, the photoanode is not fully discharged as seen from the residual color of the electrode. A bias of 0 V is applied for a certain time (~10 min) for a complete discharge, when the photoanode color resembles the original color, before the next experiment.

Table S3. Comparison of charge storage capacity of NDI-COF with reported materials.

Materials	Charge (C g^{-1})	Reference
NDI-COF	136.44	This work
K-PHI	43.56	[21]
Re MOF-253	15	[22]
MnBr-253	42	[23]
$(TBA)^{+}(NbWO_6)^{-}$	11.52	[24]

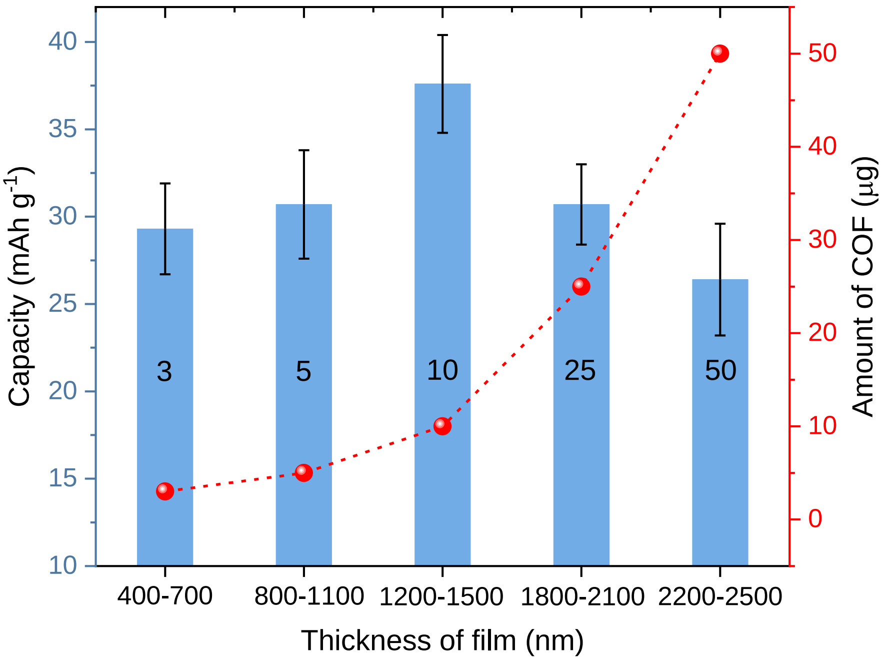

Figure S28. Capacity of the solar battery photoanode as a function of film thickness, which is directly related to the amount of COF loading. The numbers on the bars indicate the amount of COF loading.

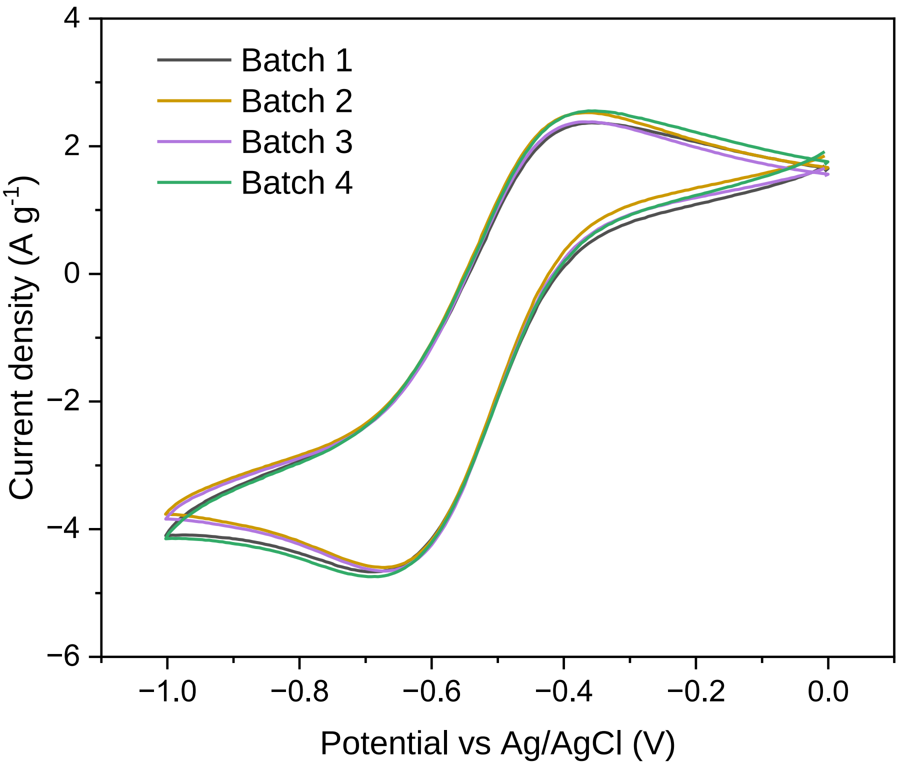

Figure S29. Cyclic voltammetry curves of NDI-COF synthesized in multiple batches on a FTO electrode at a scan rate of 20 mV s^{-1}.

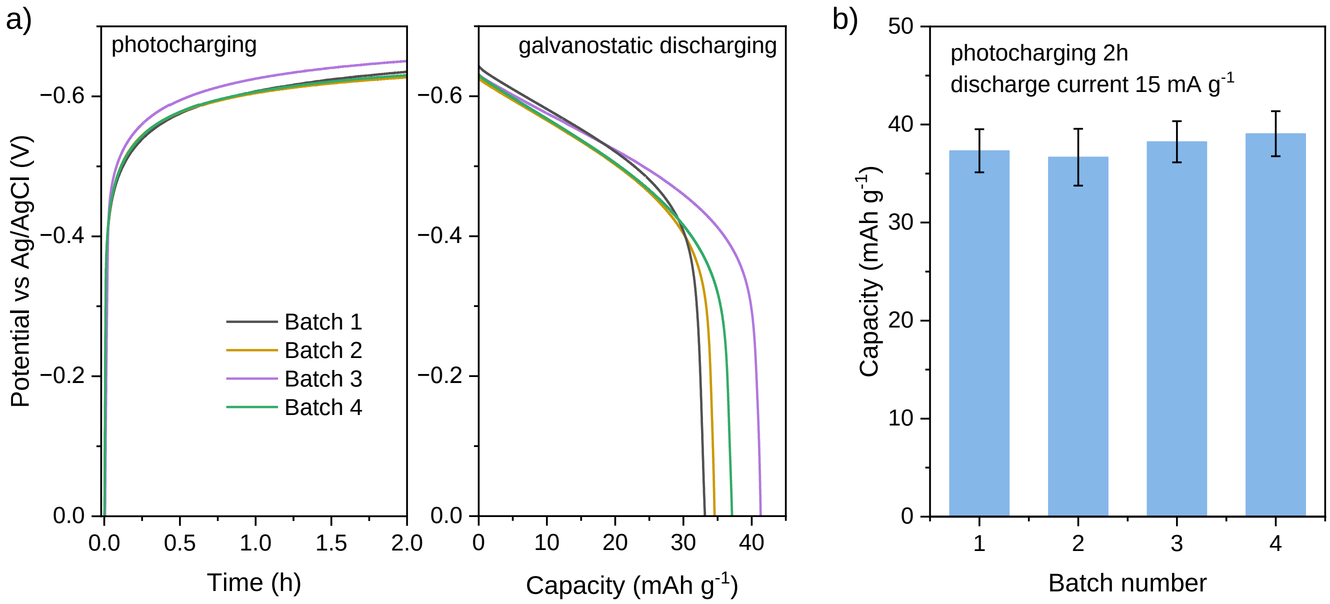

Figure S30. (a) Light-assisted charging profiles for a duration of 2 h, followed by direct electric discharging in the dark at a discharge current density of 15 mA g^{-1} in oxygen free 10 mM 4-MBA aqueous electrolyte. (b) Charge storage capacity of NDI-COF synthesized in multiple batches.

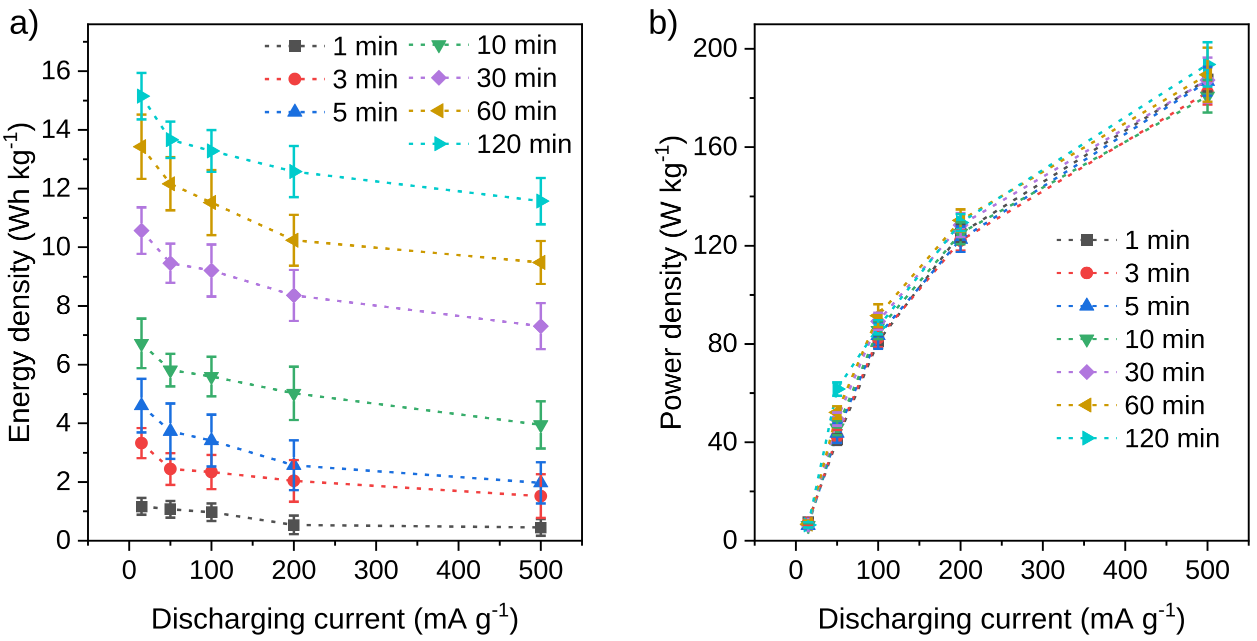

Figure 31. Scaling of (a) energy density and (b) power density for light-assisted charging via 1 sun illumination and subsequent electric discharging in the dark at different discharging currents. Charging is performed via illumination for different time duration and subsequent immediate discharging is carried out with different discharging currents (15, 50, 100, 200, 500 mA g^{-1}).

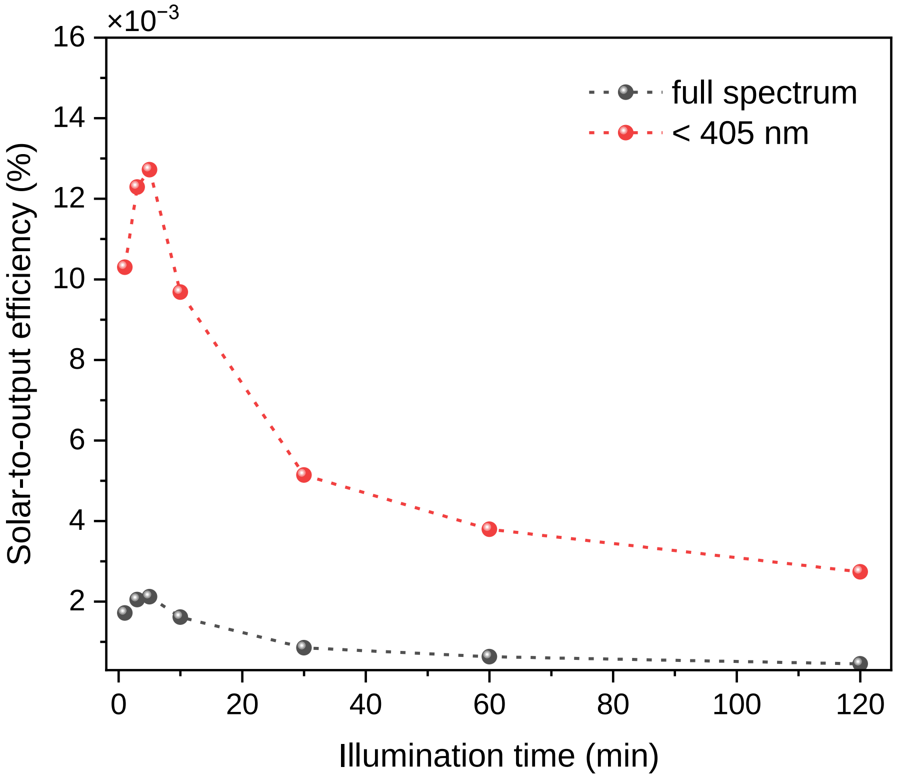

Figure S32. Calculated solar-to-output efficiency. The solar-to-output efficiency was calculated from light charging and electric discharging measurements shown in Figure 5a as a function of illumination time. The light energy was calculated according to AM1.5 G 1 sun illumination, with either the full spectrum or only wavelengths below the bandgap (3.06 eV, corresponding to 405 nm).

The solar-to-output energy conversion efficiency (SOEE) was calculated by the ratio of the output electrical energy over the input solar energy using the equation SOEE (%) = $(\int I_{out} \times E_{out} \times dt)/(P_{in} \times A \times t) \times 100$, where I_{out} is the output current, E_{out} is the output potential, t is the illumination time, P_{in} is the incident solar power (100 mW cm^{-2} for full spectrum and 16.67 mW cm^{-2} for wavelengths below the bandgap) and A is the area of the film.

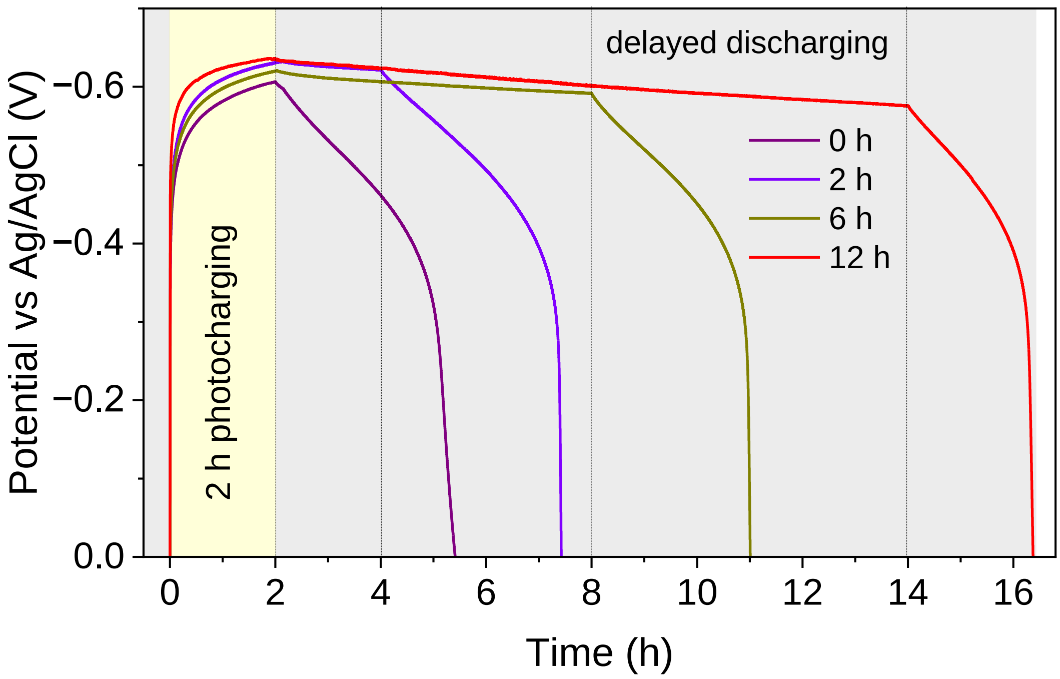

Figure S33. Light-assisted charging profiles for a duration of 2 h, followed by direct and delayed (representative 2, 6, 12 h) electric discharging in the dark. After 2 h photocharging, the dark stability was tested for a certain time period before applying a discharge current density of 15 mA g^{-1} in oxygen free 10 mM 4-MBA aqueous electrolyte.

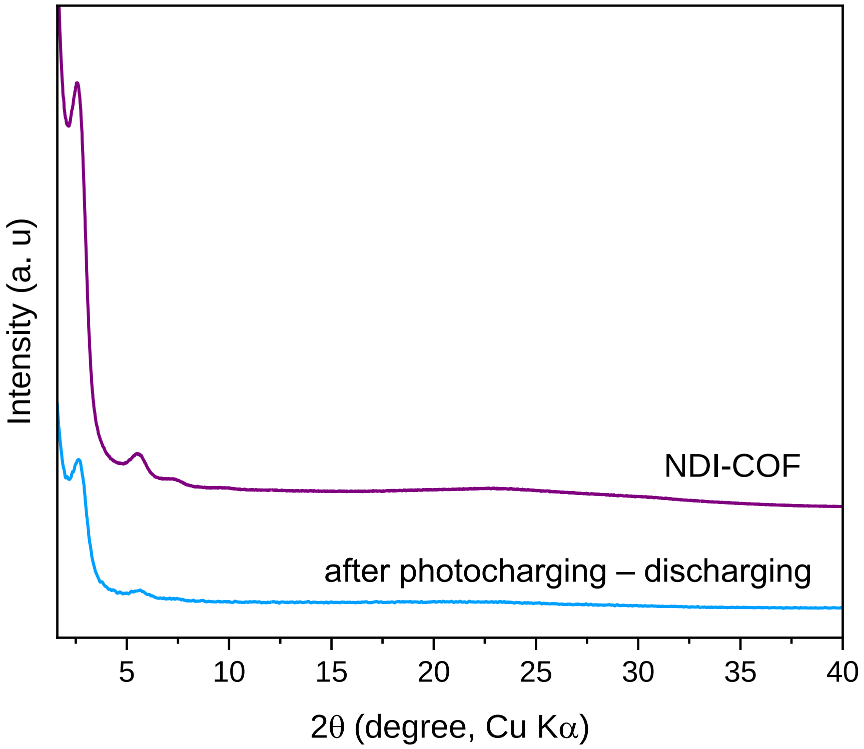

Figure S34. Comparison of PXRD patterns of NDI-COF before and after 30 cycles of photocharging-discharging experiments.

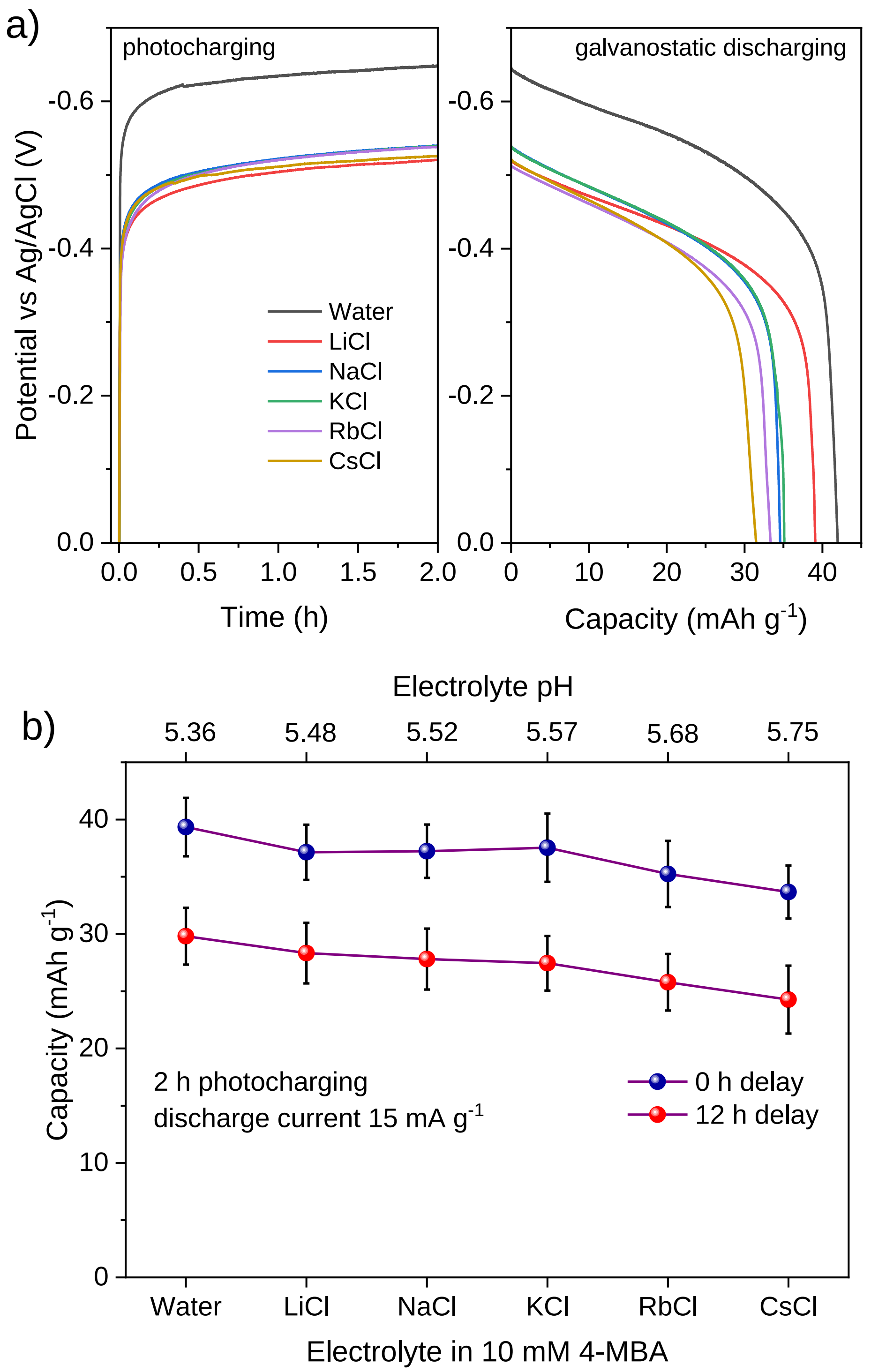

Figure S35. (a) Light-assisted charging profiles for a duration of 2 hours in the absence or presence of various external counter ions and the corresponding galvanostatic discharge profiles. The concentration of external ions is 1 M in a 10 mM aqueous solution of 4-MBA and the discharge current density is 15 mA g^{-1}. (b) Variation in the capacity in the absence or presence of different counter ions (Li^+, Na^+, K^+, Rb^+, Cs^+) after immediate discharging and when discharging was delayed by 12 h.

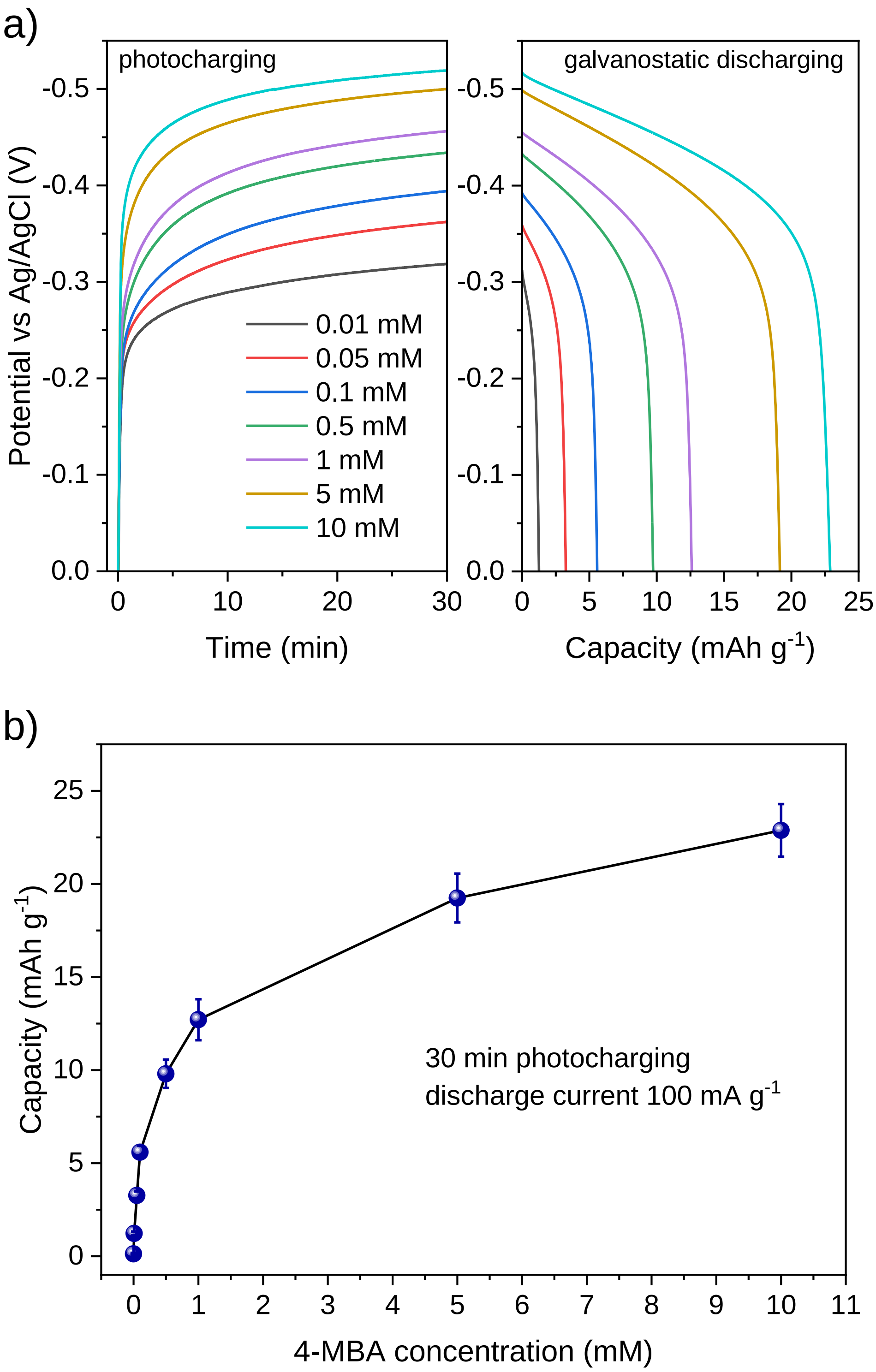

Figure S36. (a) Light-assisted charging profiles for a duration of 30 minutes as a function of electron donor (4-MBA) concentration and the corresponding galvanostatic discharge profiles. The discharge current density is 100 mA g^{-1}. (b) Capacity as a function of 4-MBA concentration shows continuous increase, reaching to a maximum at a concentration of 10 mM 4-MBA in water. The electrolyte is oxygen-free water with an increasing concentration of 4-MBA from 0.01 to 10 mM.

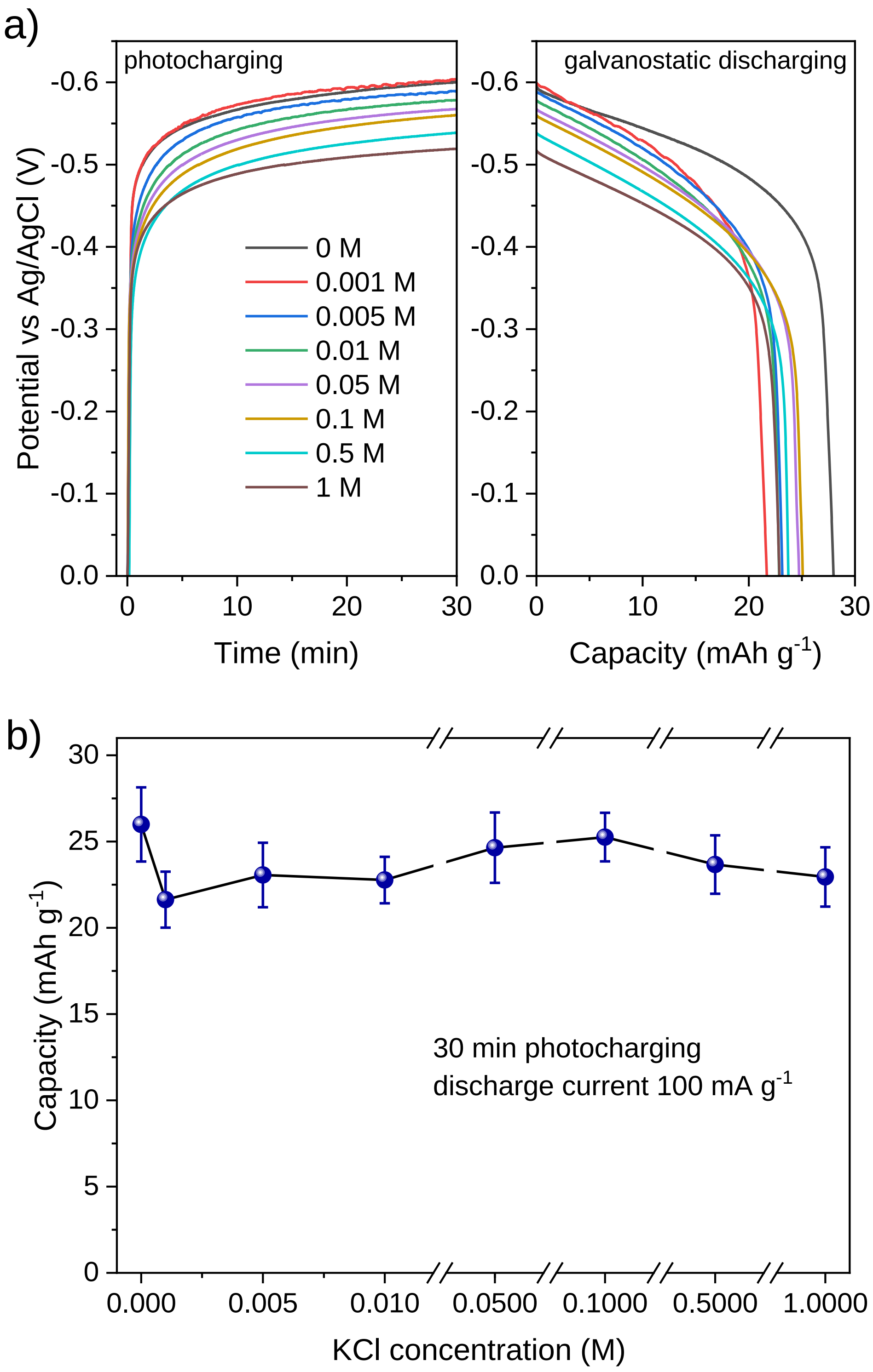

Figure S37. (a) Light-assisted charging profiles for a duration of 30 minutes as a function of external counter ion (K^+) concentration and the corresponding galvanostatic discharge profiles. The discharge current density is 100 mA g^{-1}. (b) Capacity as a function of KCl concentration shows minimal change.

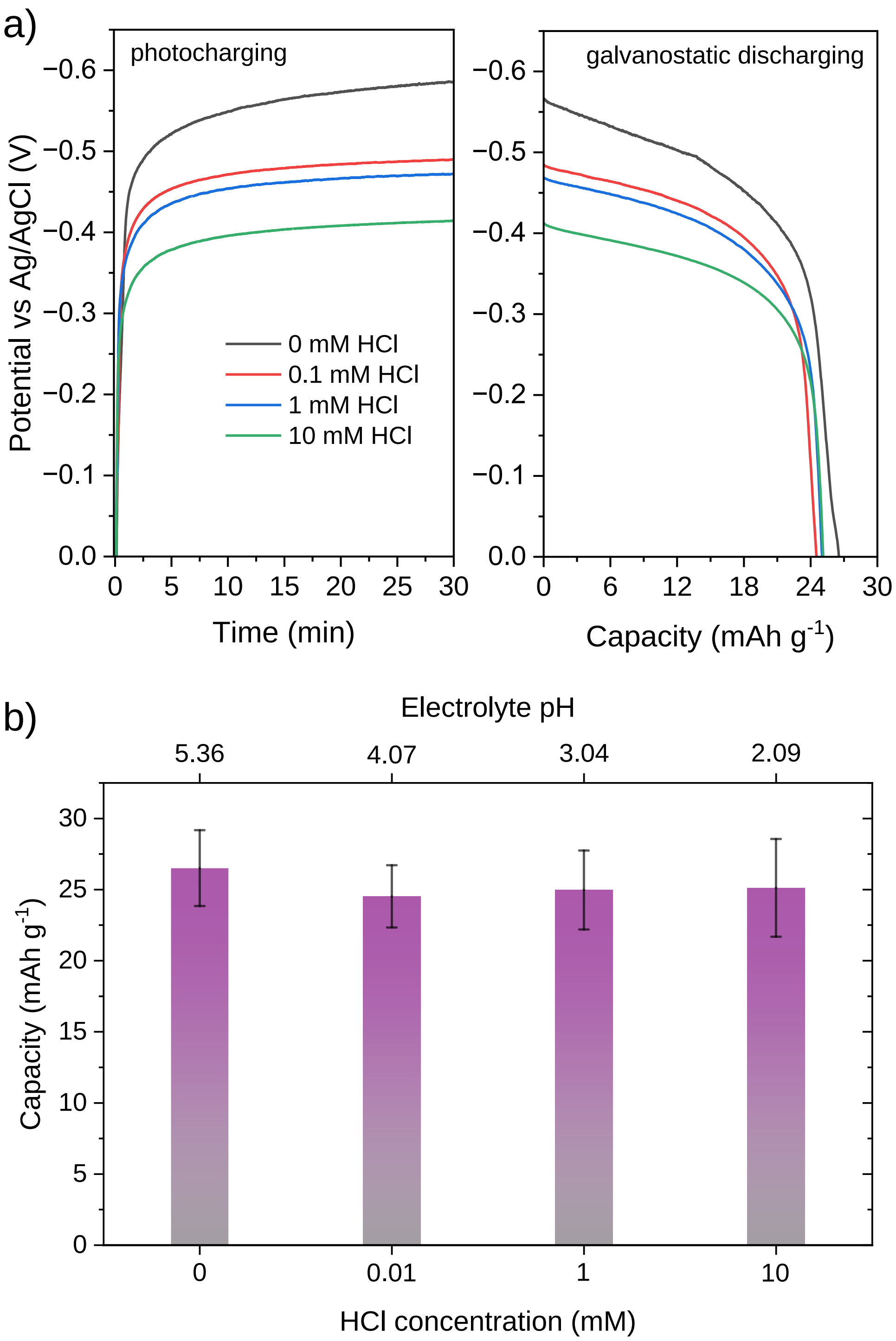

Figure S38. (a) Light-assisted charging profiles for a duration of 30 minutes as a function of external counter ion (H^+) concentration and the corresponding galvanostatic discharge profiles. The discharge current density is 100 mA g^{-1}. (b) Capacity as a function of HCl concentration shows minimal change.

Table S4. Monitoring the photoinduced $NDI^{\bullet-}$ radical formation and the dark stability of NDI-COF suspension in different solvent and electron donor combinations.

**Solvent**	**Dielectric constant (ε)**	**Sacrificial electron donor**		
		**4-MBA**	**TEA**	**TEOA**
Water	80.10	> 48 h	> 48 h	> 48 h
Dimethyl sulfoxide	46.68	< 6 h	< 9 h	< 9 h
Acetonitrile	37.50	X	< 6 h	< 6 h
Dimethylformamide	36.71	< 6 h	< 6 h	< 6 h
Methanol	32.70	X	< 2 h	< 6 h
Ethanol	24.55	< 10 min*	< 6 h	< 6 h
Isopropyl alcohol	19.92	X	< 6 h	< 6 h
1-Butanol	17.51	< 10 min*	< 2 h	< 2 h
Pyridine	13.11	< 30 min*	< 45 min	< 45 min
Dichloromethane	8.93	X	< 1 h	< 1 h
Tetrahydrofuran	7.58	X	< 1 h	< 1 h
Ethyl acetate	7.20	X	< 1 h	< 1 h
Chloroform	4.81	X	< 30 min	< 30 min
Toluene	2.38	X	< 2 h	< 2 h
1,4-Dioxane	2.25	< 30 min	< 2 h	< 3 h

4-Methylbenzylalcohol (**4-MBA**), Triethylamine (**TEA**), Triethanolamine (**TEOA**)

X – represents no $NDI^{\bullet-}$ radical formation

* partial $NDI^{\bullet-}$ radical formation

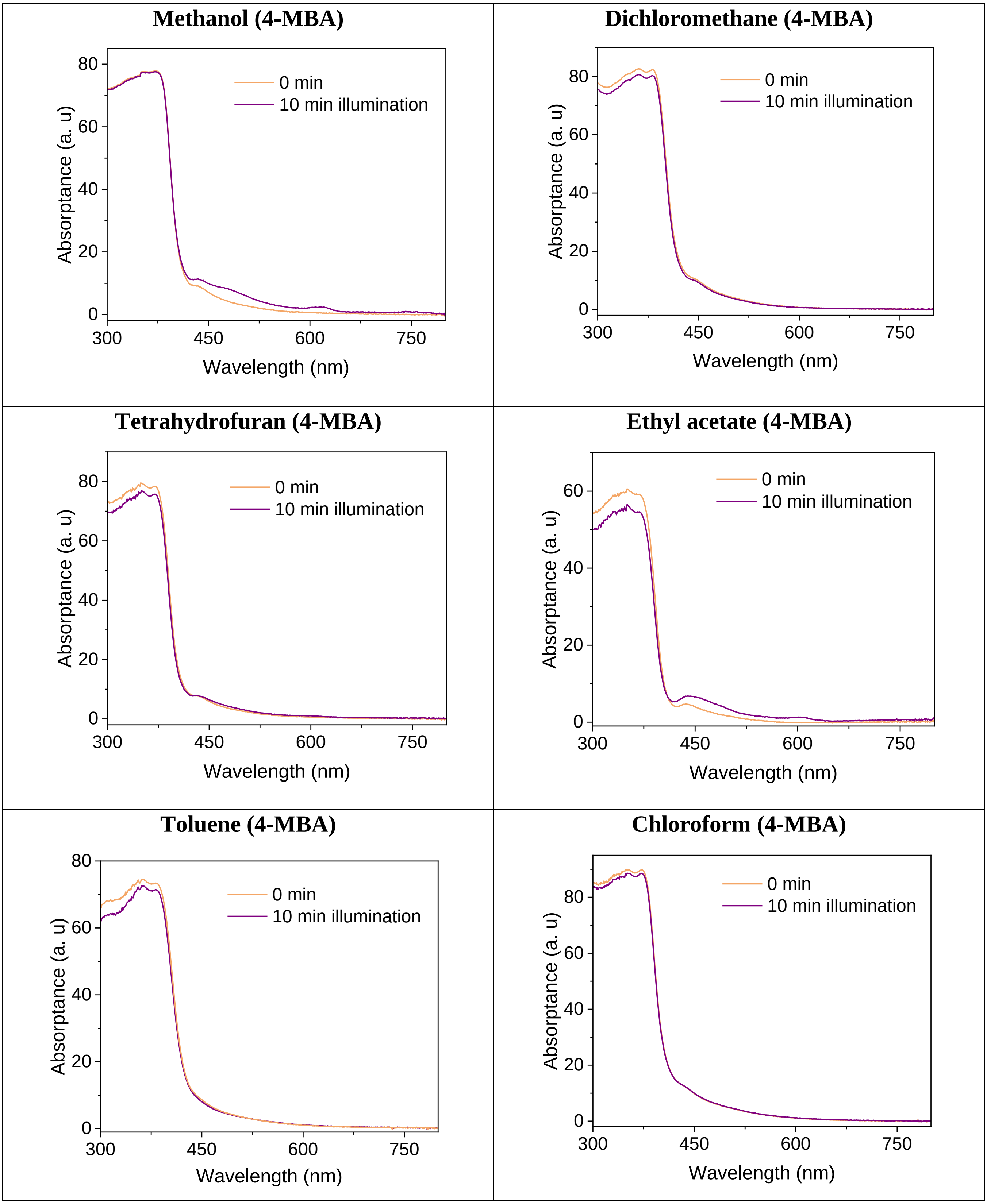

Figure S39. UV-vis absorptance spectra of NDI-COF suspension in several oxygen-free solvents (dry) in the presence of 10 mM 4-MBA as SED before and after 10 min UV illumination. These solvent-SED combinations did not result in the formation of $NDI^{\bullet -}$ radical.

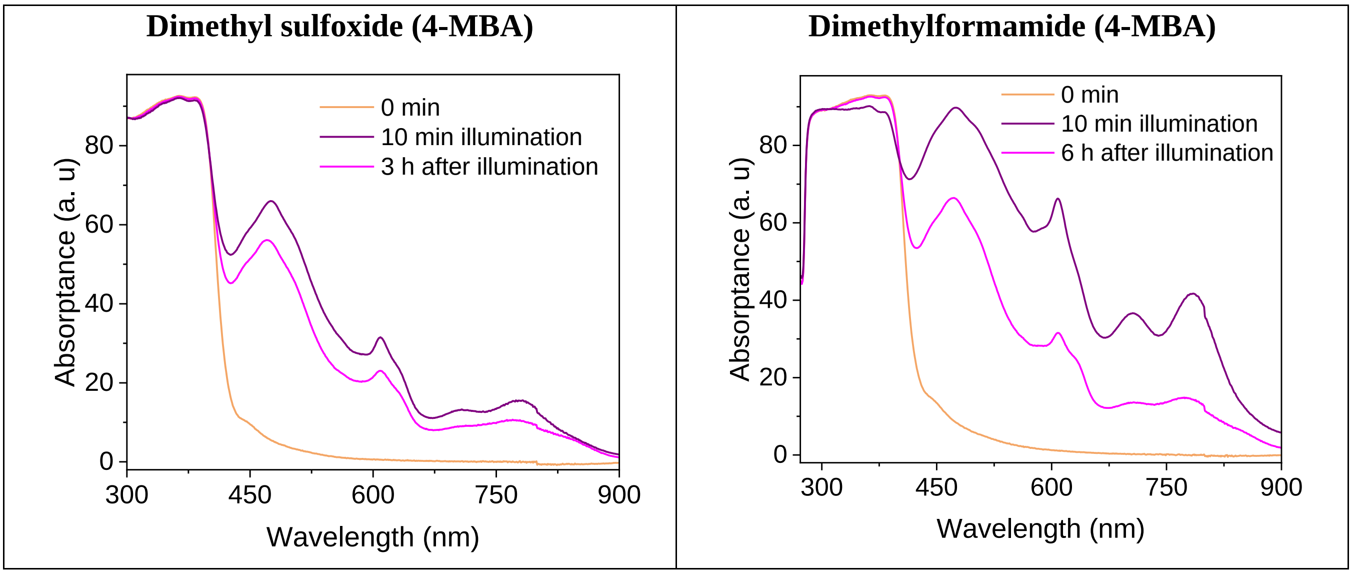

Figure S40. UV-vis absorptance spectra of NDI-COF suspension in oxygen-free dimethyl sulfoxide and dimethylformamide (dry) in the presence of 10 mM 4-MBA as SED before and after 10 min UV illumination, and dark stability measured at different post-illumination times. Note that pure dimethylformamide can also act as a weak electron donor due to the presence of amine impurities.

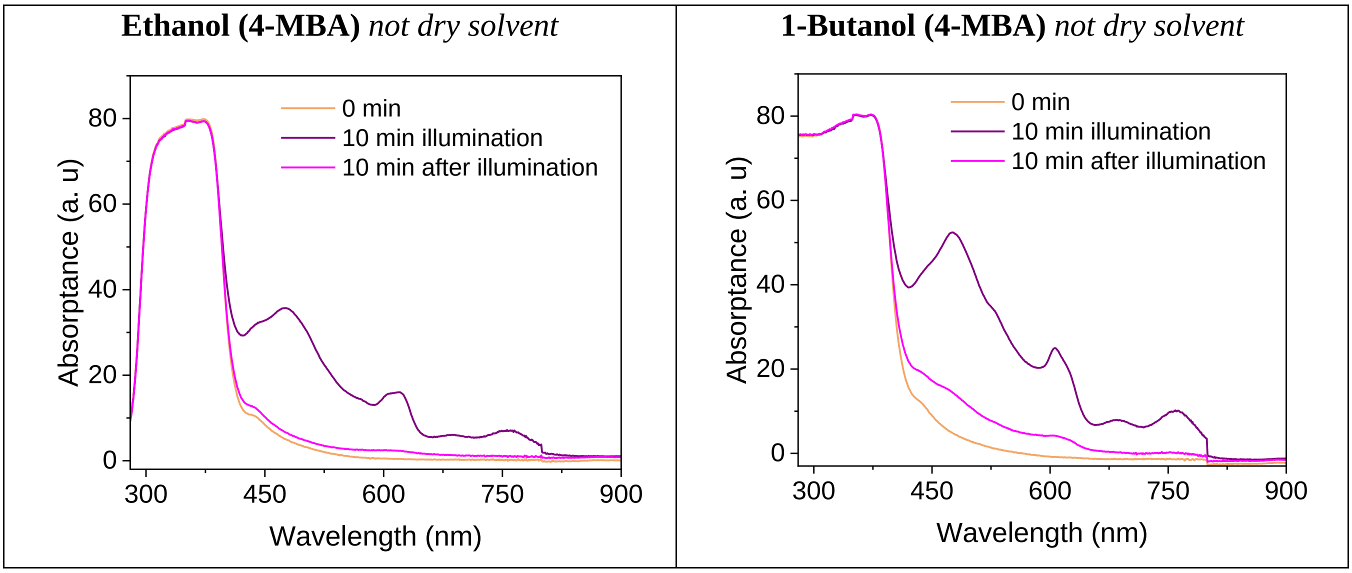

Figure S41. UV-vis absorptance spectra of NDI-COF suspension in oxygen-free ethanol and 1-butanol (not dry) in the presence of 10 mM 4-MBA as SED before, after 10 min UV illumination, and 10 minutes post-illumination. As the solvents are not dry, the presence of water possibly helps in the formation of some $NDI^{\bullet-}$ radicals. However, the stability of $NDI^{\bullet-}$ radicals does not exceed more than 10 minutes.

Table S5. Monitoring NDI$^{\bullet-}$ radical formation in NDI-COF suspension in water and acetonitrile with few electron donors. The corresponding UV-vis absorptance spectra are shown in **Figure S42−S43**.

**Solvent**	**Dielectric constant (ε)**	**NDI$^{\bullet-}$ radical stability in electron donors**				
		**4-MBA**	**TEA**	**TEOA**	**DMA**	**DIPEA**
Water	80.10	> 48 h	> 48 h	> 48 h	immiscible^{+}	immiscible^{+}
Acetonitrile	37.50	X	< 6 h	< 6 h	< 10 min*	< 2 h

4-Methylbenzylalcohol (**4-MBA**), Triethylamine (**TEA**), Triethanolamine (**TEOA**), N,N-dimethylaniline (**DMA**), Diisopropylethylamine (**DIPEA**)

X − represents no NDI$^{\bullet-}$ radical formation

$^{+}$ As **DMA** and **DIPEA** are immiscible in water, they could not be used as electron donors in water.

* Partial NDI$^{\bullet-}$ radical formation (see below in **Figure S42−S43**)

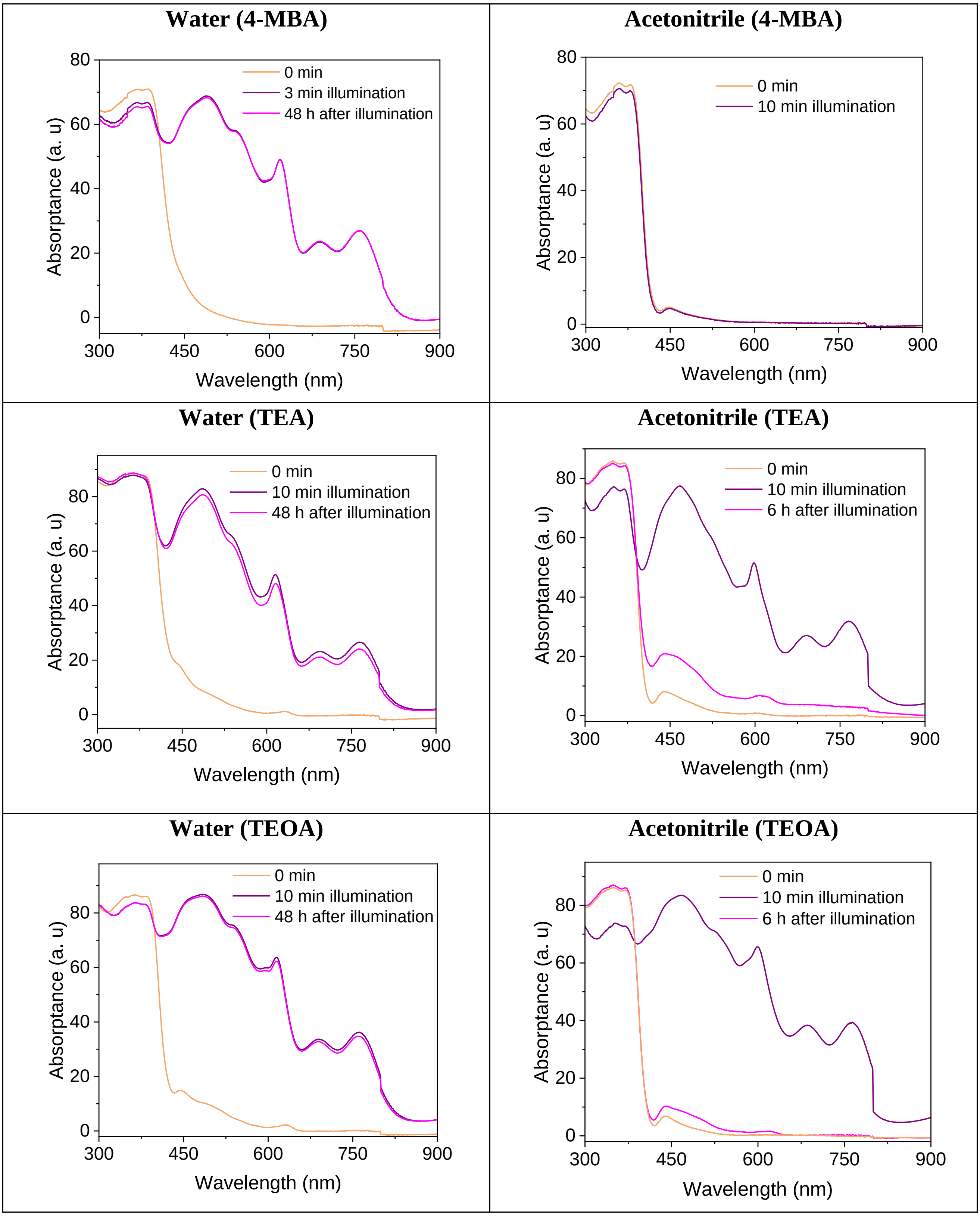

Figure S42. UV-vis absorptance spectra of NDI-COF suspension in oxygen-free water and acetonitrile (dry) in the presence of 10 mM sacrificial electron donors before, after 10 min UV illumination and their dark stability after few hours.

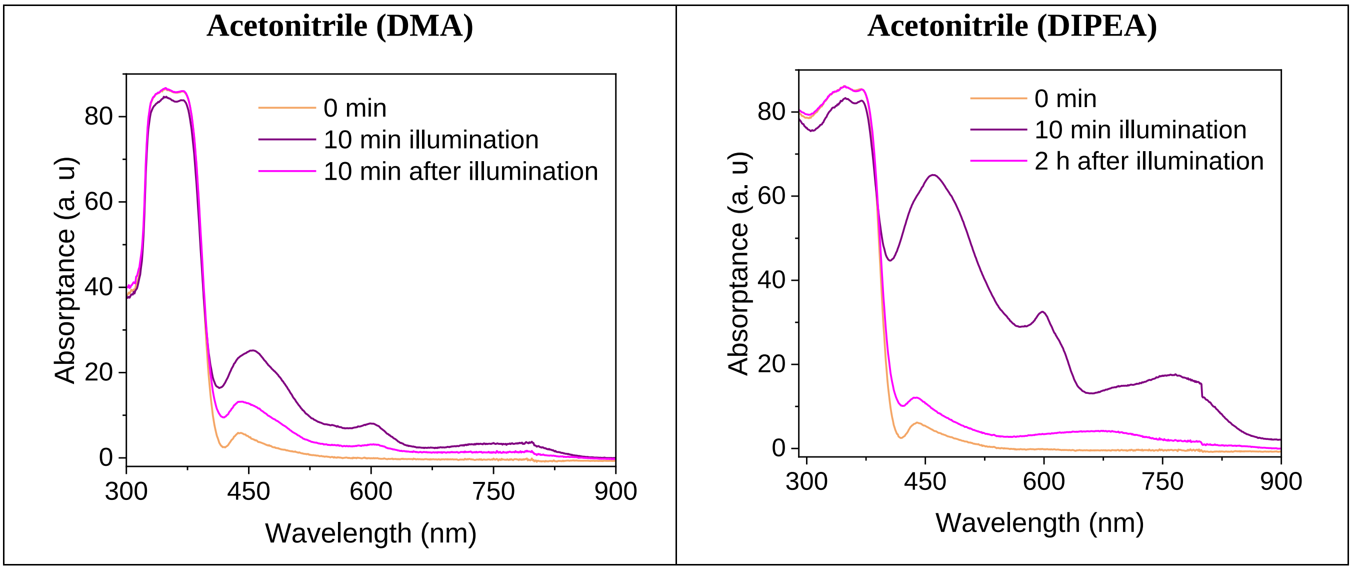

Figure S43. UV-vis absorptance spectra of NDI-COF suspension in oxygen-free acetonitrile (dry) in the presence of 10 mM sacrificial electron donors before, after 10 min UV illumination and their dark stability after few hours.

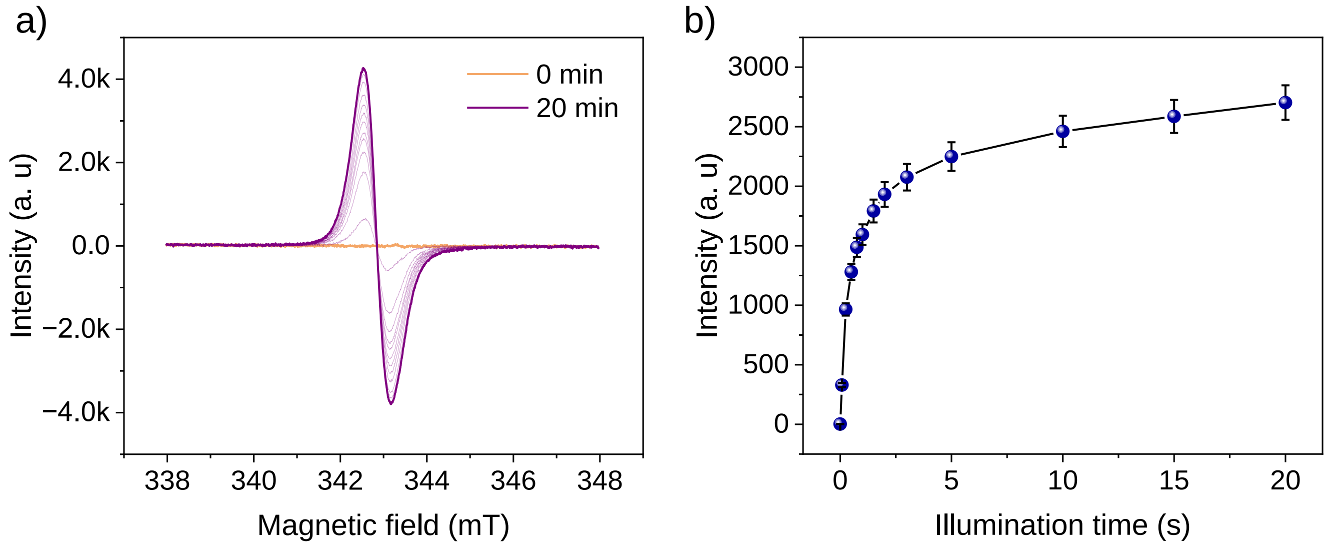

Figure S44. (a) EPR spectra of NDI-COF in acetonitrile containing 10 mM TEA shows the formation of photoinduced $NDI^{\bullet-}$ radical anion. (b) The reaction progression was monitored by the double integration of EPR signals.

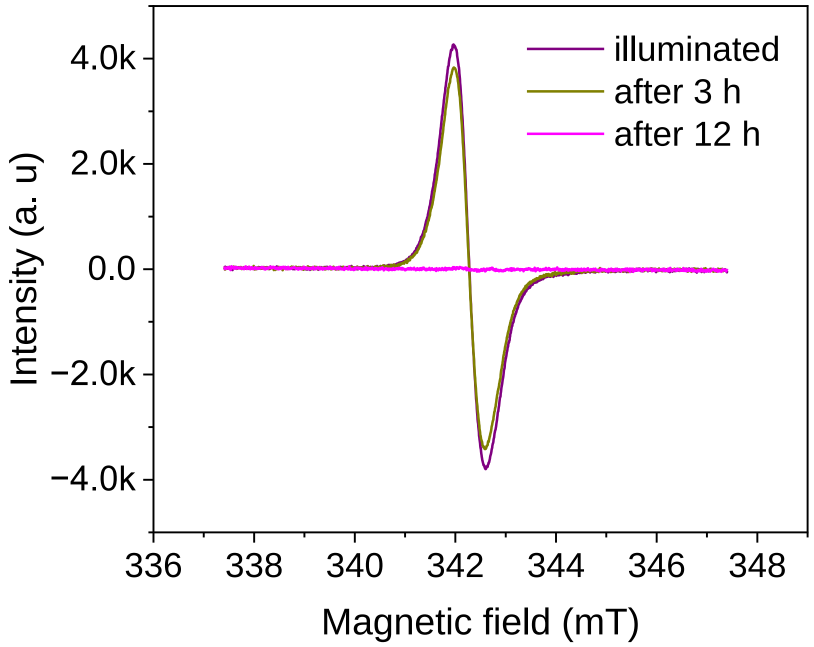

Figure S45. Time evolution of the EPR spectrum of NDI-COF in acetonitrile containing 10 mM TEA after 20 min illumination. The EPR signal for the $NDI^{\bullet-}$ radical anion diminishes over 12 h, going back to the original state.

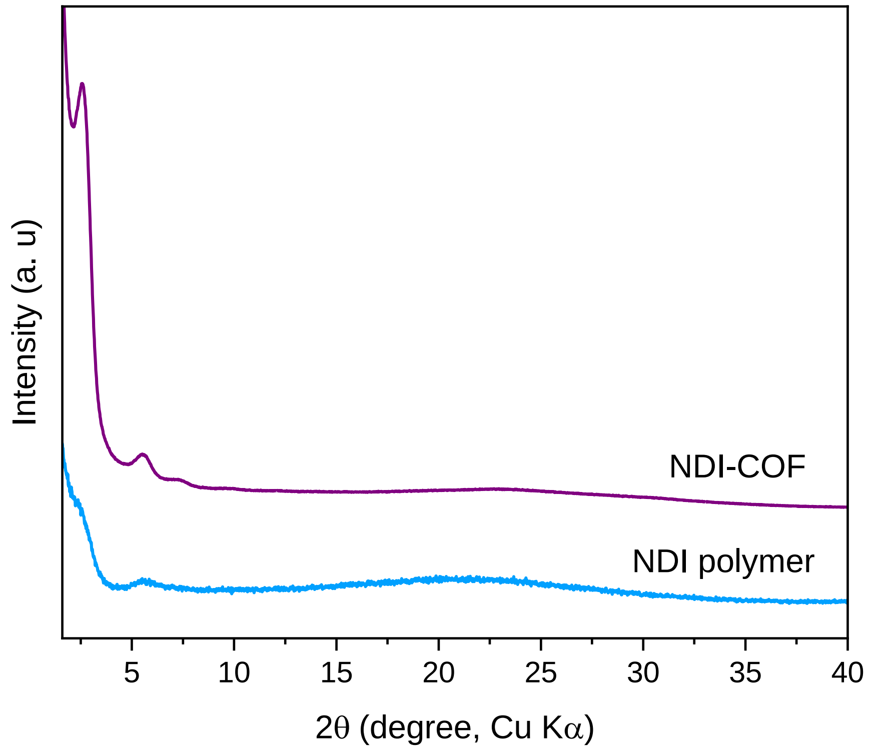

Figure S46. Comparison of PXRD patterns of NDI-COF and NDI polymer synthesized from TAPB and NTCDA.

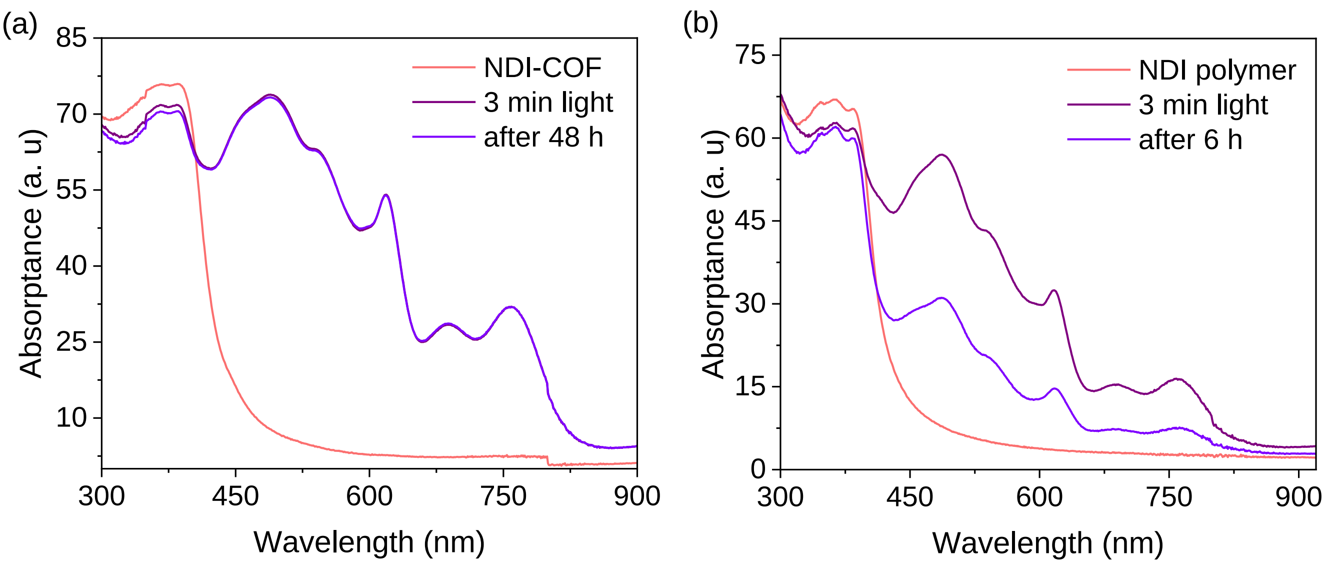

Figure S47. UV-vis absorptance spectra of (a) NDI-COF and (b) NDI polymer suspension in oxygen-free water in the presence of 10 mM 4-MBA (sacrificial electron donor) before and after UV illumination, and their dark stability after several hours. The stability of $NDI^{\bullet-}$ radical anion significantly decreases in NDI polymer as compared to NDI-COF.

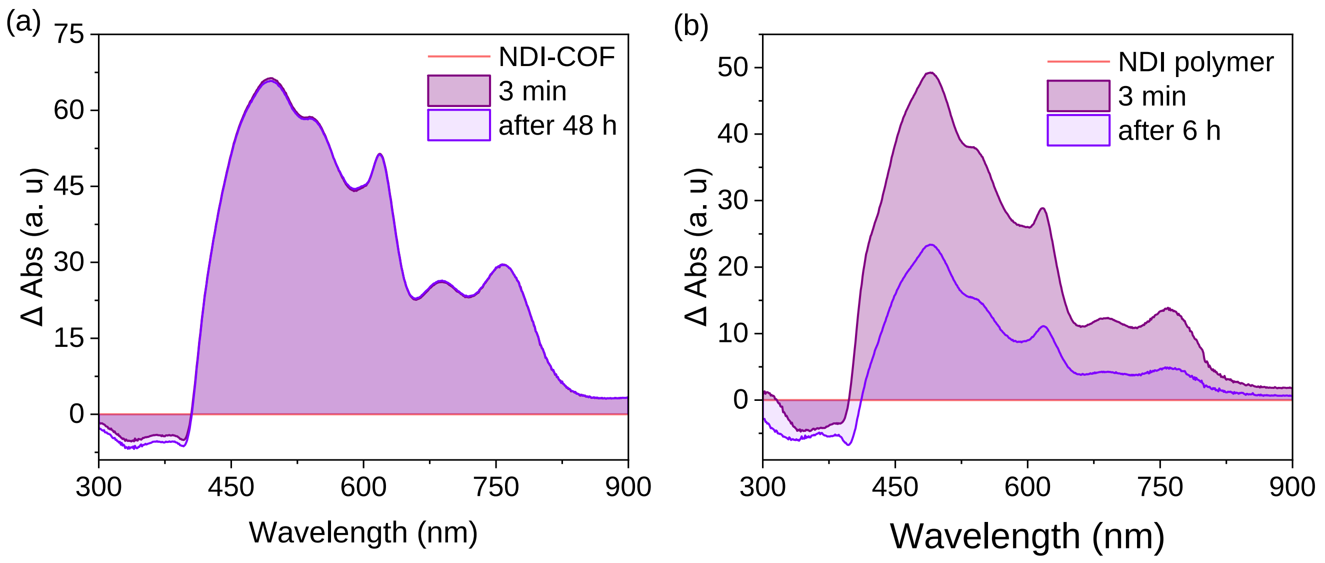

Figure S48. Change in UV-vis absorptance spectra of (a) NDI-COF and (b) NDI polymer suspension in oxygen-free water in the presence of 10 mM 4-MBA (sacrificial electron donor) before, after UV illumination and their dark stability after few hours. The stability of $NDI^{\bullet-}$ radical anion significantly decreases in NDI polymer as compared to NDI-COF.

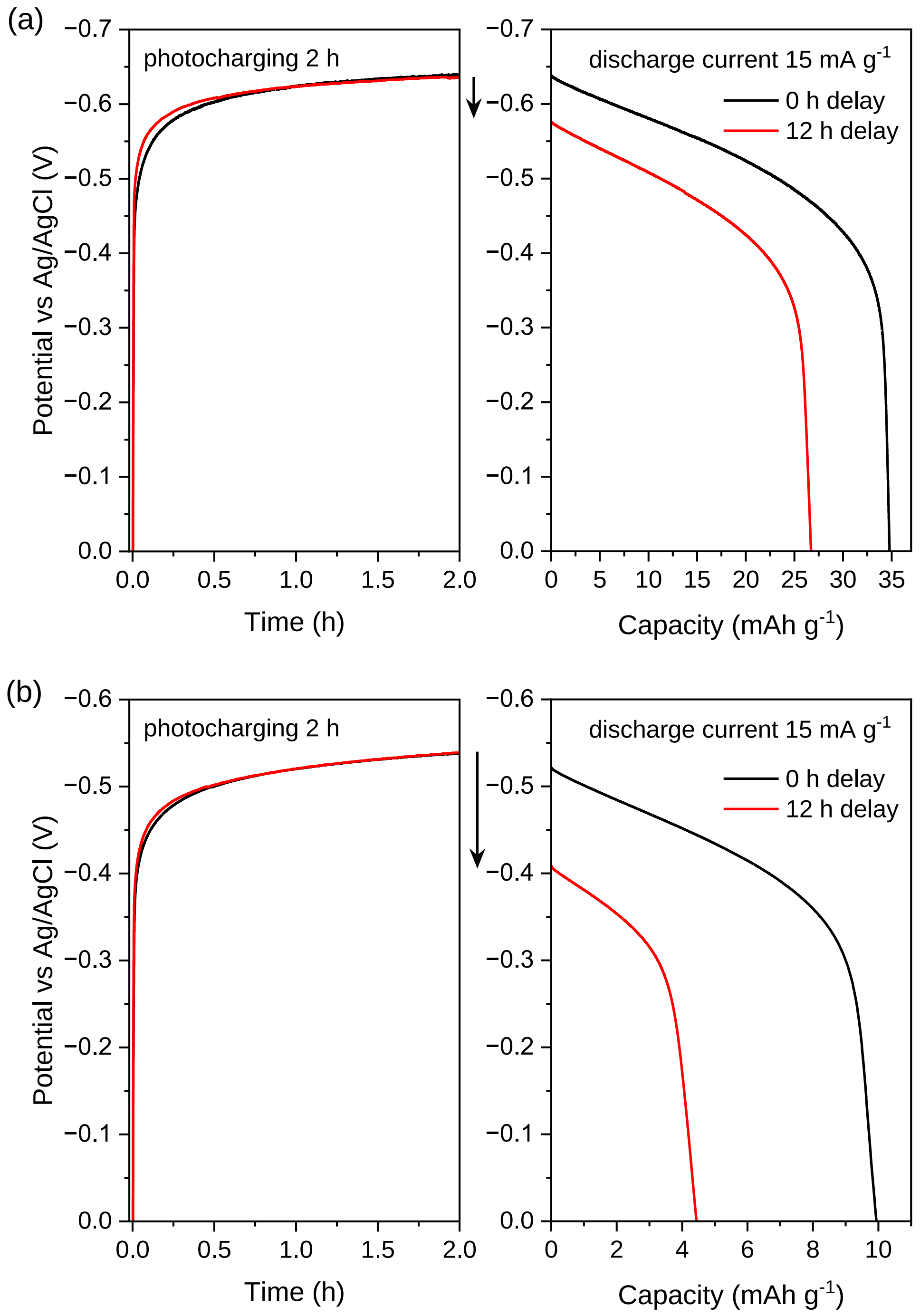

Figure S49. Charge storage in solar battery photoanode of (a) NDI-COF and (b) NDI polymer. Light-assisted charging profiles for a duration of 2 h, followed by direct and delayed (representative 12 h) electric discharging in the dark. After 2 h photocharging, the dark stability was tested for 12 h before applying a discharge current density of 15 mA g^{-1} in oxygen free 10 mM 4-MBA aqueous electrolyte.

References

(1) Stoll, S.; Schweiger, A. EasySpin, a comprehensive software package for spectral simulation and analysis in EPR. *J. Magn. Reson.* **2006**, *178* (1), 42-55.

(2) Heyd, J.; Scuseria, G. E.; Ernzerhof, M. Hybrid functionals based on a screened Coulomb potential. *J. Chem. Phys.* **2003**, *118* (18), 8207-8215.

(3) Kühne, T. D.; Iannuzzi, M.; Del Ben, M.; Rybkin, V. V.; Seewald, P.; Stein, F.; Laino, T.; Khaliullin, R. Z.; Schütt, O.; Schiffmann, F. CP2K: An electronic structure and molecular dynamics software package-Quickstep: Efficient and accurate electronic structure calculations. *J. Chem. Phys.* **2020**, *152* (19).

(4) Goedecker, S.; Teter, M.; Hutter, J. Separable dual-space Gaussian pseudopotentials. *Phys. Rev. B* **1996**, *54* (3), 1703.

(5) Hartwigsen, C.; Gœdecker, S.; Hutter, J. Relativistic separable dual-space Gaussian pseudopotentials from H to Rn. *Phys. Rev. B* **1998**, *58* (7), 3641.

(6) Krack, M. Pseudopotentials for H to Kr optimized for gradient-corrected exchange-correlation functionals. *Theor. Chem. Acc.* **2005**, *114*, 145-152.

(7) Runge, E.; Gross, E. K. Density-functional theory for time-dependent systems. *Phys. Rev. Lett.* **1984**, *52* (12), 997.

(8) Casida, M. E.; Jamorski, C.; Casida, K. C.; Salahub, D. R. Molecular excitation energies to high-lying bound states from time-dependent density-functional response theory: Characterization and correction of the time-dependent local density approximation ionization threshold. *J. Chem. Phys.* **1998**, *108* (11), 4439-4449.

(9) Casida, M. E.; Casida, K. C.; Salahub, D. R. Excited-state potential energy curves from time dependent density-functional theory: A cross section of formaldehyde's 1A1 manifold. *Int. J. Quantum Chem.* **1998**, *70* (4-5), 933-941.

(10) Vasiliev, I.; Öğüt, S.; Chelikowsky, J. R. Ab initio excitation spectra and collective electronic response in atoms and clusters. *Phys. Rev. Lett.* **1999**, *82* (9), 1919.

(11) Benedict, L. X.; Shirley, E. L.; Bohn, R. B. Optical absorption of insulators and the electron-hole interaction: An ab initio calculation. *Phys. Rev. Lett.* **1998**, *80* (20), 4514.

(12) Laurent, A. D.; Jacquemin, D. TD-DFT benchmarks: a review. *Int. J. Quantum Chem.* **2013**, *113* (17), 2019-2039.

(13) Mukamel, S. Principles of Nonlinear Optical Spectroscopy. Oxford University Press: 1995.

(14) Köuppel, H.; Domcke, W.; Cederbaum, L. S. Multimode molecular dynamics beyond the Born-Oppenheimer approximation. *Adv. Chem. Phys.* **1984**, 59-246.

(15) Köppel, H. Diabatic representation: Methods for the construction of diabatic electronic states. In *Conical intersections: electronic structure, dynamics and spectroscopy*, World Scientific, 2004; pp 175-204.
(16) Worth, G. A.; Cederbaum, L. S. Beyond Born-Oppenheimer: molecular dynamics through a conical intersection. *Annu. Rev. Phys. Chem.* **2004**, *55* (1), 127-158.
(17) Kresse, G.; Joubert, D. From ultrasoft pseudopotentials to the projector augmented-wave method. *Phys. Rev. B* **1999**, *59* (3), 1758.
(18) Perdew, J. P.; Burke, K.; Ernzerhof, M. Generalized gradient approximation made simple. *Phys. Rev. Lett.* **1996**, *77* (18), 3865.
(19) Grimme, S.; Ehrlich, S.; Goerigk, L. Effect of the damping function in dispersion corrected density functional theory. *J. Comput. Chem.* **2011**, *32* (7), 1456-1465.
(20) Royuela, S.; Martínez-Periñán, E.; Arrieta, M. P.; Martínez, J. I.; Ramos, M. M.; Zamora, F.; Lorenzo, E.; Segura, J. L. Oxygen reduction using a metal-free naphthalene diimide-based covalent organic framework electrocatalyst. *Chem. Commun.* **2020**, *56* (8), 1267-1270.
(21) Podjaski, F.; Kroger, J.; Lotsch, B. V. Toward an Aqueous Solar Battery: Direct Electrochemical Storage of Solar Energy in Carbon Nitrides. *Adv. Mater.* **2018**, *30* (9), 1705477.
(22) Stanley, P. M.; Sixt, F.; Warnan, J. Decoupled Solar Energy Storage and Dark Photocatalysis in a 3D Metal-Organic Framework. *Adv. Mater.* **2023**, *35* (1), e2207280.
(23) Wu, S.; Stanley, P. M.; Deger, S. N.; Hussain, M. Z.; Jentys, A.; Warnan, J. Photochargeable Mn-Based Metal-Organic Framework and Decoupled Photocatalysis. *Angew. Chem. Int. Ed.* **2024**, e202406385.
(24) Wang, Y.; Chan, Y. T.; Oshima, T.; Duppel, V.; Bette, S.; Kuster, K.; Gouder, A.; Scheurer, C.; Lotsch, B. V. Decoupling of Light and Dark Reactions in a 2D Niobium Tungstate for Light-Induced Charge Storage and On-Demand Hydrogen Evolution. *J. Am. Chem. Soc.* **2024**, *146* (37), 25467-25476.
